# Time to Definitive Health-Related Quality of Life Score Deterioration in Patients with Resectable Metastatic Colorectal Cancer Treated with FOLFOX4 versus Sequential Dose-Dense FOLFOX7 followed by FOLFIRI: The MIROX Randomized Phase III Trial

**DOI:** 10.1371/journal.pone.0157067

**Published:** 2016-06-16

**Authors:** Zeinab Hamidou, Benoist Chibaudel, Mohamed Hebbar, Marine Hug de Larauze, Thierry André, Christophe Louvet, David Brusquant, Marie-Line Garcia-Larnicol, Aimery de Gramont, Franck Bonnetain

**Affiliations:** 1 National clinical research Platform for Quality of life in Oncology, Marseille, France; 2 Public health laboratory, College of Medicine, Marseille, France; 3 Saint-Antoine Hospital, Department of Medical Oncology, Paris, France; 4 Centre Hospitalier Régional Universitaire, Claude Huriez Hospital, Service of Medical Oncology, Lille, France; 5 Groupe Coopérateur Multidisciplinaire en Oncologie (GERCOR), Paris, France; 6 Institut Mutualiste Montsouris, Department of Medical Oncology, Paris, France; 7 University Hospital of Besançon, Methodology and quality of life unit in oncology (EA3181), Besançon, France; University Campus Bio-Medico, ITALY

## Abstract

**Purpose:**

We previously showed that a sequential chemotherapy with dose-dense oxaliplatin (FOLFOX7) and irinotecan (FOLFIRI; irinotecan plus 5-fluorouracil/leucovorin) is not superior to FOLFOX4 in patients at advanced stage of colorectal cancer with liver metastases. Here we aimed to determine whether time to health-related quality of life (HRQoL) score definitive deterioration (TUDD) differs by study arm.

**Methods:**

HRQoL was evaluated using the European Organization for Research and Treatment of Cancer (EORTC) QLQ-C30 at baseline and every 4 cycles until the end of the study or death. Functional scale, symptom scale, global health status, and financial difficulties were analyzed. The TUDD was defined as the time interval between randomization and the first decrease in HRQoL score ≥ 5-point with no further improvement in HRQoL score ≥ 5 points or any further HRQoL data. TUDD was estimated using the Kaplan-Meier method and the long-rank test. Cox regression analyses were used to identify HRQoL items influencing TUDD. Sensitivity analyses were done using a multiple imputation method and different definitions of TUDD.

**Results:**

Of the 284 patients, 171 (60.2%) completed HRQoL questionnaires. Cox multivariate analysis showed no statistically significant difference in TUDD for most of the QLQ-C30 scales between treatments. Patients with dyspnea and those without symptoms at baseline had a significantly longer TUDD when there was a delay >12 months between diagnosis of the primary tumor and metastases (HR 0.48 [0.26–0.89]) and when there was diarrhea (HR 0.59 [0.36–0.96]), respectively.

**Conclusion:**

This study shows that TUDD does not differ significantly according to type of treatment. The TUDD method produces meaningful longitudinal HRQoL results that may facilitate effective clinical decision making in patients with mCRC.

**Trial Registration:**

ClinicalTrials.gov NCT00268398

## Introduction

Colorectal cancer (CRC) is the third leading cause of cancer death worldwide [1]. Almost 50% of patients with CRC will present metastases at same stage of their disease [2], the main cause of mortality associated to the cancer.

In case of isolated CRC metastases (mainly to the liver), the complete resection is the cornerstone treatment, allowing longer survival or even cure. Resection of metastases is possible in only 15%-25% of patients with 5-year survival rates between 25% and 65% in the most recent evaluations [3–5]. Unfortunately, majority of patients (50%-75%) will relapse at 2 years.

Additional systemic chemotherapy is increasingly used to reduce the risk of relapse. Based on the results of EORTC trial 40983, the standard of care for CRC patients is a perioperative chemotherapy composed of 12 cycles (6 cycles before and 6 cycles after surgery) of FOLFOX4 (oxaliplatin 85 mg/m^2^) [6]. In order to reduce the risk of the oxaliplatin-related neuropathy and to increase the efficacy, a modified schedule containing 6 cycles of FOLFOX7 (oxaliplatin 130 mg/m^2^) followed by 6 cycles of FOLFIRI (irinotecan plus 5-fluorouracil/leucovorin) was assessed [7]. We previously compared in phase III MIROX trial the modified schedule to 12 cycles of FOLFOX4 in patients with resectable metastatic CRC (mCRC). Chemotherapy was either perioperative or postoperative regarding the patient and disease characteristics (especially synchronicity of metastases). A sequential chemotherapy with FOLFOX7-FOLFIRI was not superior to FOLFOX4 in our study.

The importance of health-related quality of life (HRQoL) is well recognized, particularly in patients with advanced cancer. Still, HRQoL results remain poorly used to modify therapeutic strategies, mostly due to the complexity of longitudinal analysis and the lack of standardization, which lead to the inability to propose clinically meaningful HRQoL data. Moreover, in clinical studies of advanced-stage disease missing data (often arising when patients miss visits or do not fill in certain questionnaires due to rapid deterioration or death) is an important problem that potentially hampers the interpretation of HRQoL results [8].

Time until definitive deterioration (TUDD) in QoL score has been defined as a method of longitudinal analysis in oncology [9–12]. In metastatic setting, this method was allows patients’ data to be preserved for analysis even if some of their questionnaires are missing and allows producing clinically meaningful and readable results for clinicians such as a Kaplan-Meier survival curve and a hazard ratio (HR).

The aims of this analysis were to compare TUDD for QLQ-C30 scales between the FOLFOX7-FOLFIRI and FOLFOX4 arms and to investigate the applicability of this method for mCRC analysis using a subset of patients enrolled on the MIROX study.

## Patients and Methods

### Patients and eligibility criteria

Eligibility criteria and study design have been previously described elsewhere [7]. This was an open-label, randomized, phase III trial comparing the efficacy of FOLFOX7-FOLFIRI with FOLFOX4 in mCRC from 19 French centers. Patients were eligible if they had histologically confirmed colorectal adenocarcinoma with initially resectable/resected metastases in only one site (liver, lung, ovary, or peritoneum). Regarding peritoneum was allowed a single and completely resected metastasis discovered during the resection of the primary tumor. Except this case, there was no cut-off limit for the number of metastases. Other eligibility criteria included age 18–75 years, WHO performance status ≤ 2, adequate hematological, renal, and hepatic functions. Prior adjuvant chemotherapy for CRC was allowed if ended 12 months before relapse. Patients were randomized (1:1) with a minimization technique stratifying them by chemotherapy timing: perioperative versus postoperative, local intervention: surgery versus radiofrequency ablation (RFA) with/without surgery, and Fong’s score: 0–1 versus 2–3 versus 4–5. Patients received either 12 FOLFOX4 cycles (oxaliplatin dose: 85 mg/m^2^) or 6 FOLFOX7 cycles (oxaliplatin dose: 130 mg/m^2^), followed by 6 FOLFIRI cycles (irinotecan dose: 180 mg/m^2^), 1 cycle every 2 weeks.

All patients were fully informed of the study and provided signed written informed consent. The protocol was approved by the ethics committees of Lille, France (“Comité de Protection des Personnes”). This study MIROX (Combination Chemotherapy in Treating Patients with Colorectal Cancer and Resectable Metastases) was registered on ClinicalTrials.gov (Identifier: NCT00268398). The protocol for this trial (including the written informed consent form and the list of Ethics Committees) and supporting CONSORT checklist are available as supporting information (S1 and S2 Protocols and S1 Checklist).

The primary endpoint was 2-year disease-free survival (DFS) and analysis was conducted by intend to treat (ITT). Secondary endpoints were overall survival (OS), objective response rate (ORR), resection type (R0–R2), toxicity, and HRQoL. TUDD approach was used for HRQoL longitudinal analysis. Between May 2004 and June 2010, 284 patients were enrolled.

### HRQoL assessment

HRQoL was assessed using the European Organization for Research and Treatment of Cancer (EORTC) QLQ-C30 [13]. Assessment was performed at baseline (week before randomization) and every 4 cycles thereafter. The QLQ-C30 is a 30-item cancer-specific tool that generates global health status (GHS), five functional scales (physical, role, emotional, cognitive, and social), eight symptom scales (fatigue, nausea/vomiting, pain, dyspnea, insomnia, anorexia, constipation, diarrhea), and one financial difficulties item. Scoring was completed according to the EORTC scoring manual. Raw scores were linearly transformed to a 0 to 100 scale. For GHS lower scores represent worst HRQoL and higher scores better HRQoL, while for symptom parameters lower scores represent better HRQoL and higher scores worst HRQoL.

### Statistical methods

The primary endpoint was DFS, defined as the interval between randomization and first evidence of relapse or death from any cause. ORR was evaluated according to the RECIST v.1.0 criteria [14]. Adverse events (AEs) were graded according to the NCI-CTCAE v.2.0 and an oxaliplatin-specific scale for neuropathy [15]

The sample size was based on the hypothesis that the 2-year DFS might be improved from 30% with FOLFOX4 chemotherapy to 45% with FOLFOX7-FOLFIRI (Hazard Ratio [HR] of 0.66). To demonstrate this 15% difference using an 80% power and bilateral α type I error of 5%, 188 events were required. Based on estimated 36 months inclusion duration, and 24 months follow-up, at least 248 patients had to be enrolled. Assuming a drop-out rate of 20% (disease progression before surgery, R2 resections or lost to follow-up), a total number of 284 patients, 142 per treatment arm, was required.

Analyses were carried out on all patients who received at least one dose of treatment, based on a mITT approach. Survival and median follow-up were estimated by the Kaplan–Meier and the reverse Kaplan–Meier method, respectively [16, 17]. Differences between treatment groups’ outcomes were compared using a log-rank test. Proportional hazard assumptions were tested using scaled Schoenfeld residuals [18].

Continuous and qualitative variables were described by means and standard deviations (SD) and medians (min-max), and percentages, respectively. Patient characteristics were described according to the completion of questionnaire at baseline in order to determine a non-random missing patient profile. Questionnaire completion rates were calculated as a percentage of all patients who completed a questionnaire at a given time point. Completion rates and baseline HRQoL scores were compared according to treatment arm. Randomized patients whatever eligibility criteria with available HRQoL scores at baseline were included in the HRQoL analyses (modified ITT analysis).

### Analysis of HRQoL

TUDD was defined as the interval between randomization and the first decrease in HRQoL score ≥ 5 points compared to baseline HRQoL score with no further improvement or in case of patient who dropped out after a ≥ 5 points decrease, resulting in missing data or death [10,19]. Alive patients were censored at the last HRQoL follow-up if a ≥ 5 points deterioration from baseline was not observed or if a ≥ 5 points decrease was present, but was followed by secondary ≥ 5 points improvement [10]. All randomly assigned patients with a baseline and at least one post-baseline HRQoL assessment were included in TUDD analyses.

TUDD was calculated using the Kaplan-Meier method and compared with the log-rank test. TUDD was described using medians with 95% confidence interval (CI). The univariate Cox model was used to calculate HR with 95% CI. The multivariate Cox model, with treatment arms and other covariates, was applied to identify independent factors associated with TUDD for each scale. All variables with a univariate P value ≤ 0.20 from the Cox univariate analyses were eligible for multivariate analyses. Correlations were tested for eligible variables. To prevent collinearity, when two variables were significantly correlated, one variable was retained according to its clinical relevance or to the value of the likelihood ratio. The treatment arm was forced into the multivariate analyses. The time to progression status was included in Cox analyses as a time-dependent variable.

### Sensitivity analyses

As only the patients with a baseline HRQoL score were considered in TUDD, sensitivity analyses were performed to evaluate the effect on results of the discarded group of patients. Multiple imputation with predictive mean matching (PMM) method was used to handle baseline missing score. PMM matches the missing value to the observed value with the closest predicted mean (or linear prediction) [20]. The TUDD analyses were repeated on multiple imputation data in the same ways as done on the original data set.

Two sensitivity analyses by definition of an event for TUDD were also performed. In the first approach death was excluded as an event from the TUDD definition. The second approach used time to deterioration (TTD) that was defined as the interval between randomization and the first 5-point decrease in HRQoL compared to baseline HRQoL [11]. No further HRQoL investigation after this deterioration was considered. Patients were censored at the time of the last HRQoL assessment if they had not deteriorated before that.

As HRQoL was a secondary endpoint of the MIROX trial, no multiplicity adjustment was performed. All tests were two-sided and analyses were performed with Stata 11 software [21].

## Results

### Patients

Between May 2004 and June 2010, 284 patients were enrolled. One hundred forty two patients received FOLFOX4 and 142 were given FOLFOX7 followed by FOLFIRI. The treatment groups were well balanced for baseline characteristics (S1 Table). Median age was 62 years, 67% of patients were male, 68% had colon cancer, and 33% had disease symptoms at baseline. Details have been given elsewhere [7].

At a median follow-up of 67.0 months (95% CI 62.2–75.4), 177 events (89 FOLFOX4, 88 FOLFOX7-FOLFIRI) were observed. The median DFS was 22.4 months in the FOLFOX4 arm (95% CI 16.5–37.5) and 24.3 months in the FOLFOX7-FOLFIRI arm (95% CI 19.3–39.9; HR = 0.94, 95% CI 0.70–1.26; P = 0.68). Death occurred in 99 patients (49 FOLFOX4, 50 FOLFOX7-FOLFIRI). The 2, 3, and 5-year OS rates were 86.5%, 80.5%, and 69.5% with FOLFOX4, and 91.5%, 81.4%, and 66.6% with FOLFOX7-FOLFIRI, respectively (HR = 1.07, 95% CI 0.68–1.70; P = 0.76).

### Baseline HRQoL score and HRQoL compliance

**Fig 1:**
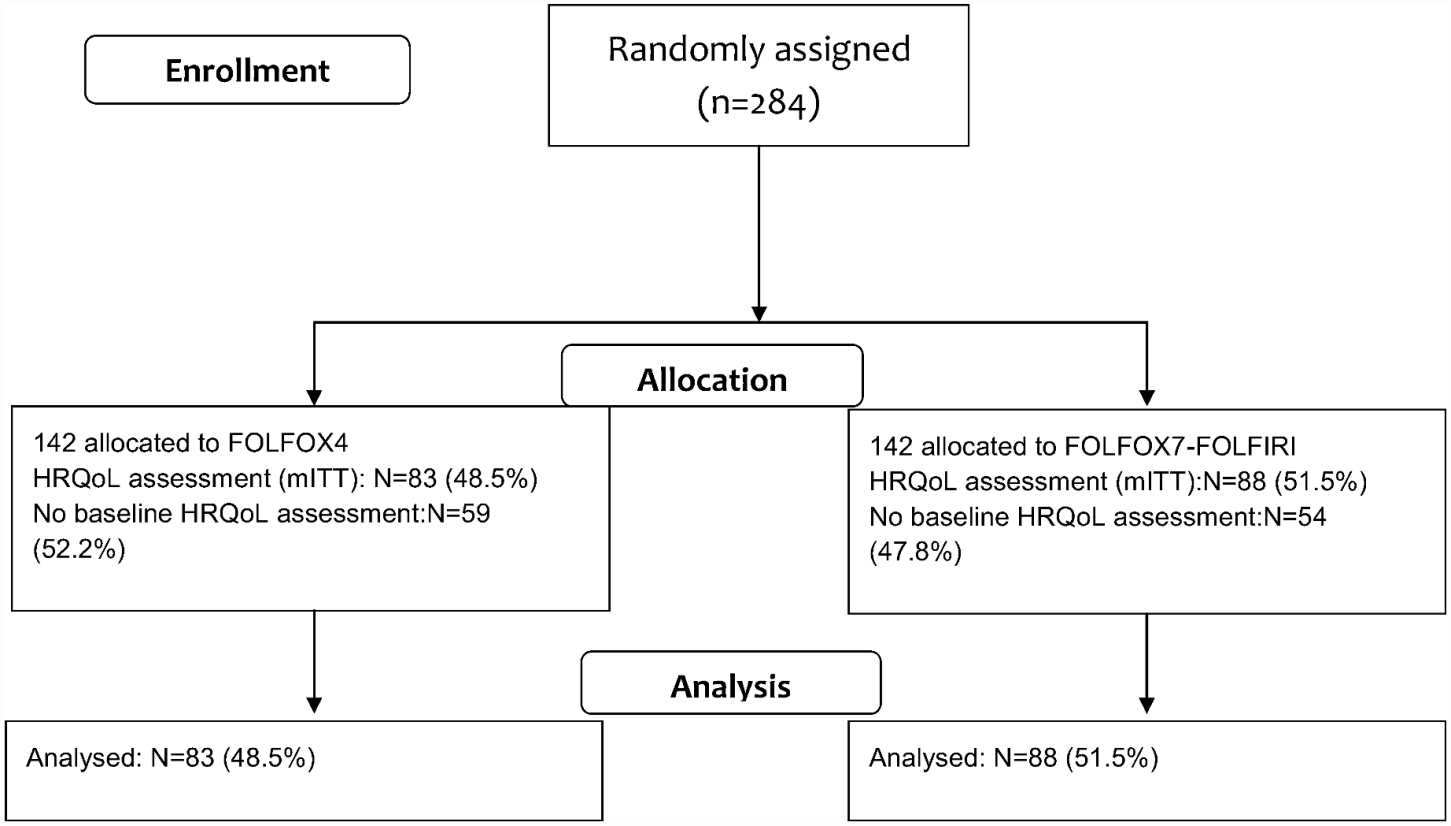
CONSORT Diagram for health-related quality of life analysis.

The compliance with HRQoL assessment is summarized in Fig 1. One hundred seventy-one (60.2%) patients completed at least one HRQoL questionnaire during the study period; 83 (48.5%) in the FOLFOX4 arm and 88 (51.5%) in the FOLFOX7-FOLFIRI arm. The differences between the number of patients who responded to the questionnaire as compared to the non-responders were as follow: 130 (76.0%) vs 141 (82.4%) at baseline, 100 (58.5%) vs 105 (61.4%) after 4 cycle, 65 (30.0%) vs 70 (40.9%) after 8 cycles, and 28 (16.3%) vs 29 (16.9%) after 12 cycles. Similar baseline characteristics, except for gender and presence of disease symptoms were observed between the two populations (S2 Table). Patients participated more likely in the HRQoL evaluation if they were women (49% vs 35% of men) and if they had no symptoms (44% vs 28% of patients with symptoms). Patients who completed HRQoL questionnaire at baseline (130; 76.0%) had similar characteristics excepted for age (Table 1). At least one score was missing in 16% of patients younger than 63 years compared to 32% of those older than 63 years.

**Table 1:** Characteristics of patients who did and did not complete the baseline questionnaire.

	Patients with complete baseline QoL questionnaire	Patients without baseline QoL assessment		
	(N = 130)	(*N* = 41)	Total	P-value*
Age, n (%)				0.02
< 63 years	75 (58)	15 (37)	90 (53)	
> = 63 years	55 (42)	26 (63)	81 (47)	
Gender, n (%)				0.84
Female	36 (28)	12 (29)	48 (28)	
Male	94 (72)	29 (71)	123 (72)	
Treatment arm, n (%)				0.75
FOLFOX4	64 (49)	19 (46)	83 (49)	
FOLFOX7 + FOLFIRI	66 (51)	22 (54)	88 (51)	
Adjuvant chemotherapy, n (%)				1
Yes	54 (42)	17 (41)	71 (42)	
No	76 (58)	24 (59)	100 (58)	
Tumor site, n (%)				0.34
Colon	90 (69)	25 (61)	115 (67)	
Rectum	40 (31)	16 (39)	56 (33)	
Body surface area, n (%)				0.7
≤1.73	36 (28)	13 (32)	49 (29)	
>1.73	92 (71)	28 (68)	120 (70)	
Unknown	2 (2)		2 (1)	
Symptoms, n (%)				1
Yes	47 (36)	15 (37)	62 (36)	
No	83 (64)	25 (61)	108 (63)	
Unknown		1	1 (1)	
Performance status, n (%)				1
0	87 (67)	28 (68)	115 (67)	
01-févr	39 (30)	12 (29)	51 (30)	
Unknown	4 (3)	1 (2)	5 (3)	
Delay between diagnosis of the primary tumor and metastasis				0.56
Simultaneous	42 (32)	11 (27)	53 (31)	
0.1–12 months	52 (40)	15 (36)	67 (39)	
> 12 months	36 (28)	15 (36)	51 (30)	

*Fisher’s exact test

The two treatment arms had similar baseline HRQoL scores in all domains except for constipation score (Table 2). Significantly higher mean constipation score was reported by patients treated with FOLFOX4 compared to those treated with FOLFOX7-FOLFIRI (19 [SD 48] vs 9 [SD 22]).

**Table 2:** Quality of life score at baseline according to treatment arm.

	FOLFOX4				FOLFOX7-FOLFIRI				
	(N = 83)				(N = 88)				
EORTC QLQ-C30 scale	N	Mean (SD)	Median	Min-Max	N	Mean (SD)	Median	Min-Max	P-value*
Global health status	69	66.6 (19.5)	66.66	8.3–100	69	73.0 (66.4)	66.6	16.6–100	0.89
Physical functioning	69	93.7 (12.0)	100	13.3–100	72	95.0 (7.4)	100	66.6–100	0.69
Role functioning	69	66.4 (54.7)	83.33	0–100	69	69.0 (32.6)	83.3	0–100	0.67
Emotional functioning	68	74.1 (22.2)	75	0–100	71	72.8 (20.6)	75	16.6–100	0.61
Cognitive functioning	69	88.8 (16.0)	100	33.3–100	71	89.6 (15.5)	100	33.3–100	0.68
Social functioning	68	83.3 (26.2)	100	0–100	70	84.5 (21.4)	100	0–100	0.79
Fatigue	69	31.9 (25.7)	33.33	0–100	71	30.4 (25.5)	33.3	0–100	0.86
Nausea	69	6.0 (13.6)	0	0–83.3	71	6.8 (16.3)	0	0–83.3	0.85
Pain	69	24.3 (49.9)	16.66	0–100	71	16.4 (22.7)	0	0–100	0.4
Dyspnea	68	15.6 (21.1)	0	0–66.6	69	16.4 (24.6)	0	0–100	0.87
Insomnia	69	28.0 (25.9)	33.33	0–66.6	71	35.2 (32.7)	33.3	0–100	0.28
Appetite loss	68	12.2 (27.5)	0	0–100	70	13.3 (26.8)	0	0–100	0.55
Constipation	69	18.8 (47.6)	0	0–100	70	9.0 (21.9)	0	0–100	0.04
Diarrhea	69	27.5 (70.0)	0	0–100	67	14.4 (24.0)	0	0–100	0.58
Financial difficulties	67	7.9 (17.4)	0	0–66.6	70	11.9 (27.2)	0	0–100	0.78

*Man and Whitney

### Univariate and multivariate analyses of TUDD

**Fig 2:**
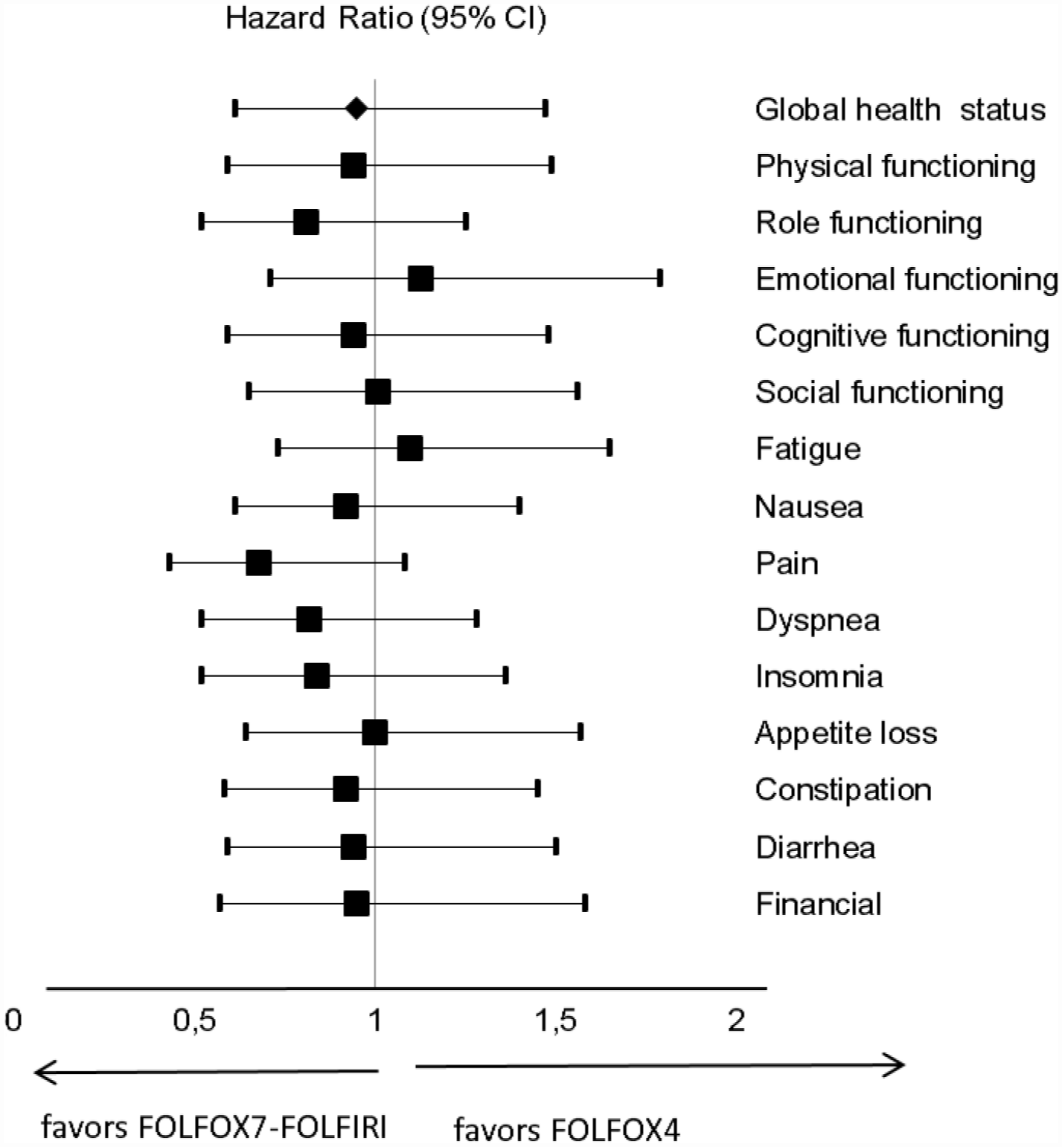
Forest plots showing hazard ratios obtained by univariate Cox regression analysis of TUDD for EORTC QLQ-C30 scales according to treatment arm.

**Fig 3:**
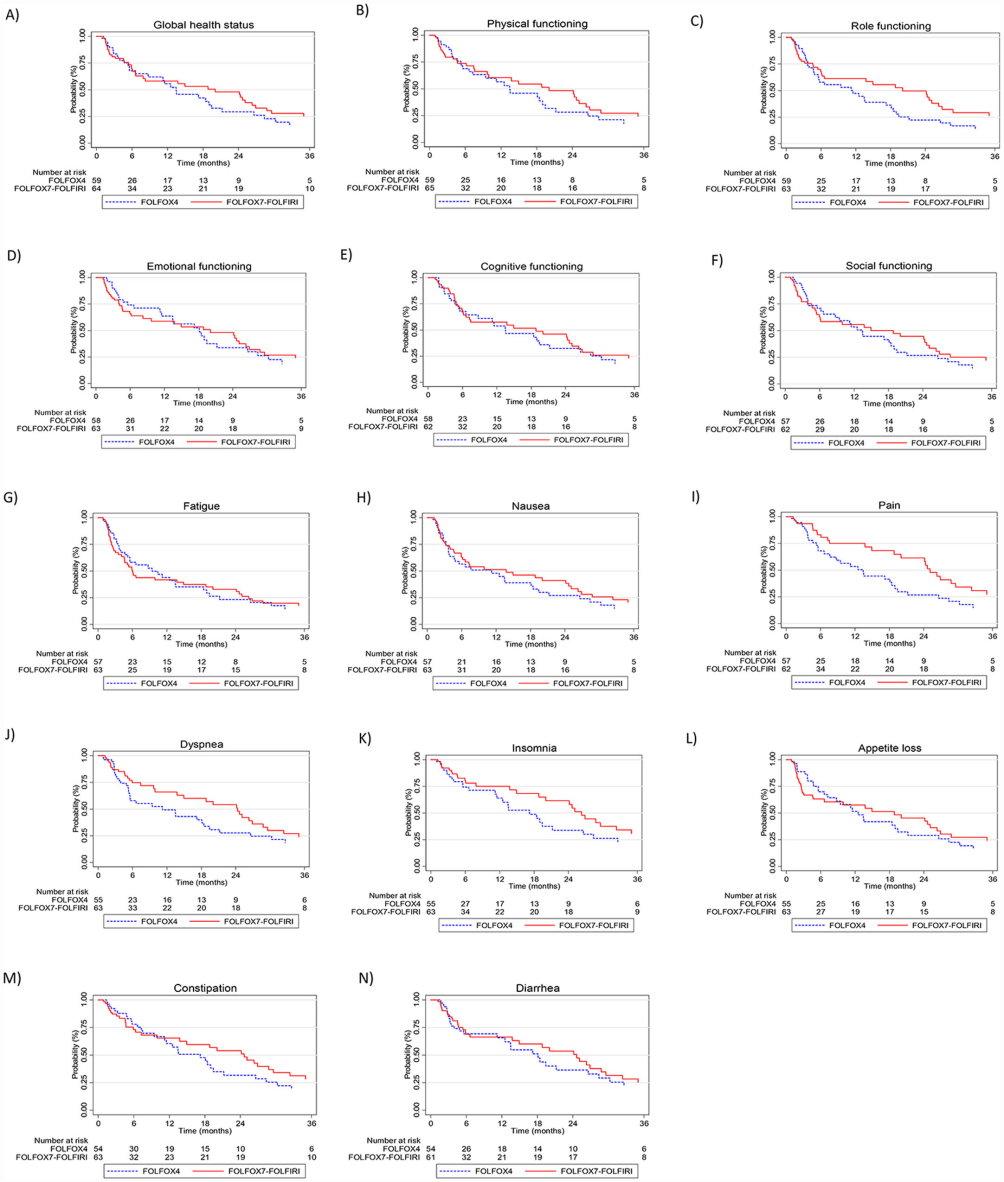
Kaplan-Meier survival curves for TUDD according to global health status (A), physical functioning (B), role functioning (C), emotional functioning (D), cognitive functioning (E), social functioning (F) fatigue (G), nausea (H), pain (I), dyspnea (J), insomnia (K), appetite loss (L), constipation (M), and diarrhea (N).

The results of univariate Cox regression analysis of TUDD (Fig 2) showed no statistically significant difference between treatments for the studied scales. Median TUDDs for the studied dimensions were also not statistically different (P *=* 0.822) according to type of treatments (Fig 3A–3N). For example, for GHS score, the median TTD was 13.4 months (95% IC: 6.6–19.4) for patients treated with FOLFOX4 (n = 37) and 20 months (95% IC: 6.6–26.3) for patients treated with FOLFOX7-FOLFIRI (n = 46; Fig 3A), with the univariate HR of 0.95 [0.61–1.47; Fig 2).

**Fig 4:**
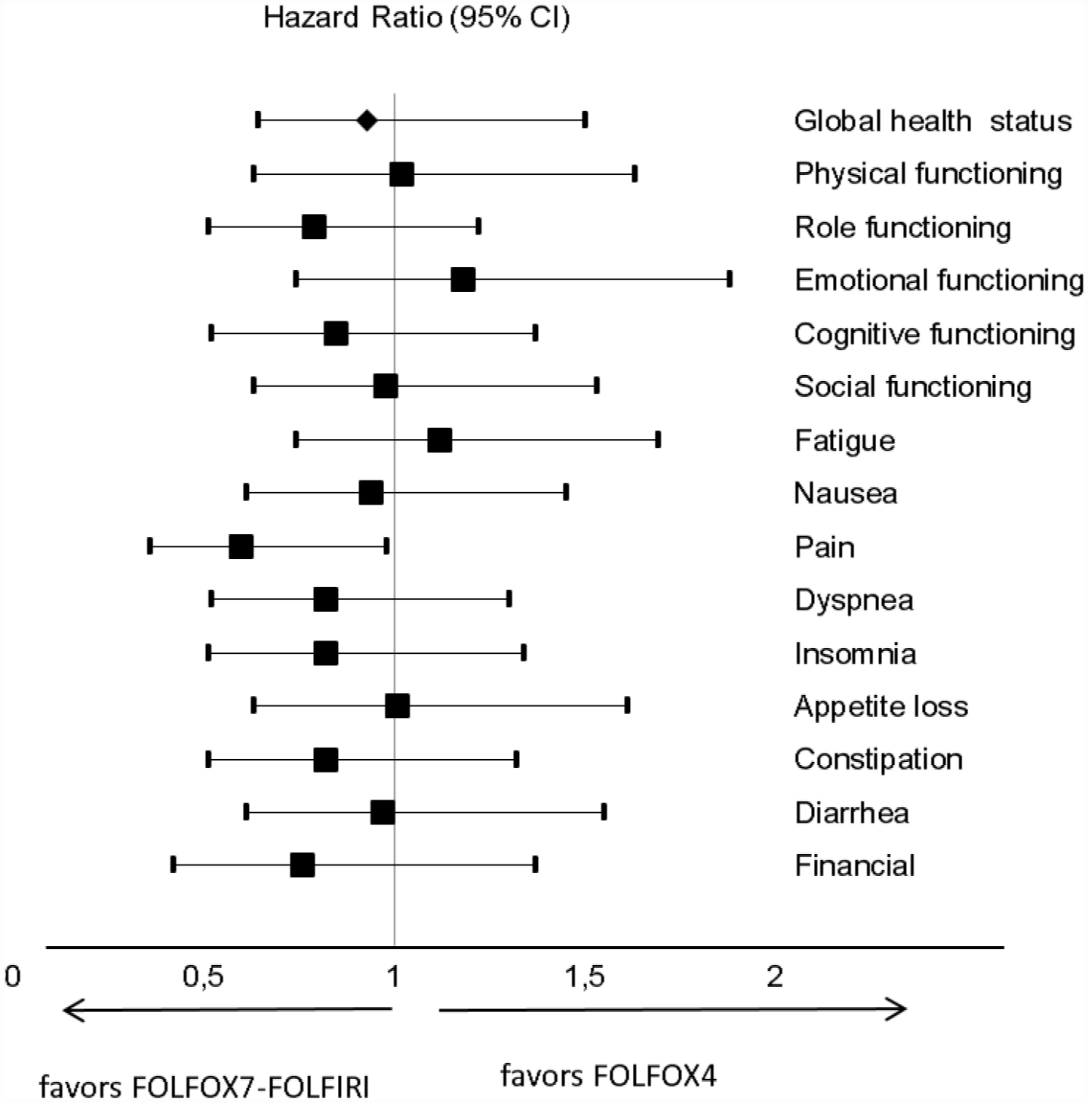
Forest plots showing hazard ratios obtained by multivariate Cox regression analysis of TUDD for EORTC QLQ-C30 scales according to treatment arm.

Multivariate Cox analyses (Fig 4 and S3 Table) showed no statistically significant difference in TUDD for the studied scales of the QLQ-C30 between treatments, except three dimensions. TUDD of pain was significantly associated with treatment arm (P *=* 0.044). Patient treated with FOLFOX7-FOLFIRI had a significantly longer TUDD (40% increase). Dyspnea was significantly associated with a delay > 12 months between diagnosis of the primary tumor and metastases (metachronous), the univariate HR of 0.48 [0.26–0.89]. Patients who did not have symptoms at inclusion had a significantly longer TUDD for diarrhea (HR 0.59 [0.36–0.96]).

### Sensitivity analyses

**Fig 5:**
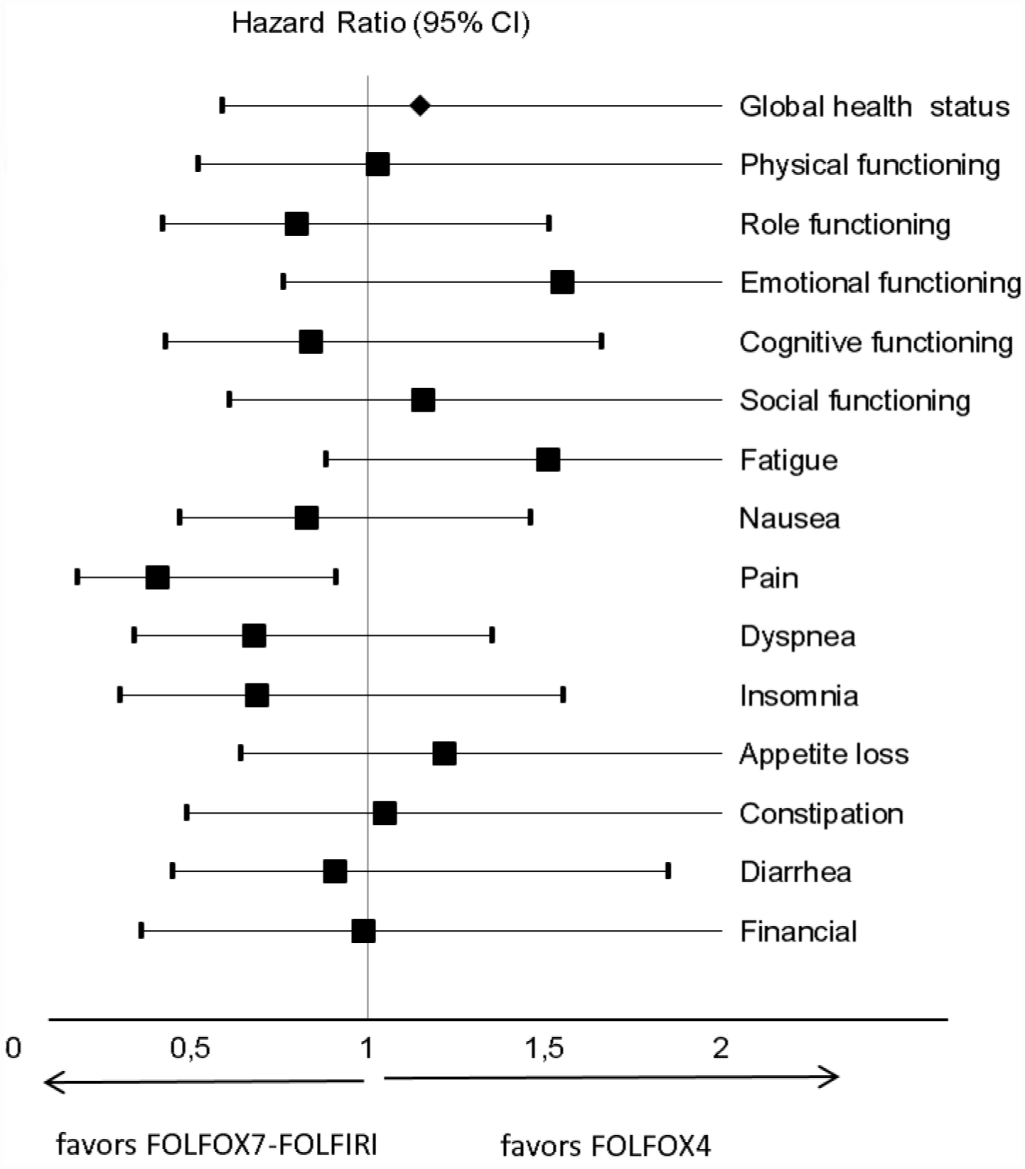
Forest plots showing hazard ratios obtained by multivariate Cox regression analysis of TUDD (excluding death as event) for EORTC QLQ-C30 scales according to treatment arm.

In univariate sensitivity analysis of TUDD excluding death as event (Fig 5, S4 Table), the TUDD did not differ significantly according to type of treatment. In addition, this result was statistically very similar to result from TTD analysis. Of note, the benefit of FOLFOX7-FOLFIRI treatment compared to FOLFOX4 treatment yielded a near-significant difference for pain (HR 0.49 [0.22–1.06]) in TUDD analysis and reached statistical significance in TTD analysis (HR 0.41 [0.18–0.91]; S3 Table).

In multivariate Cox analysis using multiple imputation data (not shown), TUDD of pain symptom was no longer significantly associated with treatment arm (HR 0.7 [0.44–1.10] for FOLFOX7-FOLFIRI, P = 0.13 compared to FOLFOX4.

## Discussion

HRQoL has become an increasingly important treatment outcome in the care of cancer patients, especially those with advanced disease. In the context of mCRC, HRQoL endpoint takes on great importance and complements the traditional endpoints in assessment of treatment effectiveness [20]. Consequently, there is a need to propose statistical method for longitudinal analysis of HRQoL that can capture meaningful changes in HRQoL scores.

In this study, we used TUDD, with or without death as an event, as a conservative method that accounts for non-ignorable missing data as the primary endpoint of the HRQoL analysis following the methodology described by Bonnetain et al. [10] for several reasons. Given that a definitive deterioration of HRQoL can be acquired before patients withdraw the study; TUDD is less sensitive to the presence of missing data in the context of advanced disease than classical repeated measurements. Patients with some missing questionnaires are not excluded from the statistical analysis as long as data from at least one HRQoL assessment is obtained. Moreover, if a patient died during follow-up or experienced deterioration, and did not have assessments after that point, this reflected definitive deterioration of the patient’s health. Furthermore, the measure of TUDD is robust and more familiar to clinicians because it is based on Kaplan–Meier survival curves and HR that thus allows them to draw more meaningful estimates of survival.

The TUDD approach is closer to other time-to-event analyses, such as time to progression, and has already been used in the analysis of HRQoL in other cancer locations [9–11,22,23]. In the setting of CRC, Kabbinavar et al. [22] showed that HRQoL was similar whatever the treatment. In that study, the time to deterioration in HRQoL for CRC patient was analyzed as being the time of death or disease progression. We did not include progression of the disease as an event in our analysis. However, when it was used as a time-dependent variable, no association between TTD and progression was found.

The importance of HRQoL dimensions has been reported in many studies since they affect HRQoL in CRC survival. We analysed the HRQoL longitudinal changes in mCRC patients treated with FOLFOX4 versus FOLFOX7-FOLFIRI. Our results show that type of treatment did not significantly influence longitudinal TUDD for the main dimension of the QLQ-C30 scores, suggesting that switching oxaliplatin to irinotecan in the treatment of resectable mCRC does not improve patients’ HRQoL. This result is consistent with those observed in studies of FOLFOX and FOLFIRI in this setting.

The presence of diarrhea and pain hamper the HRQoL among CRC patients with advanced stages [24,25]. In our analysis, patients without symptoms at inclusion presented significantly longer TUDD for diarrhea. Similarly those with dyspnea had significantly longer TUDD for a delay > 12 months between diagnosis of the primary tumor and metastases. The latter observation may be explained by the fact that a longer interval between diagnosis and metastases provides a significant change of recovery to the patient from the first line-treatment effects (after surgery and/or adjuvant chemotherapy) an in turn a satisfactory GHS.

In the present study we found that patients treated with FOLFOX7-FOLFIRI deteriorated less rapidly for pain symptoms scale than those treated with FOLFOX4. However, this result must be interpreted with caution given that the difference between the arms reached statistical significance only in multivariate analysis, possibly due to the effect of unbalanced sample size, an extremely large within group variation, relative to between group variation and the influence of missing data.

Some limitations of the analysis presented here should be noted. Firstly, although the MIROX study represents a large dataset, not all patients completed questionnaires, which could have an impact on the validation of results. Although the non-responders rate was high (39.8%), the patient characteristics were similar to responders. Nevertheless, a 60.2% HRQL response rate is considerable for advance-disease population study. Secondly, as we did not correct for multiple comparisons it may have introduced possible inflation of Type I error and in turn resulted in the lack of significant finding or findings with particularly low significance [26,27].

In conclusion, the present HRQoL results support our recent findings study about the lack of a clinical and statistical significant difference between FOLFOX4 and FOLFOX7-FOLFIRI in the main dimension of the QLQ-C30 scores in CRC patients with advanced disease. The TUDD demonstrates an accessible statistical approach for the longitudinal analysis of HRQoL that in turn are readily meaningful to clinicians and are more likely to influence clinical decision making.

## Supporting Information

S1 Checklist CONSORT Checklist. (DOCX)

S1 Protocol Original version of trial protocol. (DOC)

S2 Protocol English version of trial protocol. (DOC)

S1 Table (DOC)

S2 Table (DOC)

S3 Table (DOCX)

S4 Table (DOCX)

## References

[1] Jemal A, Thomas A, Murray T, Thun M (2002) Cancer statistics. CA Cancer J Clin 52: 23–47. 11814064 10.3322/canjclin.52.1.23

[2] Carsin AE, Sharp L, Cronin-Fenton DP, Céilleachair AO, Comber H (2008) Inequity in colorectal cancer treatment and outcomes: a population-based study. Br J Cancer 99:266–274. 10.1038/sj.bjc.6604467 18594530 PMC2480963

[3] Wei AC, Greig PD, Grant D, Taylor B, Langer B, Gallinger S (2006) Survival after hepatic resection for colorectal metastases: a 10-year experience. Ann Surg Oncol 13:668–676 S. 16523369 10.1245/ASO.2006.05.039

[4] Curley SA (2005) Outcomes after surgical treatment of colorectal cancer liver metastases Semin Oncol 32:S109–111. 10.1053/j.seminoncol.2005.06.011 16399446

[5] Misiakos EP, Karidis NP, Kouraklis G (2011) Current treatment for colorectal liver metastases. World J Gastroenterol 17:4067–4075. 10.3748/wjg.v17.i36.4067 22039320 PMC3203357

[6] Nordlinger B, Sorbye H, Glimelius B, Poston GJ, Schlag PM, Rougier P et al (2008) Perioperative chemotherapy with FOLFOX4 and surgery versus surgery alone for resectable liver metastases from colorectal cancer (EORTC Intergroup trial 40983): a randomised controlled trial. Lancet 371:1007–1016. 10.1016/S0140-6736(08)60455-9 18358928 PMC2277487

[7] Hebbar M, Chibaudel B, Andre T, Mineur L, Smith D, Louvet C et al (2015) FOLFOX4 versus sequential dose-dense FOLFOX7 followed by FOLFIRI in patients with resectable metastatic colorectal cancer (MIROX): a pragmatic approach to chemotherapy timing with perioperative or postoperative chemotherapy from an open-label, randomized phase III trial. Ann Oncol 26:340–347. 10.1093/annonc/mdu539 25403578

[8] Donaldson GW, Moinpour CM (2005) Learning to live with missing quality-of-life data in advanced-stage disease trials.J Clin Oncol 23: 7380–7384. 16186589 10.1200/JCO.2005.07.022

[9] Anota A, Hamidou Z, Paget-Bailly S, Chibaudel B, Bascoul-Mollevi C, Auquier P et al (2013) Time to health-related quality of life score deterioration as a modality of longitudinal analysis for health-related quality of life studies in oncology: do we need RECIST for quality of life to achieve standardization? Qual Life Res 24:5–18. 10.1007/s11136-013-0583-6 24277234 PMC4282717

[10] Bonnetain F, Dahan L, Maillard E, Ychou M, Mitry E, Hammel P et al (2010) Time until definitive quality of life score deterioration as a means of longitudinal analysis for treatment trials in patients with metastatic pancreatic adenocarcinoma. Eur J Cancer 46: 2753–2762. 10.1016/j.ejca.2010.07.023 20724140

[11] Hamidou Z, Dabakuyo TS, Mercier M, Fraisse J, Causeret S, Tixier H et al (2011) Time to deterioration in quality of life score as a modality of longitudinal analysis in patients with breast cancer. Oncologist 16:1458–1468. 10.1634/theoncologist.2011-0085 21948650 PMC3228064

[12] Bonnetain F, Fiteni F, Efficace F, Anota A. Statistical Challenges in the Analysis of Health-Related Quality of Life in Cancer Clinical Trials. J Clin Oncol. 2016 4 18. pii: JCO567974. [Epub ahead of print] 10.1200/JCO.2014.56.7974 27091712

[13] Aaronson NK, Ahmedzai S, Bergman B, Bullinger M, Cull A, Duez NJ et al (1993) The European Organization for Research and Treatment of Cancer QLQ-C30: a quality-of-life instrument for use in international clinical trials in oncology. J Natl Cancer Inst 85:365–376. 8433390 10.1093/jnci/85.5.365

[14] Therasse P, Arbuck S, Eisenhauer E, Wanders J, Kaplan RS, Rubinstein L et al New guidelines to evaluate the response to treatment in solid tumors. European Organization for Research and Treatment of Cancer, National Cancer Institute of the United States, National Cancer Institute of Canada. J Natl Cancer Inst 2000; 92: 205–216. 10655437 10.1093/jnci/92.3.205

[15] Levi F, Misset J, Brienza S, Adam R, Metzger G, Itzakhi M et al A chronopharmacologic phase II clinical trial with 5-fluorouracil, folinic acid, and oxaliplatin using an ambulatory multichannel programmable pump. High antitumor effectiveness against metastatic colorectal cancer. Cancer 1992; 69: 893–900. 1735081 10.1002/1097-0142(19920215)69:4<893::aid-cncr2820690410>3.0.co;2-x

[16] Schemper M, Smith T. A note on quantifying follow-up in studies of failure time. Control Clin Trials 1996; 17: 343–346. 8889347 10.1016/0197-2456(96)00075-x

[17] Kaplan EL, Meier P. Nonparametric estimation from incomplete observations. J Am Stat Assoc 1958; 53: 457–481.

[18] Schoenfeld D. Partial residuals for the proportionnal hazards regression model. Biometrika 1982; 69: 239–241.

[19] Osoba D (1999) Interpreting the meaningfulness of changes in health-related quality of life scores: lessons from studies in adults. Int J Cancer Suppl 12: 132–137. 10679884 10.1002/(sici)1097-0215(1999)83:12+<132::aid-ijc23>3.0.co;2-4

[20] Little RJA (1988) A Test of Missing Completely at Random for Multivariate Data with Missing Values. J Am Stat Assoc 83:1198–1202.

[21] StataCorp. 2009 Stata Statistical Software: Release 11. College Station, TX: StataCorp LP.

[22] Kabbinavar FF, Wallace JF, Holmgren E, Yi J, Cella D, Yost KJ et al (2008) Health-related quality of life impact of bevacizumab when combined with irinotecan, 5-fluorouracil, and leucovorin or 5-fluorouracil and leucovorin for metastatic colorectal cancer. Oncologist 13:1021–1029. 10.1634/theoncologist.2008-0003 18776057

[23] Bezjak A, Tu D, Seymour L, Clark G, Trajkovic A, Zukin M et al (2006) Symptom improvement in lung cancer patients treated with erlotinib: quality of life analysis of the National Cancer Institute of Canada Clinical Trials Group Study BR.21. J Clin Oncol 24:3831–3837. 16921034 10.1200/JCO.2006.05.8073

[24] Ramsey SD, Berry K, Moinpour C, Giedzinska A, Andersen MR (2002) Quality of life in long term survivors of colorectal cancer. Am J Gastroenterol 97(5):1228–1234. 12017152 10.1111/j.1572-0241.2002.05694.x

[25] Gray NM, Hall SJ, Browne S, Macleod U, Mitchell E, Lee AJ et al (2011) Modifiable and fixed factors predicting quality of life in people with colorectal cancer. Br J Cancer 104:1697–1703. 10.1038/bjc.2011.155 21559017 PMC3111166

[26] Godfrey K (1985) Statistics in practice. Comparing the means of several groups. N Engl J Med 313:1450–1456. 4058548 10.1056/NEJM198512053132305

[27] Bauer P (1991) Multiple testing in clinical trials.Stat Med 10:871–890. 1831562 10.1002/sim.4780100609

