# Supplementary material for: Time to Definitive Health-Related Quality of Life Score Deterioration in Patients with Resectable Metastatic Colorectal Cancer Treated with FOLFOX4 versus Sequential Dose-Dense FOLFOX7 followed by FOLFIRI: The MIROX Randomized Phase III Trial
Source: PLoS One. 2016 Jun 16;11(6):e0157067. doi: 10.1371/journal.pone.0157067 (PMC4910973; doi:10.1371/journal.pone.0157067)
Supplement: S1 Protocol — (DOC) [file pone.0157067.s002.doc]

**S1A : Original version of trial protocole**

**ESSAI DE PHASE III DE CHIMIOTHERAPIE PAR FOLFOX 4 OU PAR UNE**

**SUCCESSION FOLFOX 7- FOLFIRI CHEZ DES PATIENTS AYANT DES**

**METASTASES RESECABLES D’ORIGINE COLORECTALE**

**C02-1 - MIROX**

**PROMOTEUR**

**GERCOR**

**22 rue Malher**

**75004 Paris**

**Tél : 01 40 29 85 00**

**Fax : 01 40 29 85 08**

**COORDINATEUR PRINCIPAL**

**Pr. Mohamed Hebbar**

**Unité d’Oncologie Médicale**

**CHRU**

**Hôpital Claude Huriez**

**2 rue Oscar Lambert**

**59037 Lille Cedex**

**Tél : 03 20 44 42 95**

**Fax : 03 20 44 50 23**

**COORDINATEURS ASSOCIES**

**Pr. Luc GAMBIEZ (Lille)**

**Dr. Thierry ANDRE (Paris)**

**Dr. May MABRO (Suresnes)**

**RESUME DE L’ETUDE**

***Titre de l’étude*** Essai de phase III de chimiothérapie par FOLFOX 4 ou par une succession FOLFOX 7 - FOLFIRI chez des malades ayant des métastases résécables d’origine colorectale

**Justification -** Survie 25 à 40 % à 5 ans après exérèse des métastases hépatiques

- Apparent bénéfice pour chimiothérapie intra-artérielle mais technique lourde

- Bas contrôle : FOLFOX 4 (supérieur au LV5FU2 en adjuvant stade III)

- Séquence FOLFOX 7 puis FOLFIRI afin de limiter le risque de résistance tumorale et d’éviter la toxicité neurologique limitante de l’oxaliplatine tout en maintenant une intensité de dose satisfaisante

- Potentialisation possible entre oxaliplatine et CPT11 : induction de lésions de l’ADN par l’oxaliplatine => activation des mécanismes de réparation, dont la topoisomérase I, puis inhibition topoisomérase I par le CPT11

***Objectifs***

**Principal**

Survie sans récidive à 2 ans

**Secondaires**

-Survie globale

-Survie des patients R0, survie des patients R1

-Taux de résections R0, R1, R2

-Taux de réponses objectives (chimiothérapie débutée en pré-opératoire)

- Tolérance

- Qualité de vie

- Taux de complications post-opératoires

- Importance des transfusions lors de l’intervention

- Evaluation des paramètres pharmacogénétiques

***Plan de l’étude*** Essai de phase III randomisé, prospectif, ouvert, multicentrique

***Traitements***

**Bras A** : 12 cycles de FOLFOX 4 :

J1

H0-H2 : acide folinique 200 mg/m2 (si forme dl, 100 si forme l)

H0-H2 : oxaliplatine 85 mg/m²

H2 : 5FU bolus 400 mg/m2 en 15 mn

H2-H24 : 5FU continu 600 mg/m2

J2

H0-H2 : acide folinique 200 mg/m² (si forme dl, 100 si forme l)

H2 : 5FU bolus 400 mg/m2 en 15 mn

H2-H24 : 5FU continu 600 mg/m2

cycles administrés tous les 14 jours

**Bras B** : 6 cycles de FOLFOX 7 puis 6 cycles de FOLFIRI

FOLFOX 7 = LV5FU2 simplifié sans bolus de 5FU + oxaliplatine 130 mg/m2 entre H0 et H2

FOLFIRI = LV5FU2 simplifié + CPT 11 180 mg/m2 entre H0 et H2

cycles administrés tous les 14 jours

La randomisation peut être réalisée :

- après l’exérèse (recommandé)

- avant l’exérèse, en particulier en cas de métastases synchrones. La chimiothérapie serait dans ce cas entreprise avant l’intervention chirurgicale.

Par exemple si bras B : 4 FOLFOX avant l’intervention, 2 FOLFOX et 6 FOLFIRI après.

Les patients R2 seront sortis d’essai

***Population étudiée***

Adénocarcinome colique ou rectal histologiquement prouvé

Métastases hépatiques ou pulmonaires ou péritonéales ou ovariennes isolées (un seul site) résécables

La radiofréquence est acceptée si le nombre de métastases est < 3 et le diamètre maximal des métastases < 35 mm

Indice de performance : OMS < 2

Fonctions hématologique, rénale et hépatique adéquates

Traitements antérieurs :

- Pas de chimiothérapie en situation métastatique

- Chimiothérapie adjuvante (après exérèse de la tumeur primitive) acceptée si protocole FUFOL ou LV5FU2. Les protocoles FOLFOX 4 ou FOLFIRI réalisés en adjuvant sont acceptés à condition qu’il y ait eu un délai > 12 mois entre la fin de la chimiothérapie et la récidive.

Stratifications à l’inclusion :

- Chimiothérapie débutée en pré-opératoire *vs* post-opératoire

- Score de Blumgart 0-1 *vs* 2-3 *vs* 4-5

- Type d’intervention réalisée ou envisagée : chirurgie seule *vs* radiofréquence +/- chirurgie

***Nombre de patients***

142 patients dans chaque bras, soit un total de 284 patients

**SOMMAIRE**

**I. INTRODUCTION________________________________________________________ 7**

**II. PROTOCOLES UTILISES DANS L’ETUDE ________________________________ 8**

II. 1. LV5FU2 ____________________________________________________________ 8

II. 2.FOLFOX (5FU-ACIDE FOLINIQUE-OXALIPLATINE) ______________________ 9

*II.2.1. Essais en monothérapie _____________________________________________ 9*

*II.2.2. Association oxaliplatine-5FU-acide folinique ____________________________ 9*

*II.2.3. Tolérance de l’oxaliplatine __________________________________________ 10*

II.3. FOLFIRI (5FU-ACIDE FOLINIQUE-CPT 11) ______________________________ 11

*II.3.1. CPT 11 en monothérapie ___________________________________________ 11*

*II.3.2. CPT 11 et 5 FU __________________________________________________ 11*

*II.3.3. Tolérance du CPT-11 ______________________________________________ 12*

II.4. SEQUENCES FOLFIRI – FOLFOX ______________________________________ 13

*II.4.1. Etudes comparant un schéma intensifie à une association 5FU-acide folinique _ 13*

*II.4.2. Essai CPT V-308 _________________________________________________ 13*

*II.4.3. Essai FIREFOX __________________________________________________ 13*

**III. RATIONNEL DE L’ETUDE ____________________________________________ 14**

III.1. Faut-il proposer une chimiothérapie après exérèse de métastases hépatiques ? _____ 14

III.2. Quel type de chimiothérapie ? __________________________________________ 14

III.3. Quel type d’étude ? ___________________________________________________ 15

III.4. Schéma FOLFOX 7 puis FOLFIRI : rationnel ______________________________ 16

III.5. Métastases hépatiques synchrones et métachrones __________________________ 16

**IV. ETUDE PHARMACOGENETIQUE______________________________________ 17**

IV.1 Bases Scientifiques ___________________________________________________ 17

*IV.1.1 Polymorphismes pertinents vis-à-vis du 5FU ___________________________ 18*

*IV.1.2 Polymorphismes pertinents vis-à-vis de l'oxaliplatine _____________________ 19*

*IV.1.3 Polymorphismes pertinents vis-à-vis de l'irinotécan ______________________ 20*

IV.2 Considérations pratiques _______________________________________________ 21

*IV.2.1 Réalisation du prélèvement sanguin ___________________________________ 21*

*IV.2.2 Analyse des génotypes d'intérêt ______________________________________ 21*

IV.3 Exploitation des résultats ______________________________________________ 22

IV.4 Aspects éthiques _____________________________________________________ 22

IV.5 Aspects financiers ____________________________________________________ 22

**V. OBJECTIFS DE L’ETUDE ______________________________________________ 22**

V.1. Objectif principal _____________________________________________________ 22

V.2. Objectifs secondaires __________________________________________________ 23

**VI. SCHEMA GENERAL DE L’ESSAI ET SELECTION DES PATIENTS ________ 23**

VI.1. Schéma général de l’essai _____________________________________________ 23

VI.2. Sélection des Patients _________________________________________________ 23

*VI.2.1. Critères d’éligibilité ______________________________________________ 23*

*VI.2.2. Critères d’inéligibilité _____________________________________________ 24*

**VII. DEROULEMENT DE L’ETUDE ________________________________________ 25**

VII.1. Bilan pré et per-opératoire ____________________________________________ 25

VII.2. Type d’intervention : le cas de la radiofréquence ___________________________ 25

VII.3 Nombre de métastases, facteurs pronostiques ______________________________ 25

VII.4. Inclusion des patients ________________________________________________ 26

VII.5. Bilan d’inclusion ____________________________________________________ 27

VII.6. Suivi pendant le traitement ____________________________________________ 28

VII.7. Suivi après le traitement ______________________________________________ 29

VII.8. Durée des traitements ________________________________________________ 29

**VIII. TRAITEMENTS _____________________________________________________ 29**

VIII.1. Bras A : 12 cycles selon le schéma FOLFOX 4 ___________________________ 29

VIII.2. Bras B : FOLFOX 7 puis FOLFIRI _____________________________________ 30

**IX. ADMINISTRATION DES TRAITEMENTS ET ADAPTATIONS POSOLOGIQUES_________________________________________________________31**

IX.1 FOLFOX 4 _________________________________________________________ 31

*IX.1.1. Présentation de l'oxaliplatine _______________________________________ 31*

*IX.1.2. Administration du traitement _______________________________________ 31*

*IX.1.3 . Traitements associés _____________________________________________ 32*

*IX.1.4 Adaptations posologiques __________________________________________ 33*

IX.2. FOLFOX 7 _________________________________________________________ 35

*IX.2.1. Administration du traitement _______________________________________ 35*

*IX.2.2.Traitements associés ______________________________________________ 35*

*IX.2.3. Adaptations posologiques __________________________________________ 36*

IX.3. FOLFIRI ___________________________________________________________ 37

*IX.3.1. Présentation de l'irinotecan __________________________________________ 37*

*IX.3.2. Administration du traitement _________________________________________ 38*

*IX.3.3. Traitements associés ________________________________________________ 38*

*IX.3.4. Adaptations posologiques ____________________________________________ 39*

**X.CRITERES D'EVALUATION ____________________________________________ 39**

X.1. Critère principal ______________________________________________________ 39

X.2. Critères secondaires ___________________________________________________ 39

**XI. EVENEMENTS INDESIRABLES ________________________________________ 40**

XI.1. Définitions _________________________________________________________ 40

XI.2. Conduite à tenir _____________________________________________________ 41

**XII. SORTIE D'ESSAI _____________________________________________________ 41**

XII.1. Causes ____________________________________________________________ 41

XII.2. Surveillance ultérieure _______________________________________________ 41

**XIII. ANALYSE DES DONNEES ____________________________________________ 42**

XIII.1. Nombre de sujets nécessaires _________________________________________ 42

XIII.2. Méthodes statistiques ________________________________________________ 42

XIII.3. Définitions des populations ___________________________________________ 42

XIII.4. Evalution de l’éfficacité ______________________________________________ 43

*XIII.4.1 Paramètres d’efficacité ______________________________________________ 43*

*XIII.4.2. Analyses d’efficacité ________________________________________________ 43*

XIII.5. Evaluation de la tolérance _____________________________________________ 44

XIII.6. Evaluation de la qualité de vie __________________________________________ 44

**XIV. ASPECTS ADMINISTRATIFS, REGLEMENTAIRES ET ETHIQUES _______ 44**

XIV.1. Considérations éthiques ______________________________________________ 44

XIV.2. C.C.P.P.R.B. _______________________________________________________ 45

XIV.3. Monitoring de l’essai _________________________________________________ 45

*XIV.3. 1. Responsabilité des investigateurs ______________________________________ 45*

*XIV.3.2. Responsabilité du coordinateur ________________________________________ 45*

*XIV.3.3. Documents source requis _____________________________________________ 45*

*XIV.3.4. Recueil des données _________________________________________________ 45*

XIV.4. Règles administratives ________________________________________________ 46

*XIV.4.1. Curriculum vitae ___________________________________________________ 46*

*XIV.4.2. Pièces officielles à conserver __________________________________________ 46*

*XIV.4.3. Assurance _________________________________________________________ 47*

*XIV.4.4. Audit inteme et inspection par l'agence du médicament______________________ 47*

XIV.5. Règles de publication _________________________________________________ 47

XIV.6. Modification du protocole______________________________________________ 47

**BIBLIOGRAPHIE ________________________________________________________ 48**

**ANNEXES _______________________________________________________________ 58**

**I. INTRODUCTION**

Les taux de mortalité à 5 ans des adénocarcinomes colorectaux restent importants, de l'ordre de 40 % (1). La survenue de métastases est la principale cause de mortalité. Les métastases hépatiques concernent environ 60 % des patients atteints de cancer colique ou rectal, et le pronostic spontané de ces patients est très mauvais, avec un taux de survie de l’ordre de 1 % à 5 ans. Les métastases sont synchrones dont 1/3 des cas, métachrones dans 2/3 des cas. La résection complète de ces métastases reste le seul traitement validé permettant d'espérer une survie prolongée, voire une guérison. Ce type de traitement n'est toutefois possible que chez 15 à 20 % des patients. Par ailleurs, même quand il est réalisé et que les patients sont rigoureusement sélectionnés, la résection hépatique n'offre que 20 à 40 % de chances de survie à 5 ans (2). Après résection hépatique, près de la moitié des récidives apparaissent dans le foie restant et correspondent au développement de micro-métastases qui étaient présentes initialement, mais n'étaient pas détectables (3). Des métastases extrahépatiques, en particulier péritonéales et pulmonaires surviennent également dans 50 % des cas après résection de lésions hépatiques.

Ceci a incité plusieurs équipes à proposer une chimiothérapie complémentaire intraartérielle hépatique associée ou non à une chimiothérapie systémique.

Certaines études ont tenté dévaluer l’intérêt d’une chimiothérapie intra-artérielle par 5-FU ou FUDR (2, 4-7). Les résultats n’ont pas montré de bénéfice évident pour le taux de récidives intra-hépatique. De plus, une proportion importante des patients développaient des métastases

extra-hépatiques (4). Ceci a conduit certaines équipes à adjoindre une chimiothérapie systémique.

Dans une étude randomisée portant sur 56 patients étaient comparés un traitement combiné chimiothérapie intra-artérielle plus chimiothérapie systémique par 5 FU, à une simple surveillance (8). Les premiers résultats préliminaires plaident en faveur de la chimiothérapie, le taux de survie sans récidive à 3 ans étant de 58 % dans ce bras contre 34 % dans le bras contrôle. Il faut signaler qu’une étude importante similaire n’a pas montré de bénéfice de la

chimiothérapie, la durée médiane de survie étant même (presque significativement) supérieure

dans le bras surveillance (40,8 *versus* 35,4 mois) (6). Une autre étude a suggèré que la chimiothérapie intra-artérielle combinée à la chimiothérapie systémique augmentait la durée de survie sans récidive (9). Dans cette étude, 156 patients ont reçu après randomisation, soit une association chimiothérapie intra-artérielle (FUDR) et systémique (FUFOL), soit la chimiothérapie systémique seule. Le taux de survie à 2 ans était de 86 % dans le groupe traitement combiné, contre 72 % dans l’autre. Les taux de survie sans récidive étaient respectivement de 90 % et de 60 % (p < 10-3). Il faut néanmoins signaler que, dans le bras traitement combiné, seuls 26 % des patients ont reçu plus de la moitié de la dose de FUDR initialement prévue, en particulier du fait de la toxicité hépatique et de problèmes inhérents au cathéter intra-artériel. Ce type de traitement se heurte donc à un problème de faisabilité et doit vraisemblablement être réservé aux centres entraînés. Enfin, dans cette étude, les auteurs signalaient qu’un nombre important de patients étaient résistants au 5-FU (et au FUDR), probablement du fait d’une expression tumorale importante de la thymidylate synthase, ce qui suggère l’intérêt d’agents thérapeutiques agissant sur d’autres cibles (par exemple oxaliplatine et CPT-11).

Seuls deux essais randomisés ont évalué l’intérêt d’une chimiothérapie complémentaire systémique seule. Ces deux essais ont comparé un schéma FUFOL à la surveillance après résection de métastases hépatiques (10, 11). Ces essais indiquaient un bénéfice pour la chimiothérapie tant en termes de survie sans récidive que de survie globale. L’avantage n’était

toutefois pas significatif, ce qui est probablement lié à un manque de puissance statistique. Il faut noter aussi que cet apparent bénéfice est mis en évidence alors que le protocole FUFOL est maintenant dépassé en situation palliative. Dans l’étude présente seront évalués des protocoles plus récents et performants, protocoles d’abord validés en situation métastatique.

**II. PROTOCOLES UTILISES DANS L’ETUDE**

**II. 1. Préalable : LV5FU2**

Ces dernières années, des progrès ont été accomplis dans la chimiothérapie de première ligne en situation métastatique. Celle-ci repose encore souvent sur le 5FU modulé par l’acide folinique, mais des schémas d’administration plus performants ont été élaborés. Ainsi, le protocole LV5FU2, comportant une perfusion continue pendant 48 heures de fortes doses de 5FU est plus efficace et mieux toléré que le classique protocole FUFOL, dans lequel le 5FU était administré à plus faible dose en bolus IV (11). Plus récemment a été évalué le protocole LV5FU2 simplifié comportant de plus fortes doses de 5 FU (2400 à 3000 mg/m2 / 48 heures), mais sans bolus de 5FU et d’acide folinique à J2. En situation métastatique, dans un essai de phase II, le taux de RO était de 40 %, la durée de survie sans progression de 10,2 mois (12). Le schéma LV5FU2 représente la base des protocoles qui seront évalués dans l’étude : FOLFOX 4, FOLFOX 7 et FOLFIRI.

**II. 2. FOLFOX (5FU-ACIDE FOLINIQUE-OXALIPLATINE)**

L’oxaliplatine est un nouveau sel de platine (DACH-platine), dépourvu de toxicité rénale et dont l’activité préclinique est supérieure à celle du cisplatine sur les tumeurs coliques (13). L’oxaliplatine agit au niveau de l’ADN en se fixant préférentiellement aux guanines.

**II.2.1. Essais en monothérapie**

Trois essais multicentriques de phase II ont été réalisés avec l’oxaliplatine seul, regroupant un total de 139 malades atteints de carcinomes colorectaux métastatiques.

- deux essais ont été réalisés selon un schéma comparable : patients antérieurement traités et ayant progressé sous fluoropyrimidines. L’oxaliplatine était administré en perfusion de 2 heures, à la dose de 130 mg / m2 tous les 21 jours (14, 15).

- le troisième essai diffère des deux précédents par le mode d’administration. L’oxaliplatine était administré en perfusion à débit chronomodulé de 5 jours, toutes les 3 semaines : une escalade initiale de doses était prévue, applicable selon la tolérance (150 puis 175 et 200 mg / m2) (16).

Les résultats de ces études sont comparables : taux de réponse de 10 à 11,3 % et médiane de réponse de 8,5 à 9 mois.

**II.2.2. Association oxaliplatine-5FU-acide folinique**

Si l’oxaliplatine a initialement obtenu l’AMM en monothérapie à la dose de 130 mg/m2 tous les 21 jours, il a le plus souvent été utilisé en association au 5FU et à l’acide folinique. Les taux de réponse objectives atteignent alors 30 à 58 % (17-21). En outre, des réponses tumorales sont obtenues même chez des patients qui étaient auparavant réfractaires au 5 FU en première ligne (21). Le GERCOR a réalisé plusieurs essais associant l’oxaliplatine, le 5 FU et l’acide folinique dans des schémas intitulés FOLFOX (23).

Le protocole FOLFOX 4 est représenté par l’adjonction d’oxaliplatine à raison de 85 mg/m2 à un schéma LV5FU2. Le FOLFOX 4 s’est avéré supérieur au LV5FU2 en première ligne métastatique tant en taux de réponses objectives qu’en durée de survie sans progression(24). Le schéma FOLFOX 4 a également été évalué récemment en situation adjuvante après exérèse d’un adénocarcinome colique stade II ou III (étude MOSAIC). Dans cette étude, le FOLFOX 4 était associé à une meilleure durée de survie sans récidive par rapport au schéma LV5FU2 (25). Le protocole FOLFOX 4 est enfin en cours d’évaluation dans un essai randomisé de l’EORTC chez des patients ayant des métastases hépatiques résécables (FOLFOX 4 + chirurgie *versus* chirurgie seule). Les résultats de cet essai devraient être disponibles dans 2 ans, et en cas de positivité, le schéma FOLFOX 4 deviendrait le standard dans cette situation. Tous ces arguments nous conduisent à proposer ce schéma pour le bras de référence dans l’étude présente. Le dernier des schémas FOLFOX (FOLFOX 7) associe le schéma LV5FU2 simplifié à une forte dose d’oxaliplatine à J1 (130 mg / m2). Cette forte dose est justifiée par l’importante relation dose-intensité de l’oxaliplatine pour les taux de réponse et la durée de survie sans progression (26). Dans un essai de phase portant sur 49 patients en deuxième ligne métastatique, le taux de réponse obtenu sous FOLFOX 7 était de 42 %, avec une tolérance neurologique satisfaisante (neuropathie périphérique dans 7 % des cas) (27). Les résultats préliminaires de l’étude OPTIMOX (FOLFOX 7 et LV5FU2 *versus* FOLFOX 4) indiquent une tolérance satisfaisante du schéma FOLFOX 7 (28).

**II.2.3. Tolérance de l’oxaliplatine**

L’oxaliplatine induit, à partir de 45 mg / m2 des nausées et vomissements chez la grande majorité des patients. D’apparition rapide; ils peuvent durer 24 à 48 heures et sont généralement contrôlés par les antagonistes des récepteurs 5 HT3. Des diarrhées transitoires et de faible intensité peuvent être observées. La toxicité limitante de l’oxaliplatine est une neuropathie sensitive périphérique dose totale dépendante, caractérisée par des dysesthésies et/ou des paresthésies distales provoquées ou exacerbées par le froid, généralement régressives entre les cycles ou l’arrêt du produit. L’analyse de 682 malades traités en monothérapie ou association a permis de mieux préciser l’incidence, l’intensité et la réversibilité de ces symptômes. Les échelles de l’OMS ou du NCI n’étant pas bien adaptées à la toxicité neurologique de l’oxaliplatine, une échelle spécifique est utilisée. Des dysesthésies/paresthésies ont été observées chez 82 % des malades. Le seul facteur fortement corrélé avec la durée de la neurotoxicité est la dose totale cumulée de l’oxaliplatine. Le risque de développer des troubles fonctionnels > grade 3 selon l’échelle spécifique) est faible après 3 cycles, de 10 % après 6 cycles et de 50 % après 9 cycles. Après l’arrêt du traitement, l’analyse des patients porteurs d’une symptomatologie neurologique persistante a permis d’observer une régression de ces symptômes chez 82 % des malades sur une période moyenne d’observation de 4 mois et la dispartion complète des manifestations chez 41 % des malades restants après une période d’observation moyenne de 8 mois. En dehors des dysesthésies/paresthésies distales, des manifestations neurologiques aiguës ont été observées : paresthésies laryngopharyngées (63 patients), spasme laryngé ou palpébral (3 patients) et dysphonie et/ou dysphagie (2 patients). Ces manifestations sont généralement d’apparition rapide et disparaissent également rapidement (en quelques minutes généralement). La toxicité hématologique est inconstante. Elle semble être plus fréquente chez les malades ayant une maladie évolutive et/ou lourdement prétraités. Les neutropénies sont rares (< 1,5 % grades 3-4 quand l’oxaliplatine est utilisé seul et < 4 % quand il est associé avec le 5 FU et l’acide folinique). De rares thrombopénies (0,3 % de grade 4) ont été rapportées alors que l’oxaliplatine était utilisé en association avec le 5 FU et l’acide folinique.

**II.3. FOLFIRI (5FU-ACIDE FOLINIQUE-CPT 11)**

Le CPT 11 (irinotécan) est un dérivé semi-synthétique de la camptothécine, inhibiteur sélectif de la topoisomérase I, enzyme essentielle de la réparation de l’ADN. Son métabolite actif est le SN38 (29).

**II.3.1. CPT 11 en monothérapie**

Le CPT 11 a d’abord été administré à la dose de 350 mg / m2 en perfusion de 30 à 90 minutes toutes les trois semaines. Les taux de réponse objective sont identiques en première et en deuxième ligne et sont compris entre 14 et 29 % avec des durées médianes de réponse de 6,9 à

11,5 mois (30-33). En deuxième ligne, une proportion importante de patients (autour de 40 %) ont des stabilisations prolongées. Les principales toxicités sont la neutropénie, la diarrhée qui peut être sévère et l’asthénie.

Deux essais européens de phase III ont démontré un avantage significatif en survie pour les patients traités par CPT-11 après échappement au 5FU, *versus* traitement symptomatique ou 5FU (34, 35)

**II.3.2. CPT 11 et 5 FU**

Le CPT 11 et le 5FU ont des mécanismes d’action différents et n’ont pas de résistance croisée (16). L’association CPT 11 et LV5FU2 a fait l’objet d’un essai de phase I-II chez des patients prétraités par 1 à 5 lignes de chimiothérapie (CPF 106). Cet essai a permis de définir le profil de tolérance et la dose de CPT-11. Les paliers de dose étaient 100 puis 120, 150, 180, 200, 220, 260 et 300 mg/m2. La dose optimale recommandée pour les phases II/III est 180 mg/m2.

Un essai européen de phase III a démontré un avantage significatif pour les patients traités par CPT-11 + 5FU/AF en 1ère ligne versus 5FU/AF, en terme de réponses objectives, de temps jusqu’à progression et de survie globale. (36) Un essai FOLFIRI (CPT-11 + LV5FU2 simplifié) jusqu’à progression puis FOLFOX 6 *versus* séquence inverse a récemment été conduit par le GERCOR en première ligne métastatique (37). L’objectif de cet essai est de déterminer la meilleure séquence en termes de temps jusqu’à progression. Le TTP de la séquence globale est de 14,4 mois pour la séquence Folfiri puis Folfox, versus 11,5 mois pour la séquence inverse (ns). La survie médiane des 2 séquences est supérieure à 20 mois. Les taux de réponses objectives en 1ère ligne sont identiques dans les 2 bras, et supérieurs à 50%

(56% versus 54%). La survie des 2 séquences supérieure à 20 mois conforte l’utilisation des bithérapies en 1ère ligne. Il s’agit en effet de la survie la plus longue obtenue dans une phase III.

**II.3.3. Tolérance du CPT-11**

Tout d’abord dans la phase I toutes les 3 semaines (38), la dose recommandée était de 600 mg/m² (MTD 750 mg/m²), en suivant les recommandations de prise en charge de la diarrhée avec du lopéramide haute dose. Les toxicités limitantes étaient alors hématologique (neutropénie) et digestive (diarrhée). La diarrhée peut être précoce et entrer dans le cadre du syndrome cholinergique fréquemment induit par le CPT11, ou tardive (5ème – 10ème jours) et en rapport avec une toxicité intestinale directe. Cette diarrhée tardive impose le recours aux inhibiteurs de la motilité intestinale, voire à l’hospitalisation pour réhydratation, alimentation parentérale et éventuelle antibiothérapie. Cette toxicité est toutefois exceptionnellement sévère. Par ailleurs, l’alopécie est fréquence après administration de CPR11 à plus de 350 mg / m2. Une étude de phase II a démontré que la dose de 500 mg/m² avait un meilleur ratio efficacité/toxicité que 600 mg/m² (39). L’association du CPT11 à plus faible dose à du 5FU est mieux tolérée. La phase II CPT-11 + LV5FU2 avait atteint un palier de 300 mg/m² de CPT-11. La toxicité limitante était alors une non récupération hématologique à J15 (neutropénie > grade 1) qui ne permettait pas de maintenir le rythme des cures (40).

**II.4. SEQUENCES FOLFIRI – FOLFOX**

**II.4.1. Etudes comparant un schéma intensifie à une association 5FU-acide folinique**

En résumé, les résultats de trois essais randomisés réalisés en première ligne métastatique rapportés récemment :

- deux études ont comparé un schéma associant 5FU-acide folinique-CPT11 à un schéma 5FUacide folinique (36, 41). Dans les deux études, le schéma intensifié a permis d’obtenir davantage de réponses objectives, une meilleure survie sans progression et une meilleure survie globale.

- la troisième étude a comparé un schéma FOLFOX à un schéma LV5FU2 (42). Un meilleur taux de réponse et une plus longue survie sans progression ont été observés dans le bras

FOLFOX. La différence n’était toutefois pas significative pour la survie globale.

Ces trois études confirment la supériorité des schémas intensifiés en termes de réponses

tumorales, de survie sans progression et peut-être en termes de survie globale.

**II.4.2. Essai CPT V-308**

Dans cet essai randomisé du GERCOR, ont été comparées deux séquences thérapeutiques en première ligne chez des patients atteints de carcinome colorectal métastatique : schéma FOLFIRI jusqu’à progression ou toxicité limitante puis schéma FOLFOX *vs* séquence inverse

(37). L’essai a porté sur 226 patients. Les taux de réponses initiales étaient similaires (de l’ordre de 55 %), et aucune différence significative n’a été constatée en termes de survie sans progression et de survie globale. Un profil de tolérance différent, plus d’alopécie, de mucites et de nausée sous FOLFIRI, plus de neutropénie, et présence d’une neurotoxicité sous FOLFOX.

**II.4.3. Essai FIREFOX**

Dans cet essai de phase II coordonné par le GERCOR, a été évalué un schéma alterné 4 cycles de FOLFOX 6 – 4 cycles de FOLFIRI jusqu’à progression ou toxicité limitante (43).

L’objectif était d’une part de réduire le risque de neuropathie liée à l’oxaliplatine en ‘‘intercalant’’ des cycles sans oxaliplatine, d’autre part d’accroître l’efficacité par une synergie entre oxaliplatine et CPT11. En effet, le CPT11, en inhibitant la topoisomérase I, pourrait interférer avec les mécanismes de réparation de l’ADN activés après les lésions liées à l’oxaliplatine. L’étude a porté sur 39 patients atteints de carcinome colorectal en deuxième ligné, après échec d’une association 5FU-acide folinique. La tolérance a été très satisfaisante avec seulement 5,2 % de neuropathies sévères. Par ailleurs, le taux de réponses objectives était de 54,5 %, les durées médianes de survie sans progression et de survie globale étaient respectivement de 10,4 et de 16,8 mois. Cette étude suggère l’intérêt de proposer un schéma combinant FOLFOX et FOLFIRI.

**III. RATIONNEL DE L’ETUDE**

**III.1. Faut-il proposer une chimiothérapie après exérèse de métastases hépatiques ?**

Plusieurs arguments sont en faveur d’une chimiothérapie complémentaire chez des patients ayant des métastases hépatiques résécables d’origine colorectale :

- les récidives seraient liées à la présence de micrométastases présentes dans le foie restant lors de l’intervention chirurgicale. La relation entre taille tumorale et réponse à la chimiothérapie incite à proposer la chimiothérapie le plus précocement possible. L’objectif pouvant être de réduire de nombre de cellules tumorales à moins de 105, seuil en dessous duquel, les mécanismes de défense de l’hôte pourraient en théorie être efficaces.

- la chimiothérapie adjuvante a montré son intérêt après exérèse d’un carcinome colique avec atteinte ganglionnaire, en particulier en diminuant le risque d’apparition de métastases hépatiques à 5 ans, ce qui rejoint l’argument précédent. Récemment, l’étude randomisée

MOSAIC a montré la supériorité du schéma FOLFOX 4 par rapport au LV5FU2 en situation adjuvante après exérèse d’un adénocarcinome colique stade III (André T, et al, N Engl J Med,

2005). Le schéma FOLFOX 4 est donc devenu le standard dans cette situation.

- en situation métastatique, la chimiothérapie palliative de première ligne a montré son intérêt en terme de durée de survie, même à des niveaux de taux de réponse de 15 à 20 %, selon les études Nordic Group et de Scheithauer (44, 45). En outre, dans l’étude du Nordic Group, il est montré que plus le traitement est entrepris tôt (avant l’apparition de symptômes), plus le bénéfice est important pour le patient (44).

- la situation faisant l’objet de cette étude pouvant être considérée comme une situation

intermédiare entre l’adjuvant après exérèse de la tumeur primitive et le métastatique, nous pouvons escompter également un bénéfice d’une chimiothérapie précoce.

**III.2. Quel type de chimiothérapie ?**

- la chimiothérapie intra-artérielle est sans doute une approche rationnelle (répondant aux arguments ci-dessus), mais comme nous l’avons signalé :

- son maniement est délicat, et ses résultats vraisemblablement peu reproductibles dans des centres non entraînés

- les effets indésirables nombreux, limitant la qualité de vie du patient

- elle nécessite d’être optimisée : voies d’abord plus sélectives, chimiothérapies autres que les fluoropyrimidines pour éviter la résistance liée à la surexpression du gène de la thymidylate synthase

- le FUDR n’a pas d’AMM en France, et le 5 FU est beaucoup moins capté par le parenchyme hépatique

- elle ne réduit pas suffisamment le risque de récidives extra-hépatiques, d’où l’intérêt des traitements combinés, ce qui alourdit et complique le traitement.

- la chimiothérapie systémique exclusive mérite d’être évaluée car au cours de ces dernières années ont été élaborés des schémas plus performants en situation métastatique

- deux essais randomisés ont mis en évidence un bénéfice presque significatif du schéma FUFOL par rapport à la surveillance simple après exérèse de métastases hépatiques (10,

11), mais le protocole FUFOL est moins actifs que les schémas plus récents.

- en deuxième puis en première ligne, les schémas intensifiées associant 5 FU, acide folinique et oxaliplatine ou CPTII permettent d’obtenir des taux de réponses tumorales nettement plus élevés qu’auparavant, avec vraisemblablement un gain significatif en termes de durée de survie sans progression pour CPT-11 et oxaliplatine et de survie globale pour CPT-11.

- Les nouvelles molécules que sont l’oxaliplatine et le CPT-11 n’ont pas le même mécanisme d’action que le 5FU. L’oxaliplaine se fixe sur l’ADN pour former des adduits, il est même capable de restaurer la sensibilité au 5 FU chez 50 % des patients réfractaires à cette molécule. Le CPT-11 est un inhibiteur de la topoisomérase I, semblant avoir un effet synergique avec le 5 FU. Les principaux mécanismes de résistance sont également différents : système du glutathion réduit dans un cas, mutation ou hyperexpression de la topoisomérase I dans l’autre.

**III.3. Quel type d’étude ?**

- une étude randomisée comportant comme bras de référence une abstention thérapeutique est difficilement envisageable : plusieurs arguments plaident en faveur de la réalisation d’un traitement complémentaire. De plus, en pratique, de nombreux oncologues proposent déjà un traitement complémentaire. Enfin, proposer ce type d’étude au patient paraît délicat.

- le protocole FOLFOX 4 sera le traitement de référence

- un traitement combiné FOLFOX 7 et FOLFIRI sera également évalué => cette étude est donc un essai de phase III randomisé : chimiothérapie par FOLFOX 4 ou par un schéma combiné FOLFOX 7 puis FOLFIRI.

**III.4. Schéma FOLFOX 7 puis FOLFIRI : rationnel**

Le schéma retenu comporte 6 cures selon le protocole FOLFOX 7 puis 6 cures selon le protocole FOLFIRI. Ce schéma a été retenu pour deux raisons :

- tolérance : le protocole FOLFOX expose à un risque de neuropathie périphérique. Elle est signalée au moins une fois par 80 % des patients, et semble plus fréquente chez les femmes.

Elle peut survenir dès les premiers cycles de chimiothérapie et rapidement devenir invalidante. Cette neurotoxicité est cumulative, limitante, mais généralement réversible si le nombre de cures reste limité. Un relais par un autre traitement non neurotoxique (FOLFIRI) permettrait de dimunuer l’incidence des neuropathies invalidantes.

- efficacité : les mécanismes d’action différents de l’oxaliplatine et du CPTI-11, ainsi que l’absence de résistance croisée plaident en faveur de l’utilisation des deux molécules, ce qui semble corroboré par l’essai FIREFOX (43). En outre, une potentialisation de l’effet du CPT-11 par l’oxaliplatine peut en théorie être attendue. En effet l’action antitumorale du CPT-11 résulte d’une inhibition de la topoisomérase I, enzyme permettant la correction des anomalies topologiques de l’ADN (torsion, surenroulement ...). Cette enzyme intervient plus généralement dans les phénomènes de réparation de l’ADN. Or, une cellule tumorale quiescente, dans laquelle l’ADN est intact, fera moins intervenir la topoisomérase I, et sera donc moins vulnérable au CPT-11. L’oxaliplatine induit des lésions de l’ADN, lésions qui nécessiteront l’activation des mécanismes de réparation, en particulier la topoisomérase I. L’oxaliplatine peut ainsi placer la cellule tumorale dans une situation de vulnérabilité vis-à-vis du CPT 11. Dans une étude réalisée *in vitro*, le CPT 11 ralentissait ainsi la réparation des lésions de l’ADN induites par l’oxaliplatine (46).

- concernant le type de protocole FOLFOX, la relation dose-intensité étant importante pour l’oxaliplatine, nous retenons le schéma FOLFOX 7 comportant 130 mg / m2 d’oxaliplatine. Ce schéma est au moins aussi efficace que le schéma FOLFOX 6 et semble même mieux toléré sur le plan neurologique (24). Afin de réduire le risque de neutropénie et les retards de cures, le bolus de 5FU de J1 a été exclu.

**III.5. Métastases hépatiques synchrones et métachrones**

Le plus souvent, les métastases hépatiques surviennent à distance du traitement de la tumeur primitive. L’exérèse de ces métastases métachrones, si elles sont jugées extirpables, est généralement immédiate. La randomisation sera alors effectuée au décours du geste chirurgical, et le traitement débuté entre 21 et 52 jours après l’intervention.

Chez 15 à 30 % des patients, des métastases hépatiques sont découvertes en même temps que la tumeur primitive. Selon les équipes, deux attitudes peuvent être retenues. Certains préconisent l’exérèse des métastases dans le même temps opératoire que la tumeur primitive. De nombreuses autres équipes préfèrent conserver un intervalle de deux à trois mois entre les deux gestes chirurgicaux. La principale raison est le risque classique d’efflorescence d’autres métastases méconnues après l’ablation de la tumeur primitive. Cette attitude permet donc d’éviter à des patients une intervention hépatique lourde et inutile. Durant cet intervalle, une chimiothérapie est le plus souvent proposée. Les patients entrant dans ce cas de figure peuvent être inclus dans l’étude présente. La randomisation sera alors effectuée après l’exérèse de la tumeur primitive, la chimiothérapie est ensuite débutée puis terminée après l’intervention hépatique pour un nombre total de 12 cures administrées (par exemple 4 FOLFOX 4 avant et 8 FOLFOX 4 après, ou 4 FOLFOX 7 avant, 2 FOLFOX 7 et 6 FOLFIRI après). La reprise de la chimiothérapiue après l’exérèse des métastases doit être effectuée dans un délai de 21 à 52 jours.

**Il convient de rappeler que les métastases doivent être considérées extirpables avant la randomisation (même si elles ne peuvent être réséquées en totalité ensuite). L’objectif n’est pas de rendre accessibles à la chirurgie des localisations initialement jugées ‘‘limites’’.**

Si la chimiothérapie est entreprise après l’exérèse des métastases seuls les patients ayant eu une exérèse R0 seront retenus. Si la chimiothérapie est entreprise avant l’intervention, les patients ayant eu une exérèse R0 ou R1 seront retenus.

**IV. ETUDE PHARMACOGENETIQUE**

**IV.1 Bases Scientifiques**

La variabilité interindividuelle dans la réponse et la toxicité aux anticancéreux peut en partie s'expliquer par un polymorphisme des gènes codant pour les enzymes impliquées dans le métabolisme des médicaments ou codant pour des protéines liées à la pharmacodynamie des médicaments. La pharmacogénétique est la prise en compte de ces polymorphismes. Il est intéressant de souligner que l'analyse des polymorphismes génétiques ouvre la possibilité d'explorer du tissu sain puisque le génotype est constitutionnel.

**IV.1.1 Polymorphismes pertinents vis-à-vis du 5FU**

Polymorphismes de la DPD

La DPD est l'enzyme clef du catabolisme du 5FU, transformant physiologiquement la thymine et l'uracile en dihydrothymine et dihydrouracile, respectivement. La DPD est exprimée dans le foie mais également dans la plupart des tissus, y compris dans les cellules tumorales. Il a été clairement démontré qu'une activité DPD lymphocytaire basse favorisait une surexposition systémique au 5FU associée à un risque élevé de développer une toxicité sévère (50). La fréquence des toxicités sévères au 5FU est de l'ordre de 4 %, et celle des décès iatrogènes de l'ordre de 0.3 %. On estime qu'entre 1 % et 4 % de la population générale est partiellement déficiente en DPD, et que 0.1 % de la population est totalement déficitaire pour cet enzyme. A ce jour, 17 mutations ponctuelles ont été décrites sur le gène de la DPD (51), dont une affectant un site d'épissage sur l'exon 14 (mutation G  A). Cette dernière est associée à une réduction quasitotale de l'activité enzymatique et sa fréquence dans la population générale est estimée entre 1 % et 2 % (52).

Polymorphismes de la MTHFR

Des concentrations intratumorales élevées de 5,10 méthylènetétrahydrofolate sont nécessaires pour que le 5FU inhibe de façon optimale la thymidylate synthase. La méthylènetétrahydrofolate réductase (MTHFR) transforme de façon irréversible le 5,10 méthylènetétrahydrofolate en 5 méthyltétrahydrofolate. Une mutation ponctuelle sur le nucléotide 677 (mutation CT) a été identifiée (53). Dix à 15 % des sujets caucasiens est porteuse du génotype T/T avec une activité enzymatique MTHFR représentant environ 30 % de l'activité des sujets homozygotes C/C. Une seconde mutation, également associée à une baisse d'activité enzymatique, a été démontrée sur le nucléotide 1298 (mutation AC) (54). En théorie, les sujets présentant la mutation devraient être plus sensibles à l'action du 5FU. Une étude récente (55) a en effet suggéré que les patients présentant la mutation en 677 étaient plus sensibles aux fluoropyrimidines que les patients présentant le génotype sauvage.

Polymorphismes de la TS

Une forte expression tumorale de la thymidylate synthase (TS) est associée à une résistance au 5FU (56). Le promoteur du gène de la TS (région 5') est polymorphe, pouvant présenter des répétitions de 28 paires de base, le plus souvent doubles (2R) ou triples (3R). Ces éléments répétitifs influencent l’efficacité de translation du messager de la TS (57). De plus, une mutation ponctuelle GC au niveau de la seconde répétition du génotype 3R a été récemment rapportée, cette mutation influençant également la transcription du gène (58). Des études sur l'influence de ce polymorphisme de répétition vis-à-vis des fluoropyrimidines ont été rapportées (59, 60). Un second polymorphisme associé à une diminution d'expression a été décrit en 3', consistant en la délétion de 6 paires de base en position 1494 (61).

**IV.1.2 Polymorphismes pertinents vis-à-vis de l'oxaliplatine**

Les mécanismes cellulaires responsables de phénomènes de résistance aux dérivés de platine interviennent au niveau cytoplasmique avec les mécanismes de détoxification des radicaux libres via l'enzyme glutathion-transférase  (GST ), et au niveau nucléaire avec les enzymes de réparation des lésions de l'ADN.

Polymorphisme de la GST

Trois isoformes ont été mis en évidence pour la GST : le variant "sauvage" A (Ile105, Ala113), le variant "muté" B (Val105, Ala113) et le variant "muté" C (Val105, Val113). Il a été montré que l'activité lymphocytaire GST était plus basse chez les homozygotes B/B par rapport aux homozygotes A/A (62). Une récente étude conduite sur des cancers colorectaux métastatiques traités par FOLFOX montrait une influence significative du polymorphisme de la GST (codon 105) sur la survie des patients (63).

Polymorphismes des enzymes de réparation du DNA par excision-resynthèse (ERCC)

Le gène ERCC1 présente une mutation AC au nucléotide 8092. Pour XPD (ERCC2), il existe une mutation AC au nucléotide 751 (LysGln). L'importance de ce polymorphisme du gène XPD vis-à-vis de l'efficacité de l'oxaliplatine est été suggérée au plan clinique par Park et collaborateurs (64) qui ont rapporté chez 71 patients avec cancer colorectal avancé traités par FOLFOX que 24 % des patients Lys/Lys répondaient au traitement alors que le taux de réponse n'était que de 10 % chez les sujets Lys/Gln et Gln/Gln (p = 0.015). De plus, la survie était significativement augmentée chez les patients Lys/Lys (64).

**IV.1.3 Polymorphismes pertinents vis-à-vis de l'irinotécan**

Polymorphisme de l'UGT1A1

L'irinotecan (CPT11) est une molécule inactive qui nécessite une activation en SN38, molécule cytotoxique active, éliminée par voie biliaire après glucuroconjugaison par la glucuronyltransférase UGT1A1 (65). L'UGT1A1 présente un polymorphisme de répétition se traduisant par une insertion TA au niveau du promoteur du gène, conduisant soit à l'allèle sauvage (TA)6 ou à sa variante (TA)7, en grande partie responsable du syndrome de Gilbert, caractérisé par un défaut d'élimination de bilirubine. Il a été montré récemment que la glucuroconjugaison du SN38 était moins efficace (baisse de 25 à 50 %) chez les sujets homozygotes 7/7 et hétérozygotes 6/7 en comparaison aux homozygotes sauvages 6/6 (66). La répartition chez les caucasiens donne 9 % de sujets 7/7, 48 % de sujets 6/7 et 43 % de sujets 6/6 (66). La probabilité de rencontrer des sujets déficitaires en UGT1A1, à risque de toxicité par défaut d'élimination du SN38, est de l'ordre de 50 %. Des résultats cliniques préliminaires indiquent que les sujets homozygotes 7/7 développent plus fréquemment des toxicités en cours de traitement par irinotécan (67), suggérant l'intérêt d'une adaptation de dose du CPT11 en fonction du génotype du patient. D'autres mutations ponctuelles en région promoteur du gène UGT 1A1 ont été décrites récemment (68). Il s'agit en particulier des polymorphismes -3279 G > T et -3156 G > A dans l'élément de réponse au phénobarbital qui peuvent contribuer aux anomalies d'hyperbilirubinémie. Au total, la prise en compte des polymorphismes de la DPD, MTHFR et TS pour l'association 5FU-acide folinique, et des polymorphismes de la GST et XPD pour l'Oxaliplatine se justifie dans le cadre d'une étude prospective menée chez des patients avec cancer colorectal avancé traité par l'association 5FU-Acide folinique-Oxaliplatine. La mise en évidence de liens entre ces polymorphismes génétiques constitutionnels et l'efficacité et/ou la toxicité d'un traitement par FOLFOX permettrait d'orienter objectivement le clinicien sur le choix du traitement et pourrait aboutir à du screening individuel à grande échelle sur la base d'une exploration facilement réalisable (ADN constitutionnel).

**IV.2 Considérations pratiques**

**IV.2.1 Réalisation du prélèvement sanguin**

Il s'agira d'effectuer pour chaque patient une prise de sang unique sur tube EDTA de 10 ml (référence GREINER GR10K3). Cette prise de sang peut être effectuée à n'importe quelle heure de la journée, et ne nécessite pas d'être à jeun. Cette prise de sang sera réalisée au bilan précédant le premier cycle de chimiothérapie, cependant le polymorphisme génétique étant constitutionnel (donc identique avant et après traitement), le prélèvement peut exceptionnellement être réalisé à n'importe quel autre moment. Le tube de sang sera directement identifié (au crayon mine noir) sur l'étiquette d'origine du tube (nom + prénom + date), puis directement congelé et stocké à -20°C jusqu'au moment de son expédition au Centre Antoine Lacassagne, à Nice. Le tube ne doit surtout pas être centrifugé. Les tubes seront regroupés tous les 6 à 12 mois (en fonction du recrutement) et seront expédiés à Nice dans de la carboglace (en 24h) par un transporteur agrée pour le transport d'échantillons potentiellement infectieux (acheminement pris en charge par le Laboratoire d'Oncopharmacologie du Centre Antoine Lacassagne). Les fiches de renseignements dûment complétées correspondantes seront jointes aux tubes.

**IV.2.2 Analyse des génotypes d'intérêt**

L'ADN sera extrait au Laboratoire d'Oncopharmacologie sur PAXgeneTM Blood DNA kit (PreAnalytix). A l'exception du génotypage de l'UGT1A1, réalisé au CLCC de Toulouse par le Dr E. Chatelut, les autres polymorphismes seront réalisés au Laboratoire d'Oncopharmacologie du Centre Antoine Lacassagne. Les techniques seront les suivantes :

- Mutation de la DPD sur l'exon 14 : PCR - RLFP

- Génotypes MTHFR en 677 et 1298 : Hybridation différentielle sur LightCycler (Roche)

- Génotypes TS en 5' et 3' : PCR et migration sur gel

- Mutation ponctuelle TS en 5' (GC sur 2ème répétition de l'allèle 3R) : PCR RLFP

- Génotype GST : PCR - RLFP

- Génotype XPD : PCR - RLFP

Version 1.12. février 2008 22

- Génotype UGT1A1(uniquement réalisé chez les patients recevant de l'Irinotécan): génotypage sur séquenceur (E. Chatelut, CLCC de Toulouse).

**IV.3 Exploitation des résultats**

Nous examinerons l'impact de ces polymorphismes constitutionnels sur l'efficacité (réponse clinique) et sur la toxicité (prise en compte du grade le plus fort rencontré au décours de l'ensemble du traitement pour chaque type de toxicité).

L'ensemble de ces analyses statistiques sera réalisé sur le logiciel SPSS par M.C. Etienne- Grimaldi (Centre Antoine Lacassagne).

**IV.4 Aspects éthiques**

Compte tenu que nous analysons une information génétique constitutionnelle, ce projet entre dans le cadre d'application de la loi de Bioéthique. En conséquence, les patients recevront une note d'information spécifique et signeront un consentement éclairé.

**IV.5 Aspects financiers**

Le coût des analyses génétiques, des tubes PAX GeneTM Blood (sur lesquels seront effectués les prélèvements sanguins) et leur acheminement, ainsi que le transport des prélèvements sanguins vers le Laboratoire d'Oncopharmacologie, seront entièrement pris en charge par le Laboratoire d'Oncopharmacologie. Le laboratoire dispose en effet de crédits alloués dans le

cadre d'un programme PHRC national en cours (2003-2005) dédié à la pharmacogénétique.

**V. OBJECTIFS DE L’ETUDE**

**V.1. Objectif principal**

Evaluer l’efficacité d’un schéma de type FOLFOX 4 et d’un schéma combiné par FOLFOX 7 puis FOLFIRI en complément d’une exérèse de métastases d’un carcinome colique ou rectal.

Le critère principal d’évaluation est la survie sans récidive à deux ans.

**V.2. Objectifs secondaires**

- Survie globale

- Survie des patients R0, survie des patients R1

- Tolérance

- Qualité de vie

- Taux de résections R0, R1, R2

- Taux de réponses objectives (chimiothérapie débutée en pré-opératoire)

- Taux de complications postopératoires

- Importance des transfusions lors de l’intervention

- Evaluation des paramètres pharmacogénétiques

**VI. SCHEMA GENERAL DE L’ESSAI ET SELECTION DES PATIENTS**

**VI.1. Schéma général de l’essai**

Il s’agit d’un essai multicentrique, national, prospectif, de phase III, randomisé prévoyant d’inclure 284 patients (142 par bras).

**VI.2. Sélection des Patients**

**VI.2.1. Critères d’éligibilité**

- Adénocarcinome colique ou rectal histologiquement prouvé

- Métastases hépatiques extirpées depuis moins de 52 jours (exérèse complète macroscopiquement et microscopiquement) ou métastases hépatiques considérées extirpables et dont l’exérèse est prévue secondairement

- Absence de métastase extra-hépatique ou de récidive colorectale [NB : Les patients n’ayant que des métastases pulmonaires, ou ovariennes ou péritonéales sont incluables si ces métastases sont réséquées complètement (ou supposées pouvoir l’être)]

- Patients âgés de 18 à 75 ans

- Indice de performance : OMS < 2

- Fonctions hématologique, hépatique et rénale satisfaisantes : PNN > 2.0 109 / l, plaquettes > 100 109 / l, créatinine < 135mmol/l ou clairance  60 ml/mn, SGOT et SGPT < 3 x LSN, phosphatases alcalines < 5 x LSN

- Dosage de l’Antigène Carcino-Embryonnaire (A.C.E.) en pré-opératoire

- Absence de cirrhose

- Absence de complication post opératoire précoce non résolue

- Traitements antérieurs :

 Pas de chimiothérapie en situation métastatique

 Chimiothérapie adjuvante (après exérèse de la tumeur primitive) acceptée si protocole FUFOL ou LV5FU2. Les protocoles FOLFOX 4 ou FOLFIRI réalisés en adjuvant sont acceptés à condition qu’il y ait eu un délai > 12 mois entre la fin de la chimiothérapie et la récidive.

- Consentement écrit signé

**VI.2.2. Critères d’inéligibilité**

Conditions générales

- Patientes enceintes ou en période d’allaitement

- Patientes en âge de procréer et non soumises à une mesure contraceptive adéquate

- Patients ayant déjà reçu une chimiothérapie en situation métastatique

- Résection macroscopiquement incomplète des métastases hépatiques (si l’intervention a eu lieu avant la randomisation). En cas de résection R1, le patient reste inclus dans l’étude.

- Présence de métastases extra-hépatiques ou de récidive colorectale non traitée radicalement

- Autre maladie ou conditions médicales incluant les contre-indications au traitement protocolaire (angine de poitrine non contrôlée ou infarctus du myocarde dans les 6 mois précédents).

- Antécédent d’embolie pulmonaire ou d’accident vasculaire cérébral dans les 6 mois précédents.

- Neuropathie sensitive périphérique avec gêne fonctionnelle

- Pathologie interstitielle préalablement connue

- Antécédents de cancers autres que le cancer colorectal à l’exclusion de cancers reconnus comme guéris (dans ce cas une preuve histologique de la nature colorectale de la métastase est nécessaire) et de tumeurs cutanées basocellulaires et du cancer du col de l’utérus *in situ* traités de façon adéquate et à visée curative

- Patients participant à une autre expérimentation

- Patients qui pour des raisons psychologiques, sociales, familiales ou géographiques ne pourraient pas être suivis régulièrement

**VII. DEROULEMENT DE L’ETUDE**

**VII.1. Bilan pré et per-opératoire**

L’évaluation tumorale comporte un scanner abdominal, de préférence spiralé (ou une IRM), ainsi qu’un scanner thoracique. Elle doit être réalisée dans les 15 jours précédant l’intervention. En cas de métastases synchrones pour lesquelles l’intervention est envisagée secondairement, ces examens morphologiques peuvent être utilisés dans le cadre du bilan d’inclusion. Un nouveau scanner abdominal devra être réalisé en pré-opératoire si le dernier examen date de plus d’un mois. Si la chimiothérapie est entreprise avant l’intervention, un nouveau contrôle tomodensitométrique est également à réaliser en post-opératoire dans les deux semaines précédant la reprise de la chimiothérapie. La réalisation d’une Tomographie par Emission de Positrons est facultative. La réalisation d’une échographie per-opératoire est recommandée. Le résultat histologique de la pièce de résection, confirmant la nature des métastases, leur nombre, le caractère complet ou non de l’exérèse, est indispensable, ainsi que le compte-rendu opératoire.

La première administration du traitement protocolaire doit être planifiée dans les 8 jours suivant la date de la randomisation.

**VII.2. Type d’intervention : le cas de la radiofréquence**

Tout intervention chirurgicale, simple ou complexe, est autorisée, à condition que l’exérèse soit complète (ou supposée devoir l’être si l’inclusion est envisagée avant l’intervention). Un curage ganglionnaire doit être réalisé systématiquement. La radiofréquence est autorisée, seule ou en association à une exérèse chirurgicale, à condition que les métastases traitées par radiofréquence aient un diamètre maximal < 35 mm et que leur nombre n’excède pas 3. Ces métastases doivent être totalement détruites (ou susceptibles de l’être) par la radiofréquence. Une stratification sera réalisée pour ce paramètre à l’inclusion (chirurgie seule *vs* radiofréquence +/- radiofréquence).

**VII.3 Nombre de métastases, facteurs pronostiques**

Il n’y a pas de seuil concernant le nombre de métastases, toujours à condition que ces métastases soient jugées résécables (ou ont été réséquées) complètement. En effet, plusieurs études récentes ont montré que la durée de survie n’était pas influencée significativement par le nombre de métastases réséquées, à condition que l’exérèse soit complète (R0) (47, 48). Toutefois, afin d’éviter un déséquilibre entre les deux bras thérapeutiques, une stratification sera effectuée à l’inclusion en fonction du score de Blumgart (49). Ce score repose sur la détermination de 5 paramètres :

** en cas de non mesure de l’ACE en pré-opératoire, faire un dosage de l’ACE en post-opératoire pour permettre le calcul du score de blumgart*

Pour la stratification, les patients seront répartis en trois catégories : Blumgart 0-1, 2-3 et 4-5.

**VII.4. Inclusion des patients**

Le consentement éclairé doit être signé par le patient dans les 8 jours précédant l’inclusion dans l’essai. Un bilan d’évaluation morphologique doit être pratiqué dans les 15 jours précédant l’inclusion.

Une triple stratification sera réalisée au moment de l’inclusion :

- début de la chimiothérapie pré-opératoire *vs* post-opératoire,

- chirurgie seule vs radiofréquence +/- chirurgie,

- score de Blumgart 0-1 *vs* 2-3 *vs* 4-5

Un bilan biologique et clinique doit être réalisé dans la semaine précédant la date prévue du traitement. Chez les patients inclus après l’intervention chirurgicale, le traitement doit être entrepris entre les 21èmes et 52ème jours suivant l’intervention. Ce délai doit également être respecté pour la reprise de la chimiothérapie chez les patients inclus avant l’intervention. Si seule une radiofréquence percutanée a été réalisée, la chimiothérapie doit être entreprise dans un délai maximal de 28 jours après le geste.

**VII.5. Bilan d’inclusion**

* Les événements indésirables/signes et symptômes préexistants : seront rapportés et gradés selon les critères NCI (ANNEXE IV), en cas d’événements non gradables, selon cette classification, seront gradés comme suit en 1 : léger, 2 : modéré, 3 : sévère, 4 : mettant en jeu le pronostic vital.

**VII.6. Suivi pendant le traitement**

* Les événements indésirables/signes et symptômes préexistants : seront rapportés et gradés selon les critères NCI (ANNEXE IV) et selon l’échelle spécifique adaptée de Lévi pour les neuropathie périphériques (ANNEXE III).En cas d’événements non gradables, selon cette classification, les évènements seront gradés comme suit en 1 : léger, 2 : modéré, 3 : sévère, 4 : mettant en jeu le pronostic vital.

Les investigateurs doivent reporter objectivement tous les événements indésirables dans les 2 groupes même s’ils ne sont pas reliés aux traitements à l’essai (ex : symptômes liés à la maladie)

**VII.7. Suivi après le traitement**

Examen clinique complet, scanner abdominal, radiographie de thorax, taux d’ACE tous les trois mois lors des deux premières années, puis tous les 6 mois durant trois ans.

Coloscopie à un an, puis tous les trois ans.

**VII.8. Durée des traitements**

Les traitements seront poursuivis jusqu'à un total maximal de 12 cycles, ou récidive précoce de la maladie, ou toxicité inacceptable, ou contre-indication à leur poursuite, ou refus du patient.

Remarque : chez les patients inclus dans le bras B (FOLFOX puis FOLFIRI ou séquence inverse), en cas de survenue d’un effet indésirable lié à l’oxaliplatine et contre-indiquant sa réutilisation, le traitement sera poursuivi selon le protocole FOLFIRI jusqu’à un total de 12 cures. En cas de survenue d’un effet indésirable lié au CPT 11 et contre-indiquant sa réutilisation, la chimiothérapie sera interrompue.

**VIII. TRAITEMENTS**

Les patients recevront après randomisation soit 12 cycles de FOLFOX 4 (bras A), soit 6 cycles de FOLFOX 7 suivis de 6 cycles de FOLFIRI (bras B)

**VIII.1. Bras A : 12 cycles selon le schéma FOLFOX 4**

J1 : H0 - H2 : Oxaliplatine 85 mg/m²

H0 – H2 : acide folinique 200 mg/m² en Y (100 mg/m² si forme L)

H2 : 5FU bolus 400 mg / m2 en 15 mn

H2 – H24 : 5FU continu 600 mg/m² en 22 heures

J2 : H0 – H2 : acide folinique 200 mg/m² en Y (100 mg/m² si forme L)

H2 : 5FU bolus 400mg/m² en 15 mn

H2 – H24 : 5FU continu 600 mg/m² en 22 heures

Intervalle entre les cycles : 2 semaines (J1 = J15)

**VIII.2. Bras B : FOLFOX 7 puis FOLFIRI**

***Cycles 1 à 6 : FOLFOX 7***

J1 : LOHP 130 mg / m2 en perfusion continue de 2 heures à partir de H0

J1-J2 : LV5FU2 simplifié

H0 - H2 : acide folinique 400 mg/m² en Y (200 mg/m² si forme L)

H2 - H48 : 5FU continu 2400 mg/m²

Intervalle entre les cycles : 2 semaines (J1 = J15)

***Cycles 7 à 12 : FOLFIRI***

J1 : CPT11 : 180 mg / m2 en perfusion de 30 à 90 mn à partir de H0 en Y.

J1-J2 : LV5FU2 simplifié.

H0 - H2 : acide folinique 400 mg/m² en Y (200 mg/m² si forme L)

H2 : 5FU bolus 400 mg/m² en 15 mn

H2 – H48 : 5FU continu 2400 mg/m²

Intervalle entre les cycles : 2 semaines (J1 = J15)

**IX. ADMINISTRATION DES TRAITEMENTS ET ADAPTATIONS POSOLOGIQUES**

**IX.1 FOLFOX 4**

**IX.1.1. Présentation de l'oxaliplatine**

L'oxaliplatine (ELOXATINE®) est présenté sous forme d'un lyophilisat pour perfusion, en flacons dosés à 50 mg et 100 mg.

La reconstitution s'effectue par addition d'un volume de 10 à 20 ml (pour les flacons à 50 mg) ou de 20 à 40 ml (pour les flacons à 100 mg) d'eau pour préparation injectable ou de solution de glucosé à 5 %, puis dilution dans une solution pour perfusion avec 250 ml ou 500 ml de soluté de glucosé à 5 %.

La reconstitution ou la solution finale ne doit jamais être effectuée avec une solution de chlorure de sodium.

Les flacons d'essai seront étiquetés selon la réglementation en vigueur.

**IX.1.2. Administration du traitement**

toutes les deux semaines

**J1**

H0 - H2 :

acide folinique 200 mg/m² en Y (100 mg/m² si forme L)

Oxaliplatine 85 mg/m² dans 250 mL de G5%

H2: 5FU bolus 400 mg/m² en 10 minutes dans du G5 %

H2 – H24 : 5FU perfusion continue 600 mg/m2 sur 22 heures

**J2**

H0 - H2 : acide folinique 200 mg/m² en Y (100 mg/m² si forme L)

H2: 5FU bolus 400 mg/m² en 10 minutes dans du G5 %

H2 – H24 : 5FU perfusion continue 600 mg/m2 sur 22 heures

**IX.1.3 . Traitements associés**

- L'administration d'un traitement anti-émétique est laissé à l’appréciation de l’investigateur,

- La corticothérapie prescrite comme traitement antiémétique est autorisée.

- Tous les antalgiques et autres traitements symptomatiques sont autorisés.

**IX.1.4 Adaptations posologiques**

La toxicité sera évaluée avant chaque cycle et l'adaptation de doses sera effectuée selon la tolérance au cycle précédant et suivant les modalités suivantes:

En cas d'angor documenté, le patient sortira de l'essai pour toxicité.

En cas de thrombopénie persistante > 75000/mm3 le traitement pourra être poursuivi après avis de l'investigateur Principal.

En cas de toxicité grade 3 ou 4 en intercure, les cycles suivants seront réalisés au palier inférieur, sauf en cas de neutropénie non compliquée < 7 jours ayant récupérée à J15.

**IX.2. FOLFOX 7**

**IX.2.1. Administration du traitement**

toutes les deux semaines

L'oxaliplatine et l'acide folinique ne doivent pas être mélangés mais peuvent être administrés ensemble grâce à une perfusion en Y;

**J1**

H0 - H2 : Oxaliplatine 130 mg/m2 dans 500 ml de G5 %,

H0 - H2 : acide folinique 400 mg/m² en Y (200 mg/m² si forme L) dans 250 ml de G5 %

H2 - H48 : 5FU perfusion continue 2400 mg/m2 sur 46 heures dans du G5 % à 1'abri de la lumière

**IX.2.2.Traitements associés**

- L'administration d'un traitement anti-émétique sera systématique: anti-5HT3 seul ou en association à d'autres classes d'antiémétiques si besoin.

- La corticothérapie prescrite comme traitement antiémétique est autorisée.

- Tous les antalgiques et autres traitements symptomatiques sont autorisés.

**IX.2.3. Adaptations posologiques**

La toxicité sera évaluée avant chaque cycle et l'adaptation de doses sera effectuée selon la tolérance au cycle précédant et suivant les modalités suivantes:

En cas d'angor documenté, le patient sortira de l'essai pour toxicité.

En cas de thrombopénie persistante > 75000/mm3, le traitement pourra être poursuivi après avis de l'investigateur Principal.

La tolérance neurologique sera évaluée selon l'échelle de Lévi (ANNEXE III).

La dose de l'oxaliplatine sera adaptée en fonction de la tolérance clinique neurologique, basée sur la sévérité et la durée des symptômes.

*Le traitement sera alors poursuivi par FOLFIRI jusqu’à un total de 12 cycles.

**IX.3. FOLFIRI**

**IX.3.1. Présentation de l'irinotecan**

Présentation du produit:

Flacons d'une solution prête pour la reconstitution dosés à 40 mg /2 ml ou à 100 mg /5 ml.

Présentation commerciale:

Boite de 1 flacon CAMPTO® avec les mentions légales en vigueur.

**IX.3.2. Administration du traitement**

*FOLFIRI:*

toutes les deux semaines (J1=J15)

Le CPT-11 et l'acide folinique ne doivent pas être mélangés mais peuvent être administrés en Y;

**J1**

H0 - H2 : CPT11 180 mg/m² dans 500 ml de G5 %;

H0 - H2: acide folinique 400 mg/m² en Y (200 mg/m² si forme L) dans 250 ml de G5 %

H2: 5FU bolus 400 mg/m² en 10 minutes dans du G5 %

H2 - H48: 5FU perfusion continue 2400 mg/m² sur 46 heures dans du G5 % à l'abri de la

Lumière

**IX.3.3. Traitements associés**

- L'administration d'un traitement anti-émétique est laissée à l’appréciation de l’investigateur

- La corticothérapie prescrite comme traitement antiémétique est autorisée.

- Tous les antalgiques et autres traitements symptomatiques sont autorisés.

**IX.3.4. Adaptations posologiques**

La toxicité sera évaluée avant chaque cycle et l'adaptation de doses sera effectuée selon la tolérance au cycle précèdent et suivant les modalités suivantes:

-En cas d'angor documenté, le patient sortira de l'essai pour toxicité. Récupération = PNN > 1500/mm, plaquettes > 100 000/mm3

- En cas de thrombopénie persistante > 75000/mm3, le traitement pourra être poursuivi après avis de l'investigateur Principal.

- En cas de toxicité grade 3 ou 4 en intercure, les cycles suivants seront réalisés au palier inférieur, sauf en cas de neutropénie non compliquée < 7 jours ayant récupérée à J15.

- En cas de persistance de la diarrhée tardive d'un grade > 2 ou d'une neutropénie sévère (grade 4 > 7 jours ou neutropénie fébrile) malgré une diminution du CPT-11 à 150 mg/m², le traitement sera interrompu et le patient sorti de l’étude.

**X.CRITERES D'EVALUATION**

**X.1. Critère principal**

Le principal critère d’évaluation est le taux de survie sans récidive à deux ans.

**X.2. Critères secondaires**

- Taux de Survie sans récidive et taux de survie globale

- Incidence des toxicités selon les grades NCI-CTC ou échelle spécifique

- Analyse des questionnaires qualité de vie EORTC-QLQC30

- Taux de résections R0, R1, R2, survie des patients R0, survie des patients R1

- Taux de réponses objectives (chimio débutée en pré-opératoire)

- Taux de complications postopératoires

- Importance des transfusions lors de l’intervention

*Les toxicités biologiques, cliniques ou symptomatiques seront gradées selon les critères NCI-CTC. Les effets indésirables qui ne sont pas reportés dans cette classification seront gradés comme suit: mineur, modéré, sévère ou mettant en jeu le pronostic vital.

**XI. EVENEMENTS INDESIRABLES**

**XI.1. Définitions**

Les événements indésirables devront être recueillis durant 30 jours suivant la dernière administration de la chimiothérapie.

Un événement indésirable est considéré comme grave qu’il soit imputable à la recherche ou non si l'une des conditions suivantes au moins est remplie:

- il entraîne le décès ou met en jeu le pronostic vital du patient

- il nécessite ou prolonge une hospitalisation

- il entraîne une invalidité ou une incapacité

- il entraîne un cancer secondaire, ou une anomalie congénitale

• Enfin, d'autres évènements ne répondant pas aux qualifications ci-dessus énumérés, pourront être considérés comme potentiellement graves par le médecin prescripteur et le promoteur. Il peut s'agir en particulier des "évènements indésirables inattendus".

• Un événement est susceptible "d'être dû à la recherche" si sa survenue ne peut raisonnablement être attribuée à une cause indépendante des conditions de la recherche. En cas de désaccord entre l'investigateur et le promoteur sur l'existence d'un lien entre la recherche et l'évènement, ce dernier sera toujours considéré comme "susceptible d'être dû à la recherche".

**XI.2. Conduite à tenir**

Tout événement indésirable grave ou potentiellement grave doit être rapporté au promoteur dans les 24 heures ouvrées :

- par fax = 01.40.29.85.08

- par téléphone = 01.40.29.85.00

Le clinicien rédigera un rapport écrit dans les 24 heures suivant la découverte d'un événement indésirable grave à l'aide du formulaire de déclaration (annexe VII). Celui-ci sera envoyé à l'adresse ci-dessous:

GERCOR

22, rue Malher

75 004 PARIS

Le promoteur déclarera à l’AFSSAPS tout événement indésirable grave susceptible d'être dû à la recherche.

**XII. SORTIE D'ESSAI**

**XII.1. Causes**

Tous les détails concernant les raisons de la sortie de l'essai et les informations sur le suivi seront notés dans le cahier d'observation.

Le traitement peut être interrompu et le patient sorti d’essai à tout moment pour les raisons suivantes :

- progression tumorale

- apparition d'une toxicité menaçante pour la vie du patient et ceci en dépit de l'adaptation des doses

- refus du patient ou décision d'arrêt prise par l'investigateur dans l'intérêt du patient.

**XII.2. Surveillance ultérieure**

Les patients sortis pour toxicité seront suivis attentivement jusqu'à résolution de celle-ci.

Dans tous les cas, la surveillance s'imposera jusqu'à la date du décès.

**XIII. ANALYSE DES DONNEES**

**XIII.1. Nombre de sujets nécessaires**

Considérant que le taux de survie sans récidive à deux ans dans le bras contrôle (FOLFOX 4) est de 25% et qu’avec un traitement sous FOLFOX puis FOLFIRI, il passerait à 35%, il est nécessaire d’observer *188* événements (*94* événements par groupe), selon un test du logrank bilatéral, un risque α égal à 5% et une puissance de 80%.

Une période de recrutement uniforme de 36 mois et un suivi minimum de 24 mois sont anticipés.

En faisant l’hypothèse que la survie sans récidive suit une distribution exponentielle, 124 patients par bras seront nécessaires. En supposant un taux de patients perdus de vue et de patients présentant une morbidité ne permettant pas une reprise de la chimiothérapie égal à 20%, 142 patients devront être inclus dans chaque bras de traitement. La date de cut-off correspondra à la date où le 188ème événement sera observé.

**XIII.2. Méthodes statistiques**

L’analyse statistique sera réalisée à l’aide du logiciel SAS version 6.12 ou ultérieure pour Windows. Tous les tests statistiques seront bilatéraux à un niveau de significativité de 5%. Un plan d’analyse statistique sera rédigé avant le gel de la base de données. Les variables quantitatives seront décrites à l’aide de la médiane, moyenne, écart type de la moyenne, minimum et maximum. Les variables qualitatives seront décrites par leur fréquence et leur pourcentage.

Pour les proportions l’intervalle de confiance à 95% sera calculé à l’aide de la méthode exacte.

Afin de comparer les variables qualitatives, entre les deux bras de traitement, un test du χ² ou un test de Fisher sera utilisé. Les variables quantitatives seront comparées à l’aide d’un test de

student.

Les courbes de survie seront estimées à l’aide de la méthode de Kaplan-Meier. Un test du Logrank sera utilisé pour comparer les deux bras de traitement.

Une analyse multivariée sera réalisée afin de prendre en compte les facteurs pronostics. Cette analyse multivariée sera réalisée à l’aide d’un modèle de Cox.

**XIII.3. Définitions des populations**

Les populations suivantes seront considérées :

- population dite en ITT « modifiée » : il s’agit de l’ensemble des patients randomisés ayant reçu au moins une dose de chimiothérapie, opérés et non R2 à l’issue de la chirurgie. Ces patients seront analysés dans le bras assigné par la randomisation.

- population des patients éligibles : cette population est constituée des patients ne présentant aucune déviation majeure aux critères d’inclusion et de non inclusion.

- population évaluable pour l’analyse de la tolérance : il s’agit de l’ensemble des patients randomisés ayant reçu au moins une dose de chimiothérapie. Les patients seront analysés dans le groupe de traitement effectivement reçu.

**XIII.4. Evalution de l’éfficacité**

**XIII.4.1 Paramètres d’efficacité**

*XIII.4.1.1. Critère principal d’efficacité*

Le critère principal d’efficacité est la survie sans récidive analysée sur la population en ITT modifiée.

*XIII.4.1.2. Critères secondaires d’efficacité*

Les critères d’efficacité secondaires sont le taux de survie sans récidive à 5 ans, la survie globale, le taux de complications post-opératoires, l’importance des transfusions lors de l’intervention.

Le taux de R0, R1 et R2 à l’issue de la chirurgie et la survie des patients R0/R1 seront décrits.

L’ensemble des patients R2 à l’issue de la chirurgie seront décrits à part.

**XIII.4.2. Analyses d’efficacité**

*XIII.4.2.1. Analyse principale*

L’objectif principal de cette étude est de détecter une augmentation statistiquement significative de la survie sans récidive dans le bras expérimental par rapport au bras contrôle. Un test du logrank non ajusté sera utilisé pour comparer les deux bras de traitement.

*XIII.4.2.2. Analyses secondaires*

Les analyses secondaires seront réalisées sur la population en ITT modifiée.

Une analyse multivariée, à l’aide du modèle de Cox, sera réalisée pour la survie sans récidive.

Les facteurs pronostics seront précisés dans le plan d’analyse statistique.

**XIII.5. Evaluation de la tolérance**

L’analyse de la tolérance sera réalisée sur la population évaluable pour la tolérance.

Les analyses concerneront la toxicité hématologique (neutropénie, thrombocytopénie, anémie), les neutropénies fébriles, les infections, les autres toxicités non hématologiques et la toxicité biochimique.

La version 2 de la classification NCI-CTC sera utilisée pour grader les toxicités. Les analyses présenteront le grade maximum ou la sévérité maximum par patient et par cycle. Seront considérées dans ces analyses d’une part toutes les toxicités d’autre part les toxicités reliées au traitement.

Le nombre de patients présentant au moins un grade 3-4 au cours de l’essai sera comparé entre les deux bras de traitement.

Des analyses seront effectuées en fonction des bilans biologiques (hématologies et biochimies) par patient et par cycle. Les paramètres biochimiques analysées seront : la créatinine, les phosphatases alcalines, SGOT/SGPT et la bilirubine totale.

Les événements intercurrents graves seront analysés par patients. Le nombre d’événements intercurrents graves sera présenté pour chaque groupe en fonction de leur relation au traitement.

**XIII.6. Evaluation de la qualité de vie**

La qualité de vie sera évaluée à l’aide du questionnaire EORTC-QLQC30

**XIV. ASPECTS ADMINISTRATIFS, REGLEMENTAIRES ET ETHIQUES**

**XIV.1. Considérations éthiques**

Les investigateurs s'engagent à conduire cet essai selon les conditions de la loi Huriet du 20 décembre 1988, modifiée en juillet 1994, et en accord avec le déclaration d'Helsinki telle qu'elle a été révisée à Tokyo et à Venise.

lls s'engagent à recueillir par écrit le consentement éclairé du patient avant toute inclusion (annexe VI).

Auparavant, le patient sera informé de la nature de l'essai, du traitement envisagé avec ses bénéfices et effets secondaires éventuels (lettre d'information en annexe V).

**XIV.2. C.C.P.P.R.B.**

**XIV.3. Monitoring de l’essai**

**XIV.3. 1. Responsabilité des investigateurs**

L'investigateur s'engage à conduire l'essai selon les Bonnes Pratiques Cliniques.

Les patients seront recrutés conformément aux critères d'inclusion et de non inclusion;

I'administration et le suivi se feront selon les recommandations protocolaires.

Un recrutement de 220 patients est prévu. La durée des inclusions sera de 36 mois.

**XIV.3.2. Responsabilité du coordinateur**

L'essai sera conduit en accord avec les recommandations ministérielles concernant les

Bonnes Pratiques Cliniques (BPC). Un suivi régulier sera effectué par le coordinateur qui informera tous les investigateurs des événements indésirables graves de l'essai et des amendements éventuels nécessaires.

**XIV.3.3. Documents source requis**

Les documents originaux nécessaires au suivi de l'essai et à la vérification des données du cahier d'observation de l'essai sont :

- le cahier d'observation clinique du patient, le cahier de soins infirmiers,

- les examens biologiques permettant l'évaluation de la toxicité du traitement et d'une éventuelle efficacité (marqueur sérique)

- les examens radiologiques permettant l'évaluation tumorale.

**XIV.3.4. Recueil des données**

Il se fera sur un cahier d'observation rempli pour chaque malade qui comprend :

- Consentement écrit (annexe VI)

- Identification (N° d'inclusion sur chaque page et trois premières lettres du nom du patient).

- Fiche du bilan avant inclusion.

- Fiche de traitement chimiothérapique (une fiche à remplir pour chaque cycle de traitement: examen clinique, bilan biologique et cotation des toxicités qui seront transcrits sur le cahier).

- Fiche d'évaluation de la réponse tumorale.

- Fiche de sortie d'essai.

- Fiche de déclaration des évènements indésirables graves.

- Fiche de surveillance.

Ce cahier d'observation est un cahier simple.

Une photocopie sera faite et transmise à l'organisme qui sera en charge de la saisie et de l'analyse des données.

Un stylo à encre noire sera utilisé pour inscrire les données afin de garantir la bonne qualité des photocopies du cahier d'observation.

Seuls les investigateurs et les co-investigateurs officiellement déclarés pourront inscrire les données sur le cahier d'observation. En cas d'erreur, les corrections seront inscrites en rayant les données incorrectes (qui doivent rester lisibles) et seront datées et paraphées par l'investigateur.

L'investigateur devra revoir l'exactitude, la véracité et l'état complet des données du cahier d'observation et signer chaque feuillet.

**XIV.4. Règles administratives**

**XIV.4.1. Curriculum vitae**

Les investigateurs et co-investigateurs participant à cet essai sont tenus de fournir au promoteur leur *curriculum vitae* avec titres et travaux.

**XIV.4.2. Pièces officielles à conserver**

L'investigateur doit conserver aussi longtemps que le médicament est autorisé, les documents suivants :

- les documents source c'est à dire les dossiers individuels des patients entrés dans l'essai

- les cahiers d'observation de l'essai

- le protocole d'essai

- le rapport de l'essai

- toutes pièces en rapport avec l'essai (correspondance, gestion du stock, ...)

La liste des codes d'identification des patients doit être gardée 15 ans.

**XIV.4.3. Assurance**

Le promoteur a souscrit une assurance garantissant sa responsabilité civile et celle de chaque investigateur. Le promoteur assume l'indemnisation des conséquences dommageables de la recherche pour la personne qui s'y prête, dues à l'application exacte du protocole, sans que puisse être opposé le fait d'un tiers ou le retrait volontaire de la personne qui avait initialement consenti à se prêter à la recherche.

Numéro d'assurance : 2002064

Une attestation nominative est remise à l'investigateur, lors de la visite de mise en place.

Un spécimen mentionnant le titre et le code de l'étude est annexé à ce protocole (annexe X).

**XIV.4.4. Audit inteme et inspection par l'agence du médicament**

Dans le but d'obtenir des résultats les plus homogènes possibles, des audits pourront avoir lieu en cours d'essai. Par ailleurs, les données de l'essai doivent pouvoir être consultées en cas d'inspection par l’AFSSAPS.

**XIV.5. Règles de publication**

Toute ou une partie de l'essai pourra faire l'objet d'une publication scientifique sous la responsabilité des investigateurs principaux selon le règlement du GERCOR. Les coinvestigateurs ayant inclus au moins 5 % des patients seront co-auteurs d'une publication scientifique.

**XIV.6. Modification du protocole**

Toute modification du protocole affectant les objectifs de l'essai, la conception de l'essai, la population des patients, les procédures de l'essai ou des aspects administratifs significatifs nécessiteront un amendement formel du protocole. Avant sa mise en oeuvre, le promoteur, le coordinateur et le C.C.P.P.R.B. donneront leur accord à un tel amendement, qui sera porté à la connaissance de tous les investigateurs de l'essai.

Les modifications administratives du protocole sont des corrections ou éclaircissements secondaires qui n'affectent pas la manière dont l'essai doit être mené. Le promoteur et l'investigateur donneront leur accord à ces changements administratifs et ils seront mis par écrit. Le C.C.P.P.R.B. doit être averti de ces changements administratifs.

**BIBLIOGRAPHIE**

1. American Joint Committee on Cancer - Manual for staging of cancer. Philadelphia: JB Lippincott Company, 1192 - 4• edition.

2. Nordlinger B, Guiguet M, Vaillant JC, et al. Surgical rsection of colorectal carcinoma metastases to the liver. Cancer 1996, 77: 254-62

3. Panis Y, Ribeiro J, Chretien Y. Dormant liver metastases : an experimental study. Br J Surg

1992, 79: 221-23

4. Gambiez L, Denimal F, Karoui M, Dewailly V, Pruvot FR, Quandalle P. Chimiothérapie intra-artérielle adjuvante après résection curative de métastases hépatiques d’un cancer colorectal. Résultats d’une étude pilote chez 30 patients. Chirurgie 1999, 124: 640-8

5. Porte H, Demoulins H, Gambiez L, Wurtz A, Quandalle P. A pilot study of adjuvant hepatic arterial infusion chemotherapy, associating 5-fluorouracil and leucovorin, after resection of colorectal cancer liver metastases. Surg Oncol 1995, 4: 417-22

6. Lorenz M, Muller HH, Schramm H, et al. Randomized trial of surgery followed by adjuvant hepatic arterial infusion with 5-fluorouracil and folinic acid for liver metastases of colorectal cancer. Ann Surg 1998, 228: 756-62

7. Curley SA, Roth MS, Chase JL, Hohn DC. Adjuvant hepatic arterial infusion chemotherapy after curative resection of colorectal liver metastases. Am J Surg 1993, 166: 743-8

8. Kemeny MM, Adak S, Lipsitz S, et al. Results of the Intergroup (Eastern Cooperative

Oncology (ECOG) and Southwest Oncology Group (SWOG)) prospective randomized study of surgery alone versus continuous hepatic artery infusion of FUDR and continuous systemic

infusion after hepatic resection for colorectal liver metastases. Proc Am Soc Clin Oncol 1999,

18: 264a

9. Kemeny N, Huang Y, Cohen AM, et al. Hepatic arterial infusion of chemotherapy after

rescetion of hepatic metastases from colorectal cancer. N Engl J Med 1999, 341: 2039-48

10. Portier G, Rougier P, Milan C, Bouché O, Gillet M, Bosset JF, Ducreux M, Saric J, Bugat R, Stremsdoerfer N, Nordlinger B, Bedenne L, Lazorthes F. Adjuvant systemic shemotherapy using 5-fluorouracil and folinic acid after resection of liver metastases from colorectal origin. Results of an intergroup phase III study. Proc Am Soc Clin Oncol 2002 (Abs 528)

11. Langer B, Bleiberg H, Labianca R, Shepherd L, Nitti D, Marsoni S, Tu D, Sargeant AM,

Fields A. Fluorouracil plus L-leucovorin versus observation after potentially curative resection of liver or lung metastases from colorectal cancer : results of the ENG randomized trial. . Proc Am Soc Clin Oncol 2002 (Abs 592)

11. de Gramont A, Bosset JF, Milan C, et al. A prospectively randomized trial comparing 5FU bolus with low dose folinic acif (FUFOLUD) and 5FU bolus plus continuous infusion with high dose folinic acid (LV5FU2) for advanced colorectal cancer. A French intergroups study. J Clin Oncol 1997; 15: 808-15

12. Tournigand, C, de Gramont D, Louvet C, et al. A simplified bimonthly regimen with leucovorin and 5-fluorouracil for metastatic colorectal cancer. Proc Am Soc Clin Oncol 1998,

17: 274a

13. Pendyala L, Creaven PJ, Shah G. et al. In vitro cytotoxic studies of oxaliplatin in human tumor cells lines. Proc Am Assoc Cancer Res 1991, 32: 410

14. Machover D, Misset JL, de Gramont A et al. Essai de phase II d'évaluation de l'efficacité

antitumorale de l'oxaliplatine par monothérapie chez des patients atteints d'adénocarcinome colorectal avancé résistants aux fluoropyrimidines. Rapport interne DEBIOPHARM, Septembre 1994.

15. Diaz-Rubio E, Marty M, Extra JM et al. Multicentric phase II study with oxaliplatin (L-OHP) in 5 FU refractory patients with advanced colorectal cancer. Fifht International Congress on Anticancer Chemotherapy, Paris, February 1995, Abstract 0721 : 161.

16. Levi F, Perpoint B, Garufi C et al. Oxaliplatin activity against metastatic colorectal cancer: A phase II study of 5-day continuous venous infusion at circadian-rythm modulated rate. Eur J Cancer, 1993; 29A, 1284 - 1293.

17. Levi F, Misset JL, Brieuza S et al. Essai de phase II de l'oxaliplatine associé au 5-FU et à l'acide folinique dans le traitement du cancer colorectal avancé. Rapport interne, janvier 1994.

18. Levi F, Misset JL, Brienza S et al. A Chronopharmacologic Phase II Clinical Trial with

5-Fluorouracil, Folinic Acid, and Oxaliplatin Using an Ambulatory Multichannel Programmable Pump. Cancer 1992; 69: 893-900.

19. Levi F, Misset JL, Vannetzel JM et al. Chronotherapy with 5-Fluorouracil, Folinic Acid and Oxaliplatin in Patients with Previously Untreated Metastatic Colorectal Cancer. Rapport interne, juin 1993.

20. Levi F, Zidani R, Vannetzel JM et al. Chronomodulated Versus Fixed Infusion Rate Delivery of Ambulatory Chemotherapy with Oxaliplatin, Fluorouracil, and Folinic Acid (Leucovorin) in Patients with Colorectal Cancer Metastases: A Randomized Multi-Institutional Trial. J Nat Cancer Inst 1994; 86: 1608-17

21. Levi F, Misset JL, Vannetrel JM, et al. Chronothérapie par 5-Fluorouracile, acide folinique et oxaliplatine chez des patients atteints de cancer colorectal non prétraité. Rapport interne, octobre 1994

22. Garufi C, Brienza S, Bensmaine MA, et al. Addition of Oxaliplatin (L-OHP) to

chronomodulated (CM) 5-Fluorouracil (5-FU) and Folinic Acid (FA) for reversal of acquired

chermoresistance in patients with advanced colorectal cancer (ACC). Proc Amer Soc Clin Oncol 1995, 14: 446

23. de Gramont A, Tournigand T, Louvet C et al. Oxaliplatin with high-dose folinic acid and 5-fluorouracil 48-hour infusion in pretreated metastatic colorectal cancer. Europ J Cancer 1997,33: 214-9

24. de Gramont A, Figer A, Seymour M, et al. Leucovorin and fluorouracil with or without oxaliplatin as first-line treatment in advanced colorectal cancer. J Clin Oncol 2000, 18: 2938 47

25. de Gramont A, et al. Oxaliplatin/5FU-LV in adjuvant colon cancer: results of the international randomized mosaïc trial. Proc Am Soc Clin Oncol 2003, 22: 253 (Abs 1015)

26. de Gramont A, Maindrault-Goebel F, Louvet C, et al. Evaluation of oxaliplatin dose-intensity with the bimonthly 48h leucovorin and 5-fluorouracil regimens (FOLFOX) in pretreated metastatic colorectal cancer. Proc Am Soc Clin Oncol 1999

27. Maindrault-Goebel F, de Gramont A, Louvet C, et al. High-dose oxaliplatin with the simplified 48h bimonthly leucovorin and 5-fluorouracil regimen (FOLFOX 7) in pretreated metastatic colorectal cancer. Proc Am Soc Clin Oncol 1999

28. André T, et al. FOLFOX7 compared to FOLFOX4 ; Preliminary results of the randomized optimox study. Proc Am Soc Clin Oncol 2003, 22: 253 (Abs 1016).

29. Armand JP. Irinotecan (CPT11): recent clinical development and future direction. Ann

Oncol, 5(Suppl 5):A360 1994.

30. Rothenberg ML, Eckardt JR, Burris HA 3rd, et al. Irinotecan (CPT-11) as second-line therapy for patients with 5-FU-refractory colorectal cancer. Proc Am Soc Clin Oncol 1994,

13:A578

31. Pitot HC, Wender D, O'Connell MJ, Wieand HS, Mailliard JA. Phase II trial of CPT-11 (irinotecan) in patients with metastatic colorectal carcinoma: a North Central Cancer Treatment Group (NCCTG) study. Proc Am Soc Clin Oncol 1994, 13:A573

32. Bugat R, Suc E, Rougier P, et al. CPT-11 (irinotecan) as second-line therapy in advanced colorectal cancer (CRC): preliminary results of multicentric Phase II study. Proc Am Soc Clin

Oncol 1994, 13:A586

33. Rougier P, Bugat R, et al. Phase II study of irinotecan in the treatment of advanced colorectal cancer in chemotehrapy naïve patients and patients pretreated with fluoro-uracile based chemotherapy. J Clin Oncol 1997, Vol. 15, 251-60

34. Cunningham D, Pyrhonen S, James RD, Punt CJ, Hickish TF, Heikkila R, Johannesen TB,

Starkhammar H, Topham CA, Awad L, Jacques C, Herait P. Randomised trial of irinotecan plus supportive care versus supportive care alone after fluorouracil failure for patients with metastatic colorectal cancer. : Lancet 1998, 352: 1413-8.

35. Rougier P, Van Cutsem E, Bajetta E, Niederle N, Possinger K, Labianca R, Navarro M,

Morant R, Bleiberg H, Wils J, Awad L, Herait P, Jacques C. Randomised trial of irinotecan versus fluorouracil by continuous infusion after fluorouracil failure in patients with metastatic colorectal cancer. Lancet 1998, 352: 1407-12.

36. Douillard JY, Cunningham D, Roth AD, et al. Irinotecan combined with fluorouracil compared with fluorouracil alone as first-line treatment for metastatic colorectal cancer: a multicentre randomised trial. Lancet 2000, 355: 1041-7

37. Tournigand C, Andre T, Achille E, Lledo G, Flesh M, Mery-Mignard D, Quinaux E, Couteau C, Buyse M, Ganem G, Landi B, Colin P, Louvet C, de Gramont A. FOLFIRI followed by FOLFOX6 or the reverse sequence in advanced colorectal cancer: a randomized GERCOR study. J Clin Oncol 2004, 22: 229-37.

38. Abiberges D, Chabot GG, Armand JP, Herait P, Gouyette A, Gandia D. Phase I and pharmacologic studies of the camptothecin analog irinotecan administered every 3 weeks in cancer patients. J Clin Oncol 1995, 13: 210-21.

39. Merrouche Y, Extra JM, Abigerges D, Bugat R, Catimel G, Suc E, Marty M, Herait P,

Mahjoubi M, Armand JP. High dose-intensity of irinotecan administered every 3 weeks in advanced cancer patients: a feasibility study. J Clin Oncol 1997, 15: 1080-6.

40. Ducreux M, Ychou M, Seitz JF, Bonnay M, Bexon A, Armand JP, Mahjoubi M, Mery-

Mignard D, Rougier P. Irinotecan combined with bolus fluorouracil, continuous infusion fluorouracil, and high-dose leucovorin every two weeks (LV5FU2 regimen): a clinical dosefinding and pharmacokinetic study in patients with pretreated metastatic colorectal cancer. J Clin Oncol 1999, 17: 2901-8.

41. Saltz LB, Cox JV, Blanke C, et al. Irinotecan plus fluorouracil and leucovorin for metastatic colorectal cancer. Irinotecan Study Group. N Engl J Med 2000, 343: 905-14

42. de Gramont A, Figer A, Seymour M, et al. Leucovorin and fluorouracil with or without

oxaliplatin as first-line treatment in advanced colorectal cancer. : J Clin Oncol 2000, 18: 2938-47

43. Hebbar M, Tournigand C, Lledo G, et al. Phase II evaluation of an alternated FOLFOX /

FOLFIRI regimen in patients with resistant metastatic colorectal cancer (CRC). Proc ECCO 2001

44. Nordic Gastrointestinal Tumor Adjuvant Therapy Group. Expectancy or primary chemotherapy in patients with advanced asymptomatic colorectal cancer : a randomized trial. J Clin Oncol 1992, 10: 904-11

45. Scheithauer W, Rosen H, Kornek GV, Sebesta C, Depisch D. Randomized comparison of combination chemotherapy plus supportive care with supportive care alone in patients with metastatic colorectal cancer. Br J Med 1993, 306: 752-5

46. Zeghari-Squalli N, Raymond E, Cvitkovic E, Goldwasser F. Cellular pharmacology of the combination of the DNA topoisomerase I inhibitor SN-38 and the diaminocyclohexane platinum derivative oxaliplatin. Clin Cancer Res 1999, 5: 1189-96

47. Minagawa M, Makuuchi M, Torzilli G, Takayama T, Kawasaki S, Kosuge T, Yamamoto J, Imamura H. Extension of the frontiers of surgical indications in the treatment of liver metastases from colorectal cancer: long-term results. Ann Surg 2000, 231 : 487-99

48. Bolton JS, Fuhrman GM. Survival after resection of multiple bilobar hepatic metastases from colorectal carcinoma. Ann Surg. 2000, 231: 743-51

49. Fong Y, Fortner J, Sun RL, Brennan MF, Blumgart LH. Clinical score for predicting recurrence after hepatic resection for metastatic colorectal cancer: analysis of 1001 consecutive cases. Ann Surg 1999, 230: 309-18

50. Milano G, McLeod HL. Can dihydropyrimidine dehydrogenase impact 5-fluorouracil-based treatment? Eur J Cancer 2000, 36: 37-42

51. Collie-Duguid ES, Etienne MC, Milano G, McLeod HL. Known variant DPYD alleles do not explain DPD deficiency in cancer patients. Pharmacogenetics 2000, 10: 217-23

52. van Kuilenburg AB, Muller EW, Haasjes J, Meinsma R, Zoetekouw L, Waterham HR, Baas F, Richel DJ, van Gennip AH. Lethal outcome of a patient with a complete dihydropyrimidine dehydrogenase (DPD) deficiency after administration of 5-fluorouracil: frequency of the common IVS14+1G>A mutation causing DPD deficiency. Clin Cancer Res 2001, 7: 1149-53

53. Kang SS, Wong PW, Zhou JM, Sora J, Lessick M, Ruggie N, Grcevich G. Thermolabile methylenetetrahydrofolate reductase in patients with coronary artery disease. Metabolism 1988, 37: 611-3

54. Weisberg I, Tran P, Christensen B, Sibani S, Rozen R. A second genetic polymorphism in methylenetetrahydrofolate reductase (MTHFR) associated with decreased enzyme activity. Mol Genet Metab 1998, 64: 169-72

55. Cohen V, Panet-Raymond V, Sabbaghian N, Morin I, Batist G, Rozen R. Methylenetetrahydrofolate reductase polymorphism in advanced colorectal cancer: a novel genomic predictor of clinical response to fluoropyrimidine-based chemotherapy. Clin Cancer Res 2003, 9: 1611-5

56. Peters GJ, Backus HH, Freemantle S, et al. Induction of thymidylate synthase as a 5- fluorouracil resistance mechanism. Biochim Biophys Acta 2002, 1587: 194-205

57. Horie N, Aiba H, Oguro K, Hojo H, Takeishi K. Functional analysis and DNA polymorphism of the tandemly repeated sequences in the 5'-terminal regulatory region of the human gene for thymidylate synthase. Cell Struct Funct 1995, 20: 191-7

58. Mandola MV, Stoehlmacher J, Muller-Weeks S, Cesarone G, Yu MC, Lenz HJ, Ladner RD. A novel single nucleotide polymorphism within the 5' tandem repeat polymorphism of the thymidylate synthase gene abolishes USF-1 binding and alters transcriptional activity. Cancer Res 2003, 63: 2898-904

59. Villafranca E, Okruzhnov Y, Dominguez MA, et al. Polymorphisms of the repeated

sequences in the enhancer region of the thymidylate synthase gene promoter may predict

downstaging after preoperative chemoradiation in rectal cancer. J Clin Oncol 2001, 19: 1779-86

60. Etienne MC, Chazal M, Laurent-Puig P, et al. Prognostic value of tumoral thymidylate synthase and p53 in metastatic colorectal cancer patients receiving fluorouracil-based chemotherapy: phenotypic and genotypic analyses. J Clin Oncol 2002, 20: 2832-43

61. Ulrich CM, Bigler J, Velicer CM, Greene EA, Farin FM, Potter JD. Searching expressed sequence tag databases: discovery and confirmation of a common polymorphism in the thymidylate synthase gene. Cancer Epidemiol Biomarkers Prev 2000, 9: 1381-5

62. Dusinska M, Ficek A, Horska A, et al. Glutathione S-transferase polymorphisms influence the level of oxidative DNA damage and antioxidant protection in humans. Mutat Res 2001, 482: 47-55

63. Stoehlmacher J, Park DJ, Zhang W, Groshen S, Tsao-Wei DD, Yu MC, Lenz HJ. Association between glutathione S-transferase P1, T1, and M1 genetic polymorphism and survival of patients with metastatic colorectal cancer. J Natl Cancer Inst 2002, 94: 936-42

64. Park DJ, Stoehlmacher J, Zhang W, Tsao-Wei DD, Groshen S, Lenz HJ. A Xeroderma

pigmentosum group D gene polymorphism predicts clinical outcome to platinum-based chemotherapy in patients with advanced colorectal cancer. Cancer Res 2001, 61: 8654-8

65. Iyer L, King CD, Whitington PF, Green MD, Roy SK, Tephly TR, Coffman BL, Ratain MJ. Genetic predisposition to the metabolism of irinotecan (CPT-11). Role of uridine diphosphate glucuronosyltransferase isoform 1A1 in the glucuronidation of its active metabolite (SN-38) in human liver microsomes. J Clin Invest. 1998, 101: 847-54

66. Iyer L, Hall D, Das S, Mortell MA, Ramirez J, Kim S, Di Rienzo A, Ratain MJ. Phenotypegenotype correlation of in vitro SN-38 (active metabolite of irinotecan) and bilirubin glucuronidation in human liver tissue with UGT1A1 promoter polymorphism. Clin Pharmacol Ther. 1999, 65: 576-82

67. Innocenti F, Iyer L, Ratain MJ. Pharmacogenetics: a tool for individualizing antineoplastic therapy. Clin Pharmacokinet. 2000, 39: 315-25

68. Innocenti F, Ratain MJ. Update on pharmacogenetics in cancer chemotherapy. Eur J Cancer. 2002, 38: 639-44

**ANNEXES**

Annexe I : Liste des investigateurs

Annexe II : Indice de performance

Annexe III : Echelle de Lévi

Annexe IV : Toxicité NCI/CTC

Annexe V : Lettre d’information au patient

Annexe VI : Consentement éclairé

Annexe VII : Déclaration d’événement indésirable grave

Annexe VIII : Critères d’évaluation de réponse RECIST

Annexe IX : questionnaire de qualité de vie EORTC-QLQC30

Annexe X : Assurance

Annexe XI : Avis du CCPPRB

Annexe XII : Note d’information à l’intention du personnel hospitalier et du personnel de laboratoire et fiche d’inclusion pour les explorations de pharmacogénétique

**LISTE DES INVESTIGATEURS**

**Investigateur Principal :**

Pr. HEBBAR Mohamed

CHU de Lille – Service de médecine interne

1, place de Verdun

59 037 LILLE Cedex

**Investigateurs :**

Dr ACHILLE Emmanuel

Clinique de l’Orangerie

39, allée de la Robertsau

67000 STRASBOURG

Dr. ANDRE Thierry

Hôpital pitié Salpêtriere

Service d’hépato Gastro entérologie

47-83, bd de l'Hôpital

75651 PARIS Cedex 13

Dr ANGELLIER Elisabeth

Hôpital Fontenoy

BP 408

28018 CHARTRES CEDEX

Dr ARTRU Pascal

Clinique Saint Jean

35 rue Bataille

69008 LYON

Dr AUBY Dominique

Hôpital Robert Boulin

112 rue de la Marne

33500 LIBOURNE

Dr AZZEDINE Ahmed

Centre hospitalier d'Avignon

service onco-hématologie

305 rue Raoul Follereau

84000 AVIGNON

Dr BELGHITI Jacques

Fédération Médico-Chirurgicale d'Hépato-gastroentérologie

Hôpital Beaujon

100 boulevard Leclerc

92118 CLICHY cedex

Dr BENNAMOUN Mostefa

Centre des traitements des tumeurs

Hôpital Le Raincy- Montfermeil

10, rue du Général Leclerc

93370 MONTFERMEIL

Dr BELLAICHE-MICCIO Annie

Centre hospitalier de Lagny

31, avenue du Général Leclerc

77405 LAGNY SUR MARNE cdx

Dr MESIERES Marie

Hôpital François Maillot

54150 BRIEY

Dr CALLET Bertrand

Hôpital américain de Paris

63, boulevard Victor Hugo

92202 NEUILLY-SUR-SEINE cedex

Dr. COEFFIC David

Clinique du Mail

45 av Marie Reynoard

38034 GRENOBLE

Dr. CAROLA Elisabeth

CH de Senlis

Avenue Paul Rougé

60 309 SENLIS Cedex

Dr CAROLA Elisabeth

Clinique des Jockeys

12 av Général Leclerc

60631 CHANTILLY cedex

Dr DALIVOUST Philippe

Clinique de la Casamance

33 bd des Farigoules

13400 AUBAGNE

Pr. DE GRAMONT Aimery

Hôpital St Antoine – Service du Pr. KRULIK

184, rue du fbg St Antoine

75 571 PARIS Cedex 12

Dr DUTEL Jean Luc

CH Beauvais – service hémato-oncologie

40 avenue Léon Blum

60021 BEAUVAIS

Dr FAIVRE Sandrine

Fédération Médico-Chirurgicale d'Hépato-gastroentérologie

Hôpital Beaujon

100 boulevard Leclerc

92118 CLICHY cedex

Dr FARGES Olivier

Fédération Médico-Chirurgicale d'Hépato-gastroentérologie

Hôpital Beaujon

100 boulevard Leclerc

92118 CLICHY cedex

Dr FAROUX Roger

Centre Hospitalier Départemental Les Oudairies

85025 LA ROCHE S/YON Cedex

Dr FINCK Michel

34, avenue de Flandres

59170 CROIX

Dr. FLESCH Michel

Hôpital Drevon – Service Oncologie

8, rue des Princes de Condé

21 000 DIJON

Dr GASNAULT Laurent et Dr VINCENDET Marc

Clinique Côte d’Opale

173, route de Desvres

62280 SAINT MARTIN LEZ BOULOGNE

Dr GOERE Diane

Fédération Médico-Chirurgicale d'Hépato-gastroentérologie

Hôpital Beaujon

100 boulevard Leclerc

92118 CLICHY cedex

Dr GUICHARD Pierre

Polyclinique des Quatre Pavillons

15, rue Edouard Herriot

33 310 LORMONT

Dr HAMMEL Pascal

Fédération Médico-Chirurgicale d'Hépato-gastroentérologie

Hôpital Beaujon

100 boulevard Leclerc

92118 CLICHY cedex

Dr HENTIC Olivia

Fédération Médico-Chirurgicale d'Hépato-gastroentérologie

Hôpital Beaujon

100 boulevard Leclerc

92118 CLICHY cedex

Dr JACOB Jacques-Henri

Centre régional F.Baclesse

Route de Lion sur Mer

14076 CAEN cedex

Dr LANDI Bruno

Hôpital Européen Georges Pompidou

20, rue Leblanc

75 015 PARIS

Pr De LEDINGHEN Victor

Hôpital de Haut Levêque

5 av de Magellan

33604 PESSAC Cedex

Dr. MABRO May

Hôpital FOCH – Onco-Hématologie

40, rue Worth

92 151 SURESNES Cedex

Pr YCHOU Marc

Unité d’oncologie digestive

Centre Val d’Aurelle Paul Lamarque

34298 MONTPELLIER Cedex 5

Dr. MANDET Jacques

Clinique de Radiothérapie St Faron

Rue Charles de Gaulle

77 110 MAREUIL LES MEAUX

Dr. MARTIN Philippe

Clinique du bois

59 800 LILLE

Dr MERAD Zoher

Centre Hospitalier de Calais

11 quai du Commerce

62100 CALAIS

Dr MINEUR Laurent

Clinique Ste Catherine

Chemin du Lavarin

84000 AVIGNON cedex 2

Dr MINIER Béatrice

Centre Victor Hugo

9 bd de la République

16000 Angoulême

Dr MOUTEL CORVIOL karinne

Clinique de Courlancy

38, rue de Courlancy

51100 REIMS

Dr PAITEL Jean François

CH de la Rochelle

Rue du Dr Schweitzer

17019 LA ROCHELLE CEDEX

Dr PERRIER Hervé

Hôpital Saint Joseph

26 boulevard Louvain

13008 MARSEILLE

Dr RAYMOND Eric

Fédération Médico-Chirurgicale d'Hépato-gastroentérologie

Hôpital Beaujon

100 boulevard Leclerc

92118 CLICHY cedex

Dr. ROQUES Bertrand

Hôpital d’Annecy – Service d’Oncologie

1, avenue Trésums

74 011 ANNECY

Dr SABATE Jean-Marc

Version 1.12. février 2008 61

Hôpital Louis Mourier

178, rue des Renouillers

92700 Colombes cedex

Dr SAUVANET Alain

Fédération Médico-Chirurgicale d'Hépato-gastroentérologie

Hôpital Beaujon

100 boulevard Leclerc

92118 CLICHY cedex

Dr SMITH Denis

Hôpital Saint André

1 rue Jean Burguet

33075 BORDEAUX

Dr TAIEB Julien

Service d'hépato-gastro-entérologie

Groupe Hospitalier Pitié Salpétrière

47-83 Bd de L'Hôpital

75013 PARIS

Dr. VAILLANT Eric

Clinique Ambroise Paré

59 800 LILLE

Dr WALTER Sabine

Hôpital Notre Dame du Bon Secours

1, place Philippe Vigneulles

57038 METZ CEDEX

Dr WENDEHENNE Frédéric

Clinique Charcot

51/53 rue du Commandant Charcot

69 110 Ste FOY Les LYON

**ANNEXE II : INDICE DE PERFORMANCE**

**ANNEXE III : ECHELLE SPECIFIQUE DE NEUROTOXICITE DE LEVI**

**ANNEXE IV : TOXICITE NCI /CTC**

* local = infusion site reaction

**ANNEXE V : Notice d’information aux patients**

ETUDE DE PHASE III DE CHIMIOTHERAPIE

PAR FOLFOX 4 OU PAR UNE SUCCESSION FOLFOX 7-FOLFIRI

CHEZ DES PATIENTS AYANT DES METASTASES RESECABLES

D’ORIGINE COLORECTALE

Madame, Monsieur,

Il vous est proposé de participer à une recherche biomédicale : “Etude de phase III de chimiothérapie (étude comparative) par FOLFOX 4 ou par une succession FOLFOX 7 – FOLFIRI chez des patients ayant des métastases résécables d’origine colorectale”.

Cette étude sera conduite par votre médecin :

Docteur : ………………….;

N° de téléphone : ………………….

Cette notice d’information a pour but de vous résumer les informations essentielles en rapport avec cette recherche. Lisez -la avec attention et n’hésitez pas à poser toutes les questions qui vous semblent nécessaires.

**Quel est le traitement proposé ?**

Ce traitement vous est proposé en complément d’une exérèse chirurgicale de métastases hépatiques. Des données récentes suggèrent le caractère bénéfique de la chimiothérapie pour la diminution du risque de récidive, mais les meilleures modalités de la chimiothérapie ne sont pas encore déterminées dans cette situation. Il s’agit de l’objectif de cette étude.

Cette étude est une étude de phase III dont la caractéristique principale est de comparer deux traitements : un schéma de type FOLFOX 4 et un schéma comprenant d’abord un protocole

FOLFOX 7 ensuite un protocole FOLFIRI. Les études antérieures ont démontré que les séquences FOLFOX et FOLFIRI, administrées de façon isolée, permettent d’obtenir de bons résultats en terme de régression tumorale chez les patients atteints de cancer colorectal avec métastases. En outre, l’analyse approfondie des caractéristiques scientifiques de l’oxaliplatine et de l’irinotécan, plaide en faveur d’une potentialisation des effets bénéfiques des deux molécules et d’une limitation des effets indésirables de chacune d’elles.

Le traitement sera représenté soit par 12 cycles de FOLFOX 4, soit par 6 cycles de FOLFOX 7 puis 6 cycles de FOLFIRI.

Pour respecter un parfait équilibre entre les deux groupes de patients, votre traitement sera attribué par tirage au sort à l’aide de l’outil informatique.

Quel que soit le traitement qui vous sera alloué, une cure est prévue tous les 15 jours et comportera une perfusion d’une durée totale de 48 heures.

**Quels sont les effets indésirables potentiels ?**

Comme tout traitement médical, les molécules utilisées dans cette étude peuvent entraîner des effets indésirables. Mais dans tous les cas, l’équipe soignante possède des moyens pour prévenir ou guérir ces effets (par exemple casques réfrigérants prévenant la chute des cheveux, traitement antiémétisant prévenant ou soulageant les nausées/vomissements).

Les protocoles FOLFOX 4 ou 7 expose à un risque de chute du taux de globules blancs, de diarrhée, de stomatite et de réactions cutanées, auxquels s’ajoutent des risques de nausées vomissements, et de toxicité neurologique se traduisant généralement par des fourmillements des doigts lors de l’exposition au froid. Cette atteinte neurologique est réversible en quelques jours, mais si elle devient gênante, la dose d’oxaliplatine sera adaptée.

Le protocole FOLFIRI expose à un risque de chute du taux de globules blancs, de diarrhée, de stomatite et de réactions cutanées, auxquels s’ajoutent des risques (faibles) de nausées et de vomissements et de chute des cheveux.

**Quelle est la durée du traitement ?**

Plusieurs hypothèses peuvent motiver l’arrêt de ce traitement :

♉ votre propre demande

♉ l’administration de 12 cures

♉ l’expérience d’une toxicité justifiant le changement de traitement

♉ les résultats de l’intervention chirurgicale (lorsque la chimiothérapie est entreprise avant l’intervention)

Le médecin répondra à toutes vos questions.

**Comment se traduit la surveillance durant l’étude ?**

Pour évaluer l’efficacité du traitement et garantir votre sécurité, des prélèvements biologiques sont prévus, notamment la veille des cures de chimiothérapie. Des évaluations radiologiques (scanner et radiographie de thorax) planifiés à intervalles réguliers permettront de juger de l’évolution de votre maladie. Pour cela, il est nécessaire de posséder des données avant le début du traitement ; elles serviront de référence lors des évaluations ultérieures.

Il est également important de nous signaler tous les effets éventuels ressentis entre deux cures de chimiothérapie pour juger de la tolérance du traitement. Pour cela, nous vous demandons également de nous indiquer précisément les traitements médicamenteux que vous prenez occasionnellement ou qui vous sont prescrits par votre médecin traitant.

Enfin, un questionnaire “qualité de vie” vous sera proposé et permettra de juger de l’évolution de votre état de santé et de recueillir votre propre ressenti.

Cette étude a été conçue par des médecins spécialisés dans la prise en charge des cancers.

D’autre part, nous vous rappelons que cette étude a reçu l’avis favorable du Comité Consultatif pour la Protection des Personnes dans la Recherche Biomédicale de Lille en date du ………….

Nous vous remercions d’avoir pris le temps de lire cette notice d’information. Veuillez la conserver dans un endroit sûr au cas où vous souhaiteriez vous y référer ultérieurement. Votre médecin est là pour répondre à toutes les questions que vous pourriez vous poser par rapport à cette étude.

En cas d’urgence et si vous aviez besoin de conseils supplémentaires, ou de renseignements, veuillez nous contacter au n° de téléphone suivant : ………………………

**ANNEXE VI : Formulaire de consentement**

ETUDE DE PHASE III DE CHIMIOTHERAPIE

PAR FOLFOX 4 OU PAR UNE SUCCESSION FOLFOX 7-FOLFIRI

CHEZ DES PATIENTS AYANT DES METASTASES RESECABLES

D’ORIGINE COLORECTALE

Le Docteur ……………… (n° de téléphone : ……………………) m’a proposé un traitement faisant l’objet d’un protocole de recherche. L’étude en question vise à comparer deux traitements en terme de tolérance et d’efficacité selon un calendrier identique. J’ai reçu toutes les informations concernant les conditions de traitement, sa durée, son efficacité, les bénéfices attendus et les effets secondaires indésirables éventuels. Le médecin m’a proposé d’y réfléchir.

Je donne librement mon consentement pour participer à cette étude. Le protocole a notamment obtenu l’accord du Comité Consultatif pour la Protection des Personnes dans la Recherche Biomédicale de Lille le …… et le promoteur a souscrit une police d’assurance adaptée à cette recherche.

J’accepte que les données enregistrées à l’occasion de cette étude puissent faire l’objet d’un traitement informatisé de façon strictement anonyme. J’ai bien noté que le droit d’accès prévu par la loi “Informatique et Libertés” (article 40) s’exerce à tout moment auprès de mon médecin et je pourrai exercer mon droit de rectification.

Je suis libre d’accepter ou de refuser ce traitement à tout moment sans avoir à me justifier et sans conséquence sur la suite de mon suivi médical. Tout autre traitement pourra m’être proposé.

Mon consentement ne décharge en rien les organisateurs de la recherche de leurs responsabilités et n’affecte aucunement mes droits légaux.

J’ai lu et compris la notice d’information. J’ai bien compris et accepte le fait que l’allocation de l’un ou l’autre du traitement se fera par tirage au sort grâce à l’outil informatique. J’ai pu poser toutes les questions qui me semblaient nécessaires et ai obtenu des réponses satisfaisantes.

Fait à ……………

Date : .…/…./…. Date :…./.…/….

Nom du patient : ……………. Nom de l’investigateur : ………

Signature : Signature :

**ANNEXE VII : DECLARATION D’EVENEMENT INDESIRABLE GRAVE**

**ANNEXE VIII : CRITERES D’EVALUATION DE REPONSE RECIST**

"New Guidelines to evaluate the response to treatment in solid tumors". Therasse P, Arbuck SG, Eisenhauer EA et al. JNCI 2000; 92:205-16

*Méthodes de mesures*

La même méthode d'évaluation et la même technique doivent être utilisées pour toutes les lésions identifiées et suivies à l'inclusion et au cours de l'étude.

Examen clinique

Seules les lésions superficielles (par exemple: nodules cutanés ou palpables) peuvent être considérées comme mesurables. Une documentation photographique est recommandée en cas de lésion cutanée.

Radiographie pulmonaire

Les lésions peuvent être considérées comme mesurables lorsqu'elles sont clairement définies et entourées de parenchyme aéré.

Scanner et IRM

Ce sont les méthodes recommandées pour le suivi de l'évolution des lésions.

Sauf contre-indication particulière, un agent de contraste doit être administré.

Echographie

L'échographie n'est utilisée que pour la mesure des lésions accessibles à l'examen clinique (atteintes cutanées, sous-cutanées).

Endoscopie et laparoscopie

Ces techniques non encore validées et non standardisées ne sont utilisées que pour la confirmation de réponse complète histologique.

Marqueurs tumoraux

Ils ne peuvent être pris comme unique méthode d'évaluation de réponse. En cas de disparition de toutes les lésions à l'imagerie, les marqueurs devront être revenus dans les limites de la normale pour que la réponse soit dite complète.

Cytologie et histologie

Les techniques cytologiques et histologiques peuvent être utilisées pour différencier une réponse partielle d'une réponse complète dans quelques rares cas ou pour évaluer la nature d'un épanchement.

*Lésions à l’inclusion:*

Maladie mesurable

Les lésions dont le plus grand diamètre est > 2 cm au scanner classique ou 1cm au scanner spiralé.

Maladie non-mesurable

Les petites lésions (plus grand diamètre < 2 cm au scanner classique ou 1cm au scanner spiralé) .

Les lésions réellement non-mesurables : lésions osseuses, maladie leptoméningée, ascite, pleurésie, péricardite, maladie inflammatoire du sein, lymphangites carcinomateuses pulmonaires ou cutanées, les masses abdomino-pelviennes décelées par l’examen clinique mais non confirmées à l’imagerie et les lésions cystiques.

Lésions cibles

Les cibles choisies au cours de l'étude clinique sont au maximum 10 au total et au maximum 5 par organe. Elles sont représentatives de tous les organes envahis.

Sont choisies les lésions qui ont le plus grand diamètre et qui pourront être suivies tout au long de l'essai avec la méthode utilisée.

C’est la somme des plus grand diamètres de ces lésions cibles qui sera suivie au long de l’essai.

Lésions non-cibles

Toutes les autres lésions sont identifiées comme lésions non cibles et sont également relevées à l’inclusion. Elles ne sont pas mesurées mais sont suivies tout au long de l’essai

*Critères de réponse au traitement:*

*Lésions cibles*

Réponse complète (RC)

Disparition de toute lésion et normalisation des marqueurs antitumoraux

Réponse partielle (RP)

Diminution d'au moins 30 % de la somme des diamètres mesurables.

Stabilisation (MS)

Diminution < 30 % ou augmentation < 20 % de la somme des diamètes mesurables.

Progression (MP)

Augmentation > 20 % de la somme des diamètres mesurables par rapport à la plus petite valeur observée, ou apparition d'une nouvelle lésion.

**Lésions non cibles**

Réponse complète (RC)

Disparition de toute lésion et normalisation des marqueurs antitumoraux

Réponse incomplète – Stabilisation (RI)

Persistance d’au moins une lésion non cible et/ou marqueur élevé.

Progression (MP)

Augmentation non-équivoque de la taille des lésions non cibles ou apparition d'une nouvelle lésion.

*Réponse globale*

Toute réponse complète ou partielle devra être confirmée dans un délai > 4 semaines après son obtention. Une réponse nonconfirmée ne pourra être considérée comme une réponse.

Un panel de radiologistes indépendants expertisera les patients.

*La meilleure réponse globale*

La meilleure réponse globale est la meilleure réponse enregistrée depuis le début du traitement jusqu'à la progression.

*Durée de réponse*

La durée de réponse globale est le temps entre la réponse partielle ou complète (quelle que soit celle qui est observée en premier) et la progression (en prenant comme référence, pour la progression, la plus petite valeur enregistrée depuis le début du traitement).

La durée de réponse complète est le temps entre la première réponse complète enregistrée et la progression.

*Durée de stabilité*

La durée de stabilité est le temps entre le début du traitement et la progression (en prenant comme référence pour la progression la plus petite valeur enregistrée depuis le début du traitement). Un intervalle minimum entre l'entrée dans l'étude et la prise encompte de la stabilité sera défini protocolairement, il est généralement de 6-8 semaines.

**ANNEXE IX : questionnaire de qualité de vie EORTC-QLQC30**

**ANNEXE X : ASSURANCE**

**ANNEXE XI : AVIS DU CCPPRB**

**ANNEXE XI : Note d’information à l’intention du personnel hospitalier et du personnel de laboratoire et fiche d’inclusion pour les explorations de pharmacogénétique**

**ESSAI MIROX**

**Note d'information au patient et Consentement éclairé**

**Protocole de pharmacogénétique concernant une éventuelle susceptibilité**

**de nature génétique vis-à-vis des effets des chimiothérapies**

Madame, Monsieur,

Votre maladie nécessite un traitement médicamenteux. Dans ce cadre, nous vous proposons de participer à une recherche dont l'objectif est d'étudier l'existence de liens entre certaines caractéristiques de vos gènes et les effets du traitement médicamenteux qui vous sera administré.

Cette étude consistera à analyser, sur l'ADN de votre sang, des gènes liés à l'effet du traitement qui vous sera administré, afin de mettre en évidence une éventuelle susceptibilité individuelle vis-à-vis du traitement. Afin de mener cette recherche et de pouvoir en tirer des conclusions, nous sommes amenés à étudier plusieurs cas semblables au vôtre.

Cette étude nécessite un prélèvement sanguin de faible volume (8,5 ml) qui pourra être réalisé selon votre convenance (aucune nécessité d'être à jeun, ni aucune contrainte d'aucun ordre).

Votre participation à cette recherche ne modifiera en rien votre traitement, ni votre prise en charge par votre médecin traitant. Votre participation à cette recherche est totalement facultative.

Vous êtes libre d'accepter ou de refuser d'y participer sans que cela nuise à la qualité de votre relation avec votre médecin. Si vous acceptez, votre dossier médical restera naturellement confidentiel et ne pourra être consulté, sous la responsabilité du médecin s'occupant de votre traitement, que par des personnes soumises au secret professionnel. Toutes les données recueillies au cours de l'étude seront enregistrées anonymement par un traitement informatisé soumis à la loi “Informatique et Liberté ” qui garantit la confidentialité de toutes les données.

Votre consentement ne décharge pas les organisateurs de l'étude de leurs responsabilités et vous conserverez tous vos droits garantis par la loi.

En accord avec la loi du 2 mars 2002, les résultats globaux de cette recherche pourront être communiqués aux participants qui le souhaitent. Ces résultats relèvent du secret médical et ne seront transmis qu'à vous-même et en aucun cas à un autre membre de votre famille.

Après lecture de la note d'information, je déclare :

1- Avoir été informé(e) de la nature de l'étude et de ses objectifs.

2- Avoir noté que ma participation à cette recherche est totalement libre.

3- Avoir reçu toutes les réponses souhaitées à mes questions.

4- Avoir noté que les données me concernant resteront strictement confidentielles. Je n'autorise leur consultation que par des personnes qui collaborent à cette recherche, et éventuellement un représentant des Autorités de Santé.

5- Avoir été informé(e) conformément à la Loi 95-548 du 1er juillet 1994 et au décret n° 95-682 du 9 mai 1995 que des données nominatives me concernant feront, pour cette recherche, l'objet d'un traitement informatisé.

6- Avoir été informé(e) :

- de la nature des informations transmises (caractéristiques de ma maladie, évaluation de l'efficacité et des effets secondaires éventuels de mon traitement médicamenteux, résultats des analyses génétiques réalisées à partir de mon prélèvement sanguin).

- de la finalité du traitement des données (recherche de liens entre les caractéristiques des gènes analysés et les effets du traitement).

- de mon droit d'accès et de rectification, soit directement, soit indirectement par l'intermédiaire d'un médecin de mon choix désigné à cet effet.

- de mon droit de m'opposer au traitement automatisé des données nominatives me concernant.

7- Avoir été avisé de ce que la présentation du résultat du traitement des données ne pourra permettre mon identification directe ou indirecte.

Je soussigné(e) (Nom, Prénom). ..............................................................……………………..

déclare avoir pris connaissance de la Note d'information et accepte par la présente de participer à la recherche que m'a proposée le Docteur ………………………..….. en toute connaissance de cause et en toute liberté.

Le Docteur ………………………………… m'a précisé que je suis libre d'accepter ou de refuser de participer à cette recherche, mon refus de participer ne modifiant en rien nos relations, ni l'attention médicale et la qualité des soins qui me seront apportées.

Date :

Signature du patient :

Signature du médecin :

*Document établi en double exemplaire dont un remis au patient*

**ESSAI MIROX**

**Explorations de pharmacogénétique**

**chez des patients traités par chimiothérapie**

**pour un cancer colorectal**

*Note d'information à l'atttention*

*du personnel infirmier et du personnel de laboratoire pour*

*les explorations de pharmacogénétique*

**Prélever 10 ml de sang sur tube EDTA** (référence Greiner GR10K3). Cette prise de sang peut être effectuée à n'importe quel moment par rapport au traitement et ne nécessite pas d'être à jeun.

Le tube de sang prélevé sera dûment **identifié au crayon noir** (mine carbone ordinaire), directement sur l'étiquette d'origine du tube (ne pas coller d'étiquette, qui ne tiendrait pas durant le transport dans la carboglace) en précisant le nom, prénom du patient + date de prélèvement. **Le tube de sang ne sera pas centrifugé.** Il sera **congelé tel quel à -20°C** jusqu'au moment de son expédition au Centre Antoine Lacassagne.

Les tubes seront regroupés tous les 6 à 12 mois, en fonction du recrutement, afin de minimiser les coûts d'acheminement. Les tubes seront envoyés accompagnés des "**fiches** **d'inclusion pour pharmacogénétique"** dûment complétées, au Laboratoire

d'Oncopharmacologie du Centre Antoine Lacassagne (adresse ci-dessous, transport dans carboglace sous 24h), selon la réglementation en vigueur (transporteur agrée pour les prélèvements biologiques potentiellement infectieux, classe 6.2 ONU N° 2814). Pour le transport, chaque tube sera individuellement emballé dans une pochette plastique (afin de minimiser les problèmes d'identification consécutifs au décollement des étiquettes).

Laboratoire d'Oncopharmacologie

Mr FORMENTO ou Mme FRANCOUAL

Centre Antoine Lacassagne

33 Av de Valombrose

06 189 Nice cedex 2

Tel : 04 92 03 15 57 ou 04 92 03 15 54

**ESSAI MIROX**

**Fiche d'inclusion pour pharmacogénétique**

***A adresser en même temps que le prélèvement sanguin****

***À Jean-Louis FORMENTO ou Mireille FRANCOUAL***

*Laboratoire d' Oncopharmacologie, Centre Antoine Lacassagne*

*33 Av de Valombrose, 06189 Nice cedex 2*

*Tel : 04 92 03 15 57*

*Fax :04 93 81 71 31*

Centre investigateur : ........................................................................................................

Identification du patient :

Nom :

……………………………………………………………………………………

Prénom

:……………………………………………………………………………..……

N° du patient dans l'essai : …………………

Date de réalisation du prélèvement sanguin (j/m/a) : ……. / …… / ……

* Le prélèvement sanguin sera réalisé sur un tube 10 ml EDTA (Greiner ref GR10K3) congelé tel quel à -20°C. Le tube sera directement identifié au crayon graphite sur l'étiquette originale du tube (nom + prénom du patient + date prélèvement). Pour l'expédition dans la carboglace, chaque tube sera individuellement emballé dans une pochette en plastique. L'ensemble des tubes regroupés sera transmis au Laboratoire d'Oncopharmacologie du Centre Antoine Lacassagne (transport dans carboglace sous 24h), par un transporteur agrée pour les substances potentiellement infectieuses.
