# Supplementary material for: Time to Definitive Health-Related Quality of Life Score Deterioration in Patients with Resectable Metastatic Colorectal Cancer Treated with FOLFOX4 versus Sequential Dose-Dense FOLFOX7 followed by FOLFIRI: The MIROX Randomized Phase III Trial
Source: PLoS One. 2016 Jun 16;11(6):e0157067. doi: 10.1371/journal.pone.0157067 (PMC4910973; doi:10.1371/journal.pone.0157067)
Supplement: S2 Protocol — (DOC) [file pone.0157067.s003.doc]

S1B : English version

**FOLFOX4 versus sequential dose-dense FOLFOX7 followed by FOLFIRI in patients with resectable metastatic colorectal cancer. A GERCOR randomized phase III study (MIROX).**

Sponsor: GERCOR

Coordinator Principal: Professor Mohamed Hebbar

Professor Mohamed Hebbar, Service d’Oncologie Médicale, Centre Hospitalo-Universitaire, 1 rue Michel Polonovski, 59 037 Lille, FRANCE, Tel: 00 33 3 20 44 54 61, Fax: 00 33 3 20 44 50 23, Email: [mohamed.hebbar@chru-lille.fr](mailto:mohamed.hebbar@chru-lille.fr)

The United States National Health Institute registry identifier: NCT00268398.

**TRIAL DESCRIPTION**: Open-label, randomized phase III trial.

**PURPOSE**: To determine whether a sequential chemotherapy of 5-fluorouracil (5-FU)/leucovorin (LV) with dose-dense oxaliplatin (FOLFOX7) and irinotecan-based 5-FU/LV (FOLFIRI) is superior to FOLFOX4 in patients with resectable metastases from colorectal cancer (CRC).

**OBJECTIVES**:

***Primary***

• 2-year disease-free survival (DFS) rate.

***Secondary***

• Overall survival (OS).

• Toxicity (according to the National Cancer Institute Common Toxicity Criteria, version 2.0).

• Health-related quality of life (HRQoL).

• Objective response rate (ORR).

• In case of perioperative chemotherapy:

- Tumor response according to the RECIST 1.0
- Resection type (R0 vs. R1 vs. R2).

• Postoperative complication rate.

• Transfusing rate in patients having metastasis surgery.

• Pharmacogenetics.

**TREATMENT PLAN:**

Patients will be randomized to 1 of 2 treatment arms:

•Arm I (12 cycles of FOLFOX 4): Patients will receive FOLFOX 4 chemotherapy comprising leucovorin 200 mg/m^2^ by infusion over 2 hours on days 1- 2, oxaliplatin 85 mg/m^2^ by infusion over 2 hours on day 1, fluorouracil 400 mg/m^2^ over 15 min on days 1- 2, then fluorouracil 1200 mg/m^2^ over 46 hours. Treatment will repeat every 2 weeks for 12 courses in the absence of disease progression or unacceptable toxicity.

•Arm II (6 cycles of FOLFOX 7 and FOLFIRI): Patients will receive 6 cycles FOLFOX 7 combination chemotherapy comprising high-dose oxaliplatin 130mg/m^2^ infusion on day 1 in combination with leucovorin 200 mg/m^2^ by infusion over 2 hours followed by fluorouracil 2400 mg/m^2^ by continuous infusion over 46 hours followed by 6 cycles of FOLFIRI consisted of irinotecan 180 mg/m^2^ by 30-90 min infusion in combination with leucovorin 200 mg/m^2^, fluorouracil 400 mg/m^2^ over 15 min on day 1, followed by fluorouracil 2400 mg/m^2^ over 46 hours. Treatment will repeat every 2 weeks for 6 courses in the absence of disease progression or unacceptable toxicity.

Simple or complex surgical procedures will be authorized, provided resection will be complete or considered to be initially feasible in case of preoperative chemotherapy. Patients who will undergo R2 (residual tumor) resection will be taken off the study. In patients operated before the entry into the study, a R0 resection will be required.

**DOSE MODIFICATION**

Toxicity will be evaluated before each cycle and following dose modification will be used for subsequent cycles:

***FOLFOX4:***

If neurological symptoms will occur, the following oxaliplatin dosage adjustment will be recommended, based on the duration and severity:

- Paraesthesia associated with pain or functional impairment

- non persistent (≤7 days) - oxaliplatin won't be modified,
- persistent >7 days and <14 days,

- if neurologic examination normal – oxaliplatin dose will be reduced from 85 to 75 mg/m^2^,

- if neurologic examination abnormal - the cycle of therapy will be omitted with resumption of therapy at the scheduled next cycle, with oxaliplatin dose reduction to 75 mg/m^2^,

- persisted between cycles - therapy will be interrupted until improvement and resumed at 75 mg/m^2^ oxaliplatin.

- Neutropenia

grade 3/4 (NCI/CTC) – bolus 5-FU dose will be reduced from 400 to 300 mg/m^2^ and continuous 5-FU dose will be reduced from 600 to 500 mg/m^2^/day, oxaliplatin dose will be reduced from 85 to 75 mg/m^2^.

- Nausea and/or vomiting despite premedication -

grade 4 (NCI/CTC) - oxaliplatin dose will be reduced from 85 to 75 mg/m^2^ with antiemetics adjustment; if intolerable toxicity, patient will leave the study after agreement between the principal investigator and physician.

- Diarrhoea or mucositis

grade 3 (NCI/CTC) - bolus 5-FU dose will be reduced from 400 to 300 mg/m^2^ and continuous 5-FU dose will be reduced from 600 to 500 mg/m^2^/day,

grade 4 (NCI/CTC) - bolus 5-FU dose will be reduced from 400 to 300 mg/m^2^ and continuous 5-FU dose will be reducded from 600 to 500 mg/m^2^/day, oxaliplatin dose will be reduced from 85 to 75 mg/m^2^.

- Central neurological and cardiac toxicity (especially angina pectoris) - treatment discontinuation.

- Cutaneous toxicity

grade 3/4 (NCI/CTC) - bolus 5-FU dose will be reduced from 400 to 300 mg/m^2^ and continuous 5-FU dose will be reduced from 600 to 500 mg/m^2^/day.

- Other toxicity - bolus 5-FU dose will be reduced from 400 to 300 mg/m^2^ and continuous 5-FU dose will be reduced from 600 to 500 mg/m^2^/day, oxaliplatin dose will be reduced from 85 to 75 mg/m^2^.

- Persistent thrombocytopenia >75000/mm^3^ - treatment will be continued after decision of the principal investigator.

- If grade 3/4 toxicities persist, following cycles will be carried at the lower level, except in cases of uncomplicated neutropenia (<7 days, recovery after 15 days).

***FOLFOX7:***

If neurological symptoms will occur, the following oxaliplatin dosage adjustment will be recommended, based on the duration and severity:

- Paraesthesia associated with pain or functional impairment:

- non persistent (≤7 days) - oxaliplatin dose won’t be modified,
- persistent >7 days and <14 days : oxaliplatin dose will be reduced from 130 to 100 mg/m^2^,
- persisted between cycles – oxaliplatin will be interrupted. The patient will subsequently receive FOLFIRI for a total (FOLFOX + FOLFIRI) of 12 cycles.

- Neutropenia

grade 3/4 (NCI/CTC) - 5-FU: 2000mg/m^2^ after recovery, oxaliplatin: 100 mg/m^2^after recovery.

- Thrombopenia

grade 3/4 (NCI/CTC) – 5-FU: 2000mg/m^2^, oxaliplatin: 100 mg/m^2^ after recovery.

- Mucositis

grade 3/4 (NCI/CTC) – 5-FU: 2000mg/m^2^, oxaliplatin: 100 mg/m^2^ after recovery.

- Diarrhoea -

grade 3/4 (NCI/CTC) - 5-FU 2000mg/m^2^.

Cutaneous toxicity

grade 3/4 (NCI/CTC) - 5-FU 2000mg/m^2^.

- Central neurological and cardiac toxicity (especially angina pectoris) - treatment discontinuation.

- Persistent thrombocytopenia >75000/mm^3^- treatment will be continued after decision of the principal investigators.

***FOLFIRI:***

- Neutropenia

grade 2 (NCI/CTC) - 5-FU: 100% after recovery, irinotecan 150 mg/m^2^,

grade 3/4 (NCI/CTC) – 5-FU: 2000mg/m² after recovery, irinotecan 150 mg/m^2^.

- Thrombopenia

grade 2 (NCI/CTC) – 5-FU: 100% after recovery, irinotecan 150 mg/m^2^,

grade 3 (NCI/CTC) – 5-FU: 2000mg/m² after recovery, irinotecan 150 mg/m^2^,

grade 4 (NCI/CTC) – patient will leave the study.

- Mucositis

grade 3/4 (NCI/CTC) – 5-FU: 2000mg/m^2^, irinotecan 150 mg/m^2^.

- Diarrhoea

grade 2 (NCI/CTC) - 1^st^ occurrence – 5-FU dose will be lowered and continued at 2000mg/m^2^, 2^nd^ occurrence irinotecan 150 mg/m^2^,

grade 3/4 (NCI/CTC) – 5-FU: 2000mg/m^2^, irinotecan 150 mg/m^2^.

- Cutaneous toxicity

grade 3 (NCI/CTC) - 5-FU 2000mg/m^2^,

grade 4 (NCI/CTC) - patient will leave the study.

- Angina pectoris - patient will leave the study.

- Persistent thrombocytopenia >75000/mm^3^- treatment will be continued after decision of the principal investigators.

- In case of persistent grade 3/4 toxicities, following cycles will be carried at the lower level, except in cases of uncomplicated neutropenia <7 days, and recovery after 15 days.

- In case of persistent diarrhoea grade >2 or severe neutropenia (grade 4 > 7 days or febrile neutropenia) despite decrease in irinotecan to 150 mg/m², the treatment will be discontinued and the patient will leave the study.

***MAIN ELIGIBILITY CRITERIA***

***DISEASE CHARACTERISTICS:***

• Histologically confirmed colon or rectal adenocarcinoma.

• Resectable or resected metastatic disease (liver, lung, ovary or peritoneum).

• Only one metastatic site will be allowed.

• Patients with peritoneum metastasis in case of single and completely resected metastasis.

• Radiofrequency ablation, alone or in combination with surgical resection, will authorized provided no more than 3 liver metastases with a maximum diameter <35 mm.

• Prior adjuvant chemotherapy after resection of the primary tumor will be allowed if the interval between the end of chemotherapy and relapse will be ≥12 months.

***PATIENT CHARACTERISTICS***:

• Age 18-75,

• Alkaline phosphatase ≤5 x upper limit of normal (ULN),

• Aspartate and alanine transaminases ≤3x ULN,

• Neutrophil count ≥2.0x10^9^/L,

• Platelet count ≥100x10^9^/L,

• Bilirubin ≤2 times normal,

• Creatinine ≤135 µmol/L or creatinine clearance ≥ 60mL/min,

• No peripheral neuropathy that affects normal functions,

• No unresolved complications from prior surgery,

• WHO performance status ≤2,

• Adequate hematologic, renal and liver functions.

***PRIOR OR CONCURRENT THERAPY:***

• At least 1 year since prior FOLFOX4 or FOLFIRI regimen in the adjuvant setting.

• No concurrent participation in another clinical trial.

• Recovered from prior therapy.

**RANDOM ASSIGNEMENT AND STRATIFICATION**

• Random assignment will be centralized and performed using an Interactive Voice Response System.

• Stratifying by:

- Treatment chronology (perioperative *vs.* postoperative chemotherapy),
- Type of local therapeutic intervention (surgery *vs.* radiofrequency ablation +/- surgery,
- Blumgart’s score (0-1 *vs.* 2-3 *vs.* 4-5).

**MAIN NOT EGLIBITITY CRITERIA**:

• Patients who have previously received chemotherapy for metastatic disease,

• Unresolved postoperative complication, or prior chemotherapy in the metastatic setting,

• Presence of cirrhosis,

• Presence of extrahepatic metastases or metastatic foci after radical treatment,

• Macroscopically incomplete resection of liver metastases,

• Pulmonary embolism or stroke within 6 months prior to entry into the study,

• Peripheral sensory neuropathy with functional impairment,

• Evidence of other diseases or medical conditions within 6 months prior to study,

• Patients participating in another study,

• Patients who cannot be monitored regularly,

• Pregnant of breast feeding woman.

**PROJECTED ACCRUAL**:

• A total of 284 patients, 142 per treatment arm.

**ENDPOINTS EVALUATION**

• DFS defined as the interval between randomization and first evidence of relapse (local, regional, metastases or second CRC), or death from any cause.

• OS defined as the interval between randomization and patient death from any cause.

• Toxicity assessed according to the National Cancer Institute Common Toxicity Criteria (NCI-CTC) criteria, version 2.0.

• Tumor response (complete response, partial response, stable disease, progressive disease), ORR and disease control rate assessed according to the RECIST 1.0 criteria.

• HRQoL assessed using the European Organization for Research and Treatment of Cancer (EORTC) self-administered Quality of Life Questionnaire (QLQ-C30) at baseline and at 8, 16 and 24 weeks.

• Time until definitive deterioration (TUDD) used for longitudinal analyses defined as the interval between randomization and the first observation of a definitive deterioration of QLQ-C30 score, the later including 5 points decrease in QoL score compared to the QoL score at baseline, no improvement in QoL score ≥5 points as compared with baseline or patient drop-out after ≥5 points decrease resulting in missing data or death.

**TREATMENT EVALUATION**

• Thoracic CT scan and abdominal CT or MRI scans will be performed within two weeks before inclusion.

• In patients who will undergo preoperative chemotherapy abdominal CT-scan will be repeated before surgery.

• Positron emission tomography scan is optional, while intra-operative ultrasound is recommended.

• MRI or CT-scans and carcinoembryonic antigen assay will be performed at baseline, before surgery and at the end of chemotherapy.

• Examinations will be repeated every 3 months for 2 years, and every 6 months thereafter.

**STATISTICAL ANALYSIS:**

***POWER OF TRIAL AND CLINICAL JUSTIFICATION***

• According to hypothesis that the 2-year DFS might be improved from 30% with FOLFOX4 chemotherapy to 45% with FOLFOX7-FOLFIRI (Hazard Ratio [HR] of 0.66), 15% difference using a 80% power and bilateral α type I error of 5%, 188 events is required. Based on estimated 36 months inclusion duration, and 24 months follow-up, at least 248 patients has to be enrolled. Assuming a drop-out rate around of 20% (disease progression before surgery, R2 resections or lost to follow-up), a total number of 284 patients, 142 per treatment arm, will be required.

***BASELINE CHARACTERISTICS***

• Described using frequency, and compared by means of Chi-square or Fischer’s tests.

***ANALYSIS OF EFFICACY*** ***AND SAFETY***

• Performed on all randomized patients who will receive at least one dose of chemotherapy with a use of a modified Intent-to-Treat (mITT) approach.

• Survivals and median follow-up will be estimated by the Kaplan-Meier and reverse Kaplan-Meir methods.

• Response rate will be presented with corresponding 95% CI for the median.

• Treatment outcomes will be compared using a log-rank test.

• Proportional hazard assumptions will be based on Schoenfeld residuals.

• TUDD will be evaluated using the Kaplan-Meier method.

**REASONS FOR EARLY CESSATION OF TRIAL:** Not applicable.
