# Supplementary material for: Time to Definitive Health-Related Quality of Life Score Deterioration in Patients with Resectable Metastatic Colorectal Cancer Treated with FOLFOX4 versus Sequential Dose-Dense FOLFOX7 followed by FOLFIRI: The MIROX Randomized Phase III Trial
Source: PLoS One. 2016 Jun 16;11(6):e0157067. doi: 10.1371/journal.pone.0157067 (PMC4910973; doi:10.1371/journal.pone.0157067)
Supplement: S1 Table — (DOC) [file pone.0157067.s004.doc]

S3:**Table A1**: Baseline characteristics of patients included according to treatment arm.

|  | **FOLFOX4** | | | | **FOLFOX7-FOLFIRI** | | |
| --- | --- | --- | --- | --- | --- | --- | --- |
|  | **(N = 142)** | | | | **(N = 142)** | | |
|  | N | | | % | N | | % |
| **Age** |  | | |  | |  |  |
| Median (Min-Max) | | 63.0 | (29-78) | | | 60.5 | (32-75) |
| **Gender** | |  |  | | |  |  |
| Male | | 91 | 64.1 | | | 99 | 69.7 |
| Female | | 51 | 35.9 | | | 43 | 30.3 |
| **Adjuvant chemotherapy** | |  |  | | |  |  |
| Yes | | 32 | 22.5 | | | 39 | 27.5 |
| No | | 110 | 77.5 | | | 103 | 72.5 |
| **WHO Performance status** | |  |  | | |  |  |
| 0 | | 99 | 72.8 | | | 91 | 66.4 |
| 1 | | 33 | 24.3 | | | 41 | 29.9 |
| 2 | | 4 | 2.9 | | | 5 | 3.7 |
| Not available | | 6 | - | | | 5 | - |
| **Primary** | |  |  | | |  |  |
| Colon | | 101 | 71.1 | | | 94 | 66.2 |
| Rectum | | 41 | 28.9 | | | 48 | 33.8 |
| **WHO Performance status** | |  |  | | |  |  |
| 0 | | 99 | 72.8 | | | 91 | 66.4 |
| 1 | | 33 | 24.3 | | | 41 | 29.9 |
| 2 | | 4 | 2.9 | | | 5 | 3.7 |
| Not available | | 6 | - | | | 5 | - |
| **Time from primary to metastases** | |  |  | | |  |  |
| ≥ 12 months | | 40 | 28.4 | | | 51 | 36.2 |
| < 12 months | | 101 | 71.6 | | | 90 | 63.8 |
| Not available | | 1 | - | | | 1 | - |
| **Total number of metastases (before surgery)** | |  |  | | |  |  |
| 0 | | 0 |  | | | 1 | 0.7 |
| 1 | | 70 | 49.3 | | | 69 | 48.6 |
| 2 | | 38 | 26.8 | | | 36 | 25.4 |
| 3 | | 21 | 14.8 | | | 19 | 13.4 |
| 4 | | 7 | 4.9 | | | 11 | 7.8 |
| ≥ 5 | | 6 | 4.2 | | | 6 | 4.2 |
| **Maximal metastases diameter** | |  |  | | |  |  |
| ≤ 5 cm | | 121 | 85.8 | | | 118 | 83.3 |
| > 5 cm | | 20 | 14.2 | | | 22 | 15.7 |
| Not available | | 1 | - | | | 2 | - |
| **Treatment strategy** | |  |  | | |  |  |
| Resected population (postoperative chemotherapy) | | 57 | 40.1 | | | 59 | 41.6 |
| Resectable population (perioperative chemotherapy) | | 85 | 59.9 | | | 83 | 58.5 |
| **Number of metastatic sites** | |  |  | | |  |  |
| 1 | | 132 | 93.0 | | | 138 | 97.2 |
| 2 | | 9 | 6.3 | | | 4 | 2.8 |
| 3 | | 1 | 0.7 | | | 0 |  |
| **Sites of metastases** | |  |  | | |  |  |
| Liver | | 118 | 83.1 | | | 118 | 83.1 |
| Lung | | 20 | 14.1 | | | 20 | 14.1 |
| Peritoneum | | 11 | 7.7 | | | 6 | 4.2 |
| Node | | 1 | 0.7 | | | 1 | 0.7 |
| Ovary | | 3 | 2.1 | | | 0 |  |
| Other site | | 0 | 0 | | | 1 | 0.7 |
| **Blumgart’s score** | |  |  | | |  |  |
| 0-1 | | 43 | 30.3 | | | 50 | 35.2 |
| 2-3 | | 90 | 63.4 | | | 82 | 57.8 |
| 4-5 | | 9 | 6.3 | | | 10 | 7.0 |
| **Serum CEA level** | |  |  | | |  |  |
| < 200 ng/Ml | | 131 | 93.6 | | | 134 | 95.7 |
| > 200 ng/Ml | | 9 | 6.4 | | | 6 | 4.3 |
| Not available | | 2 | - | | | 2 | - |

S4: Table A2: Baseline characteristics of patients enrolled on the MIROX study

**^*^**Fisher exact test

|  | **Patients with HRQoL data**  ***N* = 171** | **Patients without HRQoL data**  ***N* = 113** | **Total** | ***P*-value** |
| --- | --- | --- | --- | --- |
| Age, n (%) |  |  |  | 0.468 |
| <63 | 90 (53) | 54 (48) | 144 (51) |  |
| >=63 | 81 (47) | 59 (52) | 140 (49) |  |
| Gender, n (%) |  |  |  | 0.029 |
| Female | 48 (28) | 46 (41) | 94 (33) |  |
| Male | 123 (72) | 67 (59) | 190 (67) |  |
| Treatment arms, n (%) |  |  |  | 0.628 |
| FOLFOX4 | 83 (49) | 59 (52) | 142 (50) |  |
| FOLFOX7 + FOLFIRI | 88 (51) | 54 (48) | 142 (50) |  |
| Adjuvant chemotherapy, n (%) |  |  |  | 0.903 |
| Yes | 71 (42) | 46 (41) | 117 (41) |  |
| No | 100 (58) | 67 (59) | 167 (59) |  |
| Tumor site, n (%) |  |  |  | 0.602 |
| Colon | 115 (67) | 79 (70) | 194 (68) |  |
| Rectum | 56 (33) | 33 (29) | 89 (31) |  |
| Unknown | 0 (0) | 1 (1) | 1 (> 1) |  |
| Body surface area |  |  |  | 0.235 |
| ≤1.73 mg/mL | 49 (29) | 39 (35) | 88 (31) |  |
| >1.73 mg/mL | 120 (70) | 69 (61) | 189 (67) |  |
| Unknown | 2 (1) | 5 (4) | 7 |  |
| Symptoms |  |  |  | 0.017 |
| Yes | 62 (36) | 25 (22) | 87 (31) |  |
| No | 108 (63) | 86 (76) | 194 (68) |  |
| Unknown | 1 (1) | 2 (2) | 3 (1) |  |
| Performance status |  |  |  | 0.891 |
| 0 | 115 (67) | 73 (65) | 188 (66) |  |
| 1-2 | 51 (30) | 30 (26) | 81 (29) |  |
| Unknown | 5 (3) | 10 (9) | 15 (5) |  |
| Delay between diagnostic of the primary tumor and metastasis |  |  |  | 0.653 |
| Simultaneous | 53 (31) | 34 (30) | 87 (30) |  |
| 0.1-12 months | 67 (39) | 39 (34) | 106 (37) |  |
| >12 months | 51 (30) | 39 (34) | 90 (32) |  |
| Unknown | 0 (0) | 1 (1) | 1 (> 1) |  |

S5:Table A3: multivariate Cox analyses of TUDD analyses for QoL scores

|  |  |  |  |  |  |  |  |  |  |
| --- | --- | --- | --- | --- | --- | --- | --- | --- | --- |
|  | **n** | **Hazard ratio** | **(95%°CI)** | **p** |  | **n** | **Hazard ratio** | **(95%°CI)** | **p** |
|  |  | **fatigue** |  |  |  |  | **insomnia** |  |  |
| **Treatment arms** | *124(97)* |  |  |  |  | *124(69)* |  |  |  |
| FOLFOX alone |  | 1 |  |  |  |  | 1 |  |  |
| FOLFOX + FOLFIRI | | 1.12 | [0.74-1.69] | *0.584* |  |  | 0.82 | [0.51-1.34] | *0.448* |
| **Progression status(no)†** |  | 0.97 | [0.94-1.01] | *0.211* |  |  | 0.98 | [0.95-1.02] | *0.574* |
| **Localisation** |  |  |  |  |  |  |  |  |  |
| Colon |  | 1 |  |  |  |  |  |  |  |
| Rectum |  | 0.69 | [0.44-1.07] | *0.102* |  |  | _ | _ | *_* |
|  |  | **Role** |  |  |  |  | **social** |  |  |
| **Treatment arms** | *123(85)* |  |  |  |  | *122(85)* |  |  |  |
| FOLFOX alone |  | 1 |  |  |  |  | 1 |  |  |
| FOLFOX + FOLFIRI | | 0.79 | [0.51-1.22] | *0.292* |  |  | 0.98 | [0.63-1.53] | *0.963* |
| **Progression status(no)†** |  | 0.98 | [0.94-1.02] | *0.425* |  |  | 0.98 | [0.94-1.01] | *0.314* |
| **Adjuvant** |  |  |  |  |  |  |  |  |  |
| yes |  | 1 |  |  |  |  |  |  |  |
| no |  | 0.75 | [0.48-1.18] | *0.227* |  |  | _ | _ | *_* |
|  |  | **pain** |  |  |  |  | **Global Health** |  |  |
| **Treatment arms** | *116(68)* |  |  |  |  | *123(83)* |  |  | *0.17* |
| FOLFOX alone |  | 1 |  |  |  |  | 1 |  |  |
| FOLFOX + FOLFIRI | | 0.6 | [0.36-0.98] | *0.044* |  |  | 0.93 | [0.640-1.5] | *0.769* |
| **Progression status(no)†** |  | 0.99 | [0.95-1.03] | *0.783* |  |  | 0.98 | [0.95-1.02] | *0.465* |
| **Performance status** | |  |  |  |  |  |  |  |  |
| 0 |  | 1 |  |  |  |  |  |  |  |
| 1-2 |  | 1.47 | [0.79-2.79] | *0.209* |  |  | _ | _ | _ |

Table A3 continued

|  |  |  |  |  |  |  |  |  |  |
| --- | --- | --- | --- | --- | --- | --- | --- | --- | --- |
|  | **n** | **Hazard ratio** | **(95%°CI)** | **p** |  | **n** | **Hazard ratio** | **(95%°CI)** | **p** |
|  |  | **physical** |  |  |  |  | **diarrhea** |  |  |
| **Treatment arms** | *123(75)* |  |  |  |  | *120(74)* |  |  |  |
| FOLFOX alone |  | 1 |  |  |  |  | 1 |  |  |
| FOLFOX + FOLFIRI | | 1.02 | [0.63-1.63] | *0.926* |  |  | 0.97 | [0.61-1.55] | *0.915* |
| **Symptoms** |  |  |  |  |  |  |  |  |  |
| yes |  | 1 |  |  |  |  | 1 |  |  |
| no |  | 1.52 | [0.94-2.48] | *0.086* |  |  | 0.59 | [0.36-0.96] | *0.034* |
| **Progression status(no)†** |  | 0.98 | [0.94-1.02] | *0.491* |  |  | 0.99 | [0.95-1.03] | *0.683* |
| **Gender** |  |  |  |  |  |  |  |  |  |
| female |  | 1 |  |  |  |  |  |  |  |
| male |  | 0.64 | [0.39-1.05] | *0.083* |  |  | _ | _ | *_* |
|  |  | **apetite loss** |  |  |  |  |  | **Constipation** |  |
| **Treatment arms** | *116(75)* |  |  |  |  | *116(73)* |  |  |  |
| FOLFOX alone |  | 1 |  |  |  |  | 1 |  |  |
| FOLFOX + FOLFIRI | | 1.01 | [0.63-1.61] | *0.955* |  |  | 0.82 | [0.51-1.32] | *0.419* |
| **Symptoms** |  |  |  |  |  |  |  |  |  |
| yes |  | 1 |  |  |  |  | _ | _ | *_* |
| no |  | 1.33 | [0.81-2.19] | *0.254* |  |  |  |  |  |
| **Performance status** | |  |  |  |  |  |  |  |  |
| 0 |  | 1 |  |  |  |  | 1 |  |  |
| 1-2 |  | 1.77 | [0.98-3.17] | *0.054* |  |  | 1.44 | [0.79-2.64] | *0.225* |
| **Progression status(no)†** |  | 1 | [0.96-1.04] | *0.927* |  |  | 0.99 | [0.95-1.03] | *0.873* |
| **Delay between diagnostic and metastase** | | |  |  |  |  |  |  |  |
| 0.1-12 months | |  |  |  |  |  | 1 |  |  |
| simultanous | | _ | _ | _ |  |  | 0.82 | [0.47-1.44] | *0.505* |
| >12 months |  | _ | _ | _ |  |  | 0.62 | [0.33-1.19] | *0.159* |

**† : considered as time dependent variable for analyses.**

Table A3 continued

|  |  |  |  |  |  |  |  |  |  |
| --- | --- | --- | --- | --- | --- | --- | --- | --- | --- |
|  | **n** | **Hazard ratio** | **(95%°CI)** | **p** |  | **n** | **Hazard ratio** | **(95%°CI)** | **p** |
|  |  | **nausea** |  |  |  |  | **emotional** |  |  |
|  |  |  |  |  |  |  |  |  |  |
| **Treatment arms** | *120(88)* |  |  |  |  | *121(77)* |  |  |  |
| FOLFOX alone |  | 1 |  |  |  |  | 1 |  |  |
| FOLFOX + FOLFIRI | | 0.94 | [0.61-1.45] | *0.793* |  |  | 1.18 | [0.74-1.88] | *0.463* |
| **Symptoms** |  |  |  |  |  |  |  |  |  |
| yes |  | 1 |  |  |  |  | 1 |  |  |
| no |  | 1.35 | [0.85-2.13] | *0.193* |  |  | 1.2 | [0.75-1.93] | *0.441* |
| **BSA** |  |  |  |  |  |  |  |  |  |
| ≤1.73 |  | 1 |  |  |  |  |  |  |  |
| >1.73 |  | 1.74 | [1.03-2.91] | *0.036* |  |  | **dyspnea** |  |  |
| **Delay between diagnostic and metastase** | | |  |  |  |  |  |  |  |
| 0.1-12 months | | 1 |  |  |  |  | 1 |  |  |
| simultanous | | 0.77 | [0.47-1.28] | *0.324* |  |  | 0.64 | [0.38-1.10] | *0.113* |
| >12 months |  | 0.56 | [0.31-1.01] | *0.056* |  |  | 0.48 | [0.26-0.89] | *0.02* |
| **Progression status(no)†** |  | 0.97 | [0.93-1.02] | *0.337* |  |  | 0.98 | [0.95-1.03] | *0.619* |
| **Treatment arms** | |  |  |  |  |  |  |  |  |
| FOLFOX alone |  |  |  |  |  |  | 1 |  |  |
| FOLFOX + FOLFIRI | | _ | _ | *_* |  |  | 0.82 | [0.52-1.30] | *0.416* |
|  |  | **cognitive** |  |  |  |  |  | **Financial** |  |
| **Treatment arms** | *155(98)* |  |  |  |  | *121(63)* |  |  |  |
| FOLFOX alone |  | 1 |  |  |  |  | 1 |  |  |
| FOLFOX + FOLFIRI | | 0.85 | [0.52-1.37] | *0.515* |  |  | 0.76 | [0.42-1.37] | *0.368* |
| **Delay between diagnostic and metastase** | | |  |  |  |  |  |  |  |
| 0.1-12 months | | 1 |  |  |  |  | 1 |  |  |
| simultanous | | 0.77 | [0.43-1.36] | *0.376* |  |  | 1.58 | [0.86-2.91] | *0.139* |
| >12 months |  | 0.58 | [0.31-1.10] | *0.097* |  |  | 0.68 | [0.32-1.44] | *0.321* |
| **Performance status** | |  |  |  |  |  |  |  |  |
| 0 |  | 1 |  |  |  |  |  |  |  |
| 1-2 |  | 1.32 | [0.70-2.48] | *0.378* |  |  | _ | _ | *_* |
| **Progression status(no)†** |  | 0.99 | [0.95-1.03] | *0.687* |  |  | _ | _ | *_* |
| **Age** |  |  |  |  |  |  |  |  |  |
| <63 |  |  |  |  |  |  | 1 |  |  |
| >=63 |  |  |  |  |  |  | 0.61 | [0.31-1.20] | *0.155* |
|  |  |  |  |  |  |  |  |  |  |

**† : considered as time dependent variable for analyses.**

S6:Table A4: Univariate analysis of TUDD excluding death as event and of TTD for EORTC QLQ-C30 scales according to treatment arm

|  |  |  | **TTD** |  |  |  |  |  |  | **TUDD** |  |  |  |
| --- | --- | --- | --- | --- | --- | --- | --- | --- | --- | --- | --- | --- | --- |
|  | ***N*** | **Deterioration** | **Median** | **95% CI** | **HR [95% CI]** | ***P*-value** |  | ***N*** | **Deterioration** | **Median** | **95% CI** | **HR [95% CI]** | ***P-*value** |
| **Global health status** |  |  |  |  |  |  |  |  |  |  |  |  |  |
| FOLFOX4 | 45 | 18 | 6.63 | 3.7-NR |  |  |  | 45 | 16 | 6.63 | 3.9-NR |  |  |
| FOLFOX7 + FOLFIRI | 42 | 23 | 5.93 | 2.33-7.8 | 1.28 [0.69-2.39] | 0.429 |  | 42 | 20 | 6.56 | 5.4-8.23 | 1.15 [0.59-2.24] | 0.663 |
| **Physical functioning** |  |  |  |  |  |  |  |  |  |  |  |  |  |
| FOLFOX4 | 45 | 15 | 6.63 | 3.96-NR |  |  |  | 45 | 15 | 6.63 | 3.96-NR |  |  |
| FOLFOX7 + FOLFIRI | 44 | 18 | 7.56 | 6.16-9.93 | 1.03 [0.52-2.07] | 0.911 |  | 44 | 18 | 7.56 | 6.06-9.93 | 1.03 [0.52-2.07] |  |
| **Role functioning** |  |  |  |  |  |  |  |  |  |  |  |  |  |
| FOLFOX4 | 45 | 21 | 4.7 | 3.5-6.63 |  |  |  | 45 | 21 | 4.73 | 3.5-6.63 |  |  |
| FOLFOX7 + FOLFIRI | 42 | 20 | 6.16 | 2.33-NR | 0.92 [0.49-1.71] | 0.793 |  | 42 | 19 | 6.16 | 3.2-NR | 0.8 [0.42-1.51] | 0.501 |
| **Emotional functioning** |  |  |  |  |  |  |  |  |  |  |  |  |  |
| FOLFOX4 | 44 | 12 | NR | 4.06-NR |  |  |  | 44 | 12 | NR | 4.06-NR |  |  |
| FOLFOX7 + FOLFIRI | 43 | 22 | 5.93 | 3.96-9.7 | 1.67 [0.82-3.38] | 0.154 |  | 43 | 21 | 6.16 | 4-9.7 | 1.55 [0.76-3.16] | 0.225 |
| **Cognitive functioning** |  |  |  |  |  |  |  |  |  |  |  |  |  |
| FOLFOX4 | 45 | 16 | 6.63 | 4.06-NR |  |  |  | 45 | 16 | 6.63 | 4.06-NR |  |  |
| FOLFOX7 + FOLFIRI | 43 | 19 | 6.16 | 4.66-NR | 0.84 [0.43-1.66] | 0.636 |  | 43 | 19 | 6.16 | 4.66-NR | 0.84 [0.43-1.66] | 0.636 |
| **Social functioning** |  |  |  |  |  |  |  |  |  |  |  |  |  |
| FOLFOX4 | 44 | 18 | 5.53 | 3.76-NR |  |  |  | 44 | 17 | 6.23 | 3.96-NR |  |  |
| FOLFOX7 + FOLFIRI | 43 | 23 | 5.43 | 2.83-NR | 1.08 [0.57-2.03] | 0.803 |  | 43 | 23 | 5.93 | 3.86-6.3 | 1.16 [0.61-2.19] | 0.647 |
| **Fatigue** |  |  |  |  |  |  |  |  |  |  |  |  |  |
| FOLFOX4 alone | 44 | 27 | 3.7 | 2.8-5.56 |  |  |  | 45 | 25 | 4.66 | 3.2-6.63 |  |  |
| FOLFOX7 + FOLFIRI | 42 | 31 | 3.06 | 2.16-5.13 | 1.22 [0.72-2.07] | 0.44 |  | 43 | 32 | 3.96 | 2.33-5.1 | 1.51 [0.88-2.56] | 0.128 |
| **Nausea** |  |  |  |  |  |  |  |  |  |  |  |  |  |
| FOLFOX4 | 45 | 24 | 3.96 | 3.26-6.63 |  |  |  | 45 | 24 | 3.96 | 3.2-6.63 |  |  |
| FOLFOX7 + FOLFIRI | 43 | 27 | 5.9 | 2.83-6.63 | 0.91 [0.52-1.61] | 0.768 |  | 43 | 26 | 5.93 | 3.06-7.2 | 0.83 [0.47-1.46] | 0.523 |

Table A4 continued

|  |  |  | **TTD** |  |  |  |  |  |  | **TUDD** |  |  |  |
| --- | --- | --- | --- | --- | --- | --- | --- | --- | --- | --- | --- | --- | --- |
|  | **Total** | **Deterioration** | **Median** | **95% CI** | **HR [95% CI]** | **P-value** |  | **Total** | **Deterioration** | **Median** | **95% CI** | **HR [95 % CI]** | **P-value** |
| **Pain** |  |  |  |  |  |  |  |  |  |  |  |  |  |
| FOLFOX4 | 46 | 18 | 6.63 | 5.56-NR |  |  |  | 45 | 17 | 6.63 | 3.8-NR |  |  |
| FOLFOX7 + FOLFIRI | 43 | 11 | 7.56 | 6.6-NR | 0.49 [0.22-1.06] | 0.073 |  | 43 | 10 | 7.56 | 6.06-NR | 0.41 [0.18-0.91] | 0.03 |
| **Dyspnea** |  |  |  |  |  |  |  |  |  |  |  |  |  |
| FOLFOX4 | 43 | 19 | 5.5 | 3.7-NR |  |  |  | 43 | 19 | 5.5 | 3.7-NR |  |  |
| FOLFOX7 + FOLFIRI | 43 | 15 | 9.7 | 5.43-NR | 0.68 [0.34-1.35] | 0.274 |  | 43 | 15 | 9.7 | 5.4-NR | 0.68 [0.34-1.35] | 0.274 |
| **Insomnia** |  |  |  |  |  |  |  |  |  |  |  |  |  |
| FOLFOX4 | 45 | 14 | NR | 3.96-NR |  |  |  | 45 | 13 | NR | 5.56-NR |  |  |
| FOLFOX7 + FOLFIRI | 43 | 12 | NR | 6.06-NR | 0.69 [0.32-1.51] | 0.36 |  | 43 | 11 | NR | 6.06-NR | 0.69 [0.30-1.55] | 0.376 |
| **Appetite loss** |  |  |  |  |  |  |  |  |  |  |  |  |  |
| FOLFOX4 | 45 | 18 | 6.63 | 4.66-NR |  |  |  | 45 | 18 | 6.63 | 4.6-NR |  |  |
| FOLFOX7 + FOLFIRI | 43 | 21 | 6.63 | 2.53-NR | 1.24 [0.65-2.36] | 0.497 |  | 43 | 21 | 6.63 | 2.53-NR | 1.22 [0.64-2.32] | 0.528 |
| **Constipation** |  |  |  |  |  |  |  |  |  |  |  |  |  |
| FOLFOX4 | 44 | 13 | 7.26 | 5.73 |  |  |  | 44 | 13 | 7.26 | 5.7-NR |  |  |
| FOLFOX7 + FOLFIRI | 43 | 15 | 7.26 | 5.93 | 1.08 [0.51-2.29] | 0.822 |  | 43 | 15 | 7.26 | 5.9-NR | 1.05 [0.49-2.21] | 0.895 |
| **Diarrhea** |  |  |  |  |  |  |  |  |  |  |  |  |  |
| FOLFOX4 | 44 | 15 | NR | 3.2-NR |  |  |  | 44 | 15 | NR | 3.26-NR |  |  |
| FOLFOX7 + FOLFIRI | 40 | 16 | 6.63 | 5-NR | 0.95 [0.47-1.92] | 0.891 |  | 40 | 16 | 6.63 | 5.4-NR | 0.91 [0.45-1.85] | 0.81 |
| **Financial difficulties** |  |  |  |  |  |  |  |  |  |  |  |  |  |
| FOLFOX4 | 43 | 7 | NR | 6.23-NR |  |  |  | 43 | 7 | NR | 6.2-NR |  |  |
| FOLFOX7 + FOLFIRI | 42 | 9 | 9.93 | 7.43-NR | 1.04 [0.38-2.81] | 0.93 |  | 42 | 9 | 9.93 | 7.43-NR | 0.99 [0.36-2.67] | 0.988 |
