# Supplementary material for: Time to Definitive Health-Related Quality of Life Score Deterioration in Patients with Resectable Metastatic Colorectal Cancer Treated with FOLFOX4 versus Sequential Dose-Dense FOLFOX7 followed by FOLFIRI: The MIROX Randomized Phase III Trial
Source: PLoS One. 2016 Jun 16;11(6):e0157067. doi: 10.1371/journal.pone.0157067 (PMC4910973; doi:10.1371/journal.pone.0157067)
Supplement: S2 Table — (DOC) [file pone.0157067.s005.doc]

S2: Table A2: Baseline characteristics of patients enrolled on the MIROX study

**^*^**Fisher exact test

|  | **Patients with HRQoL data**  ***N* = 171** | **Patients without HRQoL data**  ***N* = 113** | **Total** | ***P*-value** |
| --- | --- | --- | --- | --- |
| Age, n (%) |  |  |  | 0.468 |
| <63 | 90 (53) | 54 (48) | 144 (51) |  |
| >=63 | 81 (47) | 59 (52) | 140 (49) |  |
| Gender, n (%) |  |  |  | 0.029 |
| Female | 48 (28) | 46 (41) | 94 (33) |  |
| Male | 123 (72) | 67 (59) | 190 (67) |  |
| Treatment arms, n (%) |  |  |  | 0.628 |
| FOLFOX4 | 83 (49) | 59 (52) | 142 (50) |  |
| FOLFOX7 + FOLFIRI | 88 (51) | 54 (48) | 142 (50) |  |
| Adjuvant chemotherapy, n (%) |  |  |  | 0.903 |
| Yes | 71 (42) | 46 (41) | 117 (41) |  |
| No | 100 (58) | 67 (59) | 167 (59) |  |
| Tumor site, n (%) |  |  |  | 0.602 |
| Colon | 115 (67) | 79 (70) | 194 (68) |  |
| Rectum | 56 (33) | 33 (29) | 89 (31) |  |
| Unknown | 0 (0) | 1 (1) | 1 (> 1) |  |
| Body surface area |  |  |  | 0.235 |
| ≤1.73 mg/mL | 49 (29) | 39 (35) | 88 (31) |  |
| >1.73 mg/mL | 120 (70) | 69 (61) | 189 (67) |  |
| Unknown | 2 (1) | 5 (4) | 7 |  |
| Symptoms |  |  |  | 0.017 |
| Yes | 62 (36) | 25 (22) | 87 (31) |  |
| No | 108 (63) | 86 (76) | 194 (68) |  |
| Unknown | 1 (1) | 2 (2) | 3 (1) |  |
| Performance status |  |  |  | 0.891 |
| 0 | 115 (67) | 73 (65) | 188 (66) |  |
| 1-2 | 51 (30) | 30 (26) | 81 (29) |  |
| Unknown | 5 (3) | 10 (9) | 15 (5) |  |
| Delay between diagnostic of the primary tumor and metastasis |  |  |  | 0.653 |
| Simultaneous | 53 (31) | 34 (30) | 87 (30) |  |
| 0.1-12 months | 67 (39) | 39 (34) | 106 (37) |  |
| >12 months | 51 (30) | 39 (34) | 90 (32) |  |
| Unknown | 0 (0) | 1 (1) | 1 (> 1) |  |
