# Supplementary material for: Time to Definitive Health-Related Quality of Life Score Deterioration in Patients with Resectable Metastatic Colorectal Cancer Treated with FOLFOX4 versus Sequential Dose-Dense FOLFOX7 followed by FOLFIRI: The MIROX Randomized Phase III Trial
Source: PLoS One. 2016 Jun 16;11(6):e0157067. doi: 10.1371/journal.pone.0157067 (PMC4910973; doi:10.1371/journal.pone.0157067)
Supplement: S3 Table — (DOCX) [file pone.0157067.s006.docx]

S2 Table A3: multivariate Cox analyses of TUDD analyses for QoL scores

|  |  |  |  |  |  |  |  |  |  |
| --- | --- | --- | --- | --- | --- | --- | --- | --- | --- |
|  | **n** | **Hazard ratio** | **(95%°CI)** | **p** |  | **n** | **Hazard ratio** | **(95%°CI)** | **p** |
|  |  | **fatigue** |  |  |  |  | **insomnia** |  |  |
| **Treatmentarms** | *124(97)* |  |  |  |  | *124(69)* |  |  |  |
| FOLFOX alone |  | 1 |  |  |  |  | 1 |  |  |
| FOLFOX + FOLFIRI | | 1.12 | [0.74-1.69] | *0.584* |  |  | 0.82 | [0.51-1.34] | *0.448* |
| **Progression status(no)†** |  | 0.97 | [0.94-1.01] | *0.211* |  |  | 0.98 | [0.95-1.02] | *0.574* |
| **Localisation** |  |  |  |  |  |  |  |  |  |
| Colon |  | 1 |  |  |  |  |  |  |  |
| Rectum |  | 0.69 | [0.44-1.07] | *0.102* |  |  | _ | _ | *_* |
|  |  | **Role** |  |  |  |  | **social** |  |  |
| **Treatmentarms** | *123(85)* |  |  |  |  | *122(85)* |  |  |  |
| FOLFOX alone |  | 1 |  |  |  |  | 1 |  |  |
| FOLFOX + FOLFIRI | | 0.79 | [0.51-1.22] | *0.292* |  |  | 0.98 | [0.63-1.53] | *0.963* |
| **Progression status(no)†** |  | 0.98 | [0.94-1.02] | *0.425* |  |  | 0.98 | [0.94-1.01] | *0.314* |
| **Adjuvant** |  |  |  |  |  |  |  |  |  |
| yes |  | 1 |  |  |  |  |  |  |  |
| no |  | 0.75 | [0.48-1.18] | *0.227* |  |  | _ | _ | *_* |
|  |  | **pain** |  |  |  |  | **Global Health** |  |  |
| **Treatmentarms** | *116(68)* |  |  |  |  | *123(83)* |  |  | *0.17* |
| FOLFOX alone |  | 1 |  |  |  |  | 1 |  |  |
| FOLFOX + FOLFIRI | | 0.6 | [0.36-0.98] | *0.044* |  |  | 0.93 | [0.640-1.5] | *0.769* |
| **Progression status(no)†** |  | 0.99 | [0.95-1.03] | *0.783* |  |  | 0.98 | [0.95-1.02] | *0.465* |
| **Performance status** | |  |  |  |  |  |  |  |  |
| 0 |  | 1 |  |  |  |  |  |  |  |
| 1-2 |  | 1.47 | [0.79-2.79] | *0.209* |  |  | _ | _ | _ |

Table A3 continued

|  |  |  |  |  |  |  |  |  |  |
| --- | --- | --- | --- | --- | --- | --- | --- | --- | --- |
|  | **n** | **Hazard ratio** | **(95%°CI)** | **p** |  | **n** | **Hazard ratio** | **(95%°CI)** | **p** |
|  |  | **physical** |  |  |  |  | **diarrhea** |  |  |
| **Treatmentarms** | *123(75)* |  |  |  |  | *120(74)* |  |  |  |
| FOLFOX alone |  | 1 |  |  |  |  | 1 |  |  |
| FOLFOX + FOLFIRI | | 1.02 | [0.63-1.63] | *0.926* |  |  | 0.97 | [0.61-1.55] | *0.915* |
| **Symptoms** |  |  |  |  |  |  |  |  |  |
| yes |  | 1 |  |  |  |  | 1 |  |  |
| no |  | 1.52 | [0.94-2.48] | *0.086* |  |  | 0.59 | [0.36-0.96] | *0.034* |
| **Progression status(no)†** |  | 0.98 | [0.94-1.02] | *0.491* |  |  | 0.99 | [0.95-1.03] | *0.683* |
| **Gender** |  |  |  |  |  |  |  |  |  |
| female |  | 1 |  |  |  |  |  |  |  |
| male |  | 0.64 | [0.39-1.05] | *0.083* |  |  | _ | _ | *_* |
|  |  | **apetiteloss** |  |  |  |  |  | **Constipation** |  |
| **Treatmentarms** | *116(75)* |  |  |  |  | *116(73)* |  |  |  |
| FOLFOX alone |  | 1 |  |  |  |  | 1 |  |  |
| FOLFOX + FOLFIRI | | 1.01 | [0.63-1.61] | *0.955* |  |  | 0.82 | [0.51-1.32] | *0.419* |
| **Symptoms** |  |  |  |  |  |  |  |  |  |
| yes |  | 1 |  |  |  |  | _ | _ | *_* |
| no |  | 1.33 | [0.81-2.19] | *0.254* |  |  |  |  |  |
| **Performance status** | |  |  |  |  |  |  |  |  |
| 0 |  | 1 |  |  |  |  | 1 |  |  |
| 1-2 |  | 1.77 | [0.98-3.17] | *0.054* |  |  | 1.44 | [0.79-2.64] | *0.225* |
| **Progression status(no)†** |  | 1 | [0.96-1.04] | *0.927* |  |  | 0.99 | [0.95-1.03] | *0.873* |
| **Delay between diagnostic and metastase** | | |  |  |  |  |  |  |  |
| 0.1-12 months | |  |  |  |  |  | 1 |  |  |
| simultanous | | _ | _ | _ |  |  | 0.82 | [0.47-1.44] | *0.505* |
| >12 months |  | _ | _ | _ |  |  | 0.62 | [0.33-1.19] | *0.159* |

**† : considered as time dependent variable for analyses.**

Table A3 continued

|  |  |  |  |  |  |  |  |  |  |
| --- | --- | --- | --- | --- | --- | --- | --- | --- | --- |
|  | **n** | **Hazard ratio** | **(95%°CI)** | **p** |  | **n** | **Hazard ratio** | **(95%°CI)** | **p** |
|  |  | **nausea** |  |  |  |  | **emotional** |  |  |
|  |  |  |  |  |  |  |  |  |  |
| **Treatmentarms** | *120(88)* |  |  |  |  | *121(77)* |  |  |  |
| FOLFOX alone |  | 1 |  |  |  |  | 1 |  |  |
| FOLFOX + FOLFIRI | | 0.94 | [0.61-1.45] | *0.793* |  |  | 1.18 | [0.74-1.88] | *0.463* |
| **Symptoms** |  |  |  |  |  |  |  |  |  |
| yes |  | 1 |  |  |  |  | 1 |  |  |
| no |  | 1.35 | [0.85-2.13] | *0.193* |  |  | 1.2 | [0.75-1.93] | *0.441* |
| **BSA** |  |  |  |  |  |  |  |  |  |
| ≤1.73 |  | 1 |  |  |  |  |  |  |  |
| >1.73 |  | 1.74 | [1.03-2.91] | *0.036* |  |  | **dyspnea** |  |  |
| **Delay between diagnostic and metastase** | | |  |  |  |  |  |  |  |
| 0.1-12 months | | 1 |  |  |  |  | 1 |  |  |
| simultanous | | 0.77 | [0.47-1.28] | *0.324* |  |  | 0.64 | [0.38-1.10] | *0.113* |
| >12 months |  | 0.56 | [0.31-1.01] | *0.056* |  |  | 0.48 | [0.26-0.89] | *0.02* |
| **Progression status(no)†** |  | 0.97 | [0.93-1.02] | *0.337* |  |  | 0.98 | [0.95-1.03] | *0.619* |
| **Treatmentarms** | |  |  |  |  |  |  |  |  |
| FOLFOX alone |  |  |  |  |  |  | 1 |  |  |
| FOLFOX + FOLFIRI | | _ | _ | *_* |  |  | 0.82 | [0.52-1.30] | *0.416* |
|  |  | **cognitive** |  |  |  |  |  | **Financial** |  |
| **Treatmentarms** | *155(98)* |  |  |  |  | *121(63)* |  |  |  |
| FOLFOX alone |  | 1 |  |  |  |  | 1 |  |  |
| FOLFOX + FOLFIRI | | 0.85 | [0.52-1.37] | *0.515* |  |  | 0.76 | [0.42-1.37] | *0.368* |
| **Delay between diagnostic and metastase** | | |  |  |  |  |  |  |  |
| 0.1-12 months | | 1 |  |  |  |  | 1 |  |  |
| simultanous | | 0.77 | [0.43-1.36] | *0.376* |  |  | 1.58 | [0.86-2.91] | *0.139* |
| >12 months |  | 0.58 | [0.31-1.10] | *0.097* |  |  | 0.68 | [0.32-1.44] | *0.321* |
| **Performance status** | |  |  |  |  |  |  |  |  |
| 0 |  | 1 |  |  |  |  |  |  |  |
| 1-2 |  | 1.32 | [0.70-2.48] | *0.378* |  |  | _ | _ | *_* |
| **Progression status(no)†** |  | 0.99 | [0.95-1.03] | *0.687* |  |  | _ | _ | *_* |
| **Age** |  |  |  |  |  |  |  |  |  |
| <63 |  |  |  |  |  |  | 1 |  |  |
| >=63 |  |  |  |  |  |  | 0.61 | [0.31-1.20] | *0.155* |
|  |  |  |  |  |  |  |  |  |  |

**† : considered as time dependent variable for analyses.**
