# Supplementary material for: Time to Definitive Health-Related Quality of Life Score Deterioration in Patients with Resectable Metastatic Colorectal Cancer Treated with FOLFOX4 versus Sequential Dose-Dense FOLFOX7 followed by FOLFIRI: The MIROX Randomized Phase III Trial
Source: PLoS One. 2016 Jun 16;11(6):e0157067. doi: 10.1371/journal.pone.0157067 (PMC4910973; doi:10.1371/journal.pone.0157067)
Supplement: S4 Table — (DOCX) [file pone.0157067.s007.docx]

S2 TableA4: Univariate analysis of TUDD excluding death as event and of TTD for EORTC QLQ-C30 scales according to treatment arm

|  |  |  | **TTD** |  |  |  |  |  |  | **TUDD** |  |  |  |
| --- | --- | --- | --- | --- | --- | --- | --- | --- | --- | --- | --- | --- | --- |
|  | ***N*** | **Deterioration** | **Median** | **95% CI** | **HR [95% CI]** | ***P*-value** |  | ***N*** | **Deterioration** | **Median** | **95% CI** | **HR [95% CI]** | ***P-*value** |
| **Global health status** |  |  |  |  |  |  |  |  |  |  |  |  |  |
| FOLFOX4 | 45 | 18 | 6.63 | 3.7-NR |  |  |  | 45 | 16 | 6.63 | 3.9-NR |  |  |
| FOLFOX7 + FOLFIRI | 42 | 23 | 5.93 | 2.33-7.8 | 1.28[0.69-2.39] | 0.429 |  | 42 | 20 | 6.56 | 5.4-8.23 | 1.15[0.59-2.24] | 0.663 |
| **Physical functioning** |  |  |  |  |  |  |  |  |  |  |  |  |  |
| FOLFOX4 | 45 | 15 | 6.63 | 3.96-NR |  |  |  | 45 | 15 | 6.63 | 3.96-NR |  |  |
| FOLFOX7 + FOLFIRI | 44 | 18 | 7.56 | 6.16-9.93 | 1.03[0.52-2.07] | 0.911 |  | 44 | 18 | 7.56 | 6.06-9.93 | 1.03[0.52-2.07] |  |
| **Role functioning** |  |  |  |  |  |  |  |  |  |  |  |  |  |
| FOLFOX4 | 45 | 21 | 4.7 | 3.5-6.63 |  |  |  | 45 | 21 | 4.73 | 3.5-6.63 |  |  |
| FOLFOX7 + FOLFIRI | 42 | 20 | 6.16 | 2.33-NR | 0.92[0.49-1.71] | 0.793 |  | 42 | 19 | 6.16 | 3.2-NR | 0.8[0.42-1.51] | 0.501 |
| **Emotional functioning** |  |  |  |  |  |  |  |  |  |  |  |  |  |
| FOLFOX4 | 44 | 12 | NR | 4.06-NR |  |  |  | 44 | 12 | NR | 4.06-NR |  |  |
| FOLFOX7 + FOLFIRI | 43 | 22 | 5.93 | 3.96-9.7 | 1.67[0.82-3.38] | 0.154 |  | 43 | 21 | 6.16 | 4-9.7 | 1.55[0.76-3.16] | 0.225 |
| **Cognitive functioning** |  |  |  |  |  |  |  |  |  |  |  |  |  |
| FOLFOX4 | 45 | 16 | 6.63 | 4.06-NR |  |  |  | 45 | 16 | 6.63 | 4.06-NR |  |  |
| FOLFOX7 + FOLFIRI | 43 | 19 | 6.16 | 4.66-NR | 0.84[0.43-1.66] | 0.636 |  | 43 | 19 | 6.16 | 4.66-NR | 0.84[0.43-1.66] | 0.636 |
| **Social functioning** |  |  |  |  |  |  |  |  |  |  |  |  |  |
| FOLFOX4 | 44 | 18 | 5.53 | 3.76-NR |  |  |  | 44 | 17 | 6.23 | 3.96-NR |  |  |
| FOLFOX7 + FOLFIRI | 43 | 23 | 5.43 | 2.83-NR | 1.08[0.57-2.03] | 0.803 |  | 43 | 23 | 5.93 | 3.86-6.3 | 1.16[0.61-2.19] | 0.647 |
| **Fatigue** |  |  |  |  |  |  |  |  |  |  |  |  |  |
| FOLFOX4 alone | 44 | 27 | 3.7 | 2.8-5.56 |  |  |  | 45 | 25 | 4.66 | 3.2-6.63 |  |  |
| FOLFOX7 + FOLFIRI | 42 | 31 | 3.06 | 2.16-5.13 | 1.22[0.72-2.07] | 0.44 |  | 43 | 32 | 3.96 | 2.33-5.1 | 1.51[0.88-2.56] | 0.128 |
| **Nausea** |  |  |  |  |  |  |  |  |  |  |  |  |  |
| FOLFOX4 | 45 | 24 | 3.96 | 3.26-6.63 |  |  |  | 45 | 24 | 3.96 | 3.2-6.63 |  |  |
| FOLFOX7 + FOLFIRI | 43 | 27 | 5.9 | 2.83-6.63 | 0.91[0.52-1.61] | 0.768 |  | 43 | 26 | 5.93 | 3.06-7.2 | 0.83[0.47-1.46] | 0.523 |

Table A4 continued

|  |  |  | **TTD** |  |  |  |  |  |  | **TUDD** |  |  |  |
| --- | --- | --- | --- | --- | --- | --- | --- | --- | --- | --- | --- | --- | --- |
|  | **Total** | **Deterioration** | **Median** | **95% CI** | **HR [95% CI]** | **P-value** |  | **Total** | **Deterioration** | **Median** | **95% CI** | **HR [95 % CI]** | **P-value** |
| **Pain** |  |  |  |  |  |  |  |  |  |  |  |  |  |
| FOLFOX4 | 46 | 18 | 6.63 | 5.56-NR |  |  |  | 45 | 17 | 6.63 | 3.8-NR |  |  |
| FOLFOX7 + FOLFIRI | 43 | 11 | 7.56 | 6.6-NR | 0.49[0.22-1.06] | 0.073 |  | 43 | 10 | 7.56 | 6.06-NR | 0.41[0.18-0.91] | 0.03 |
| **Dyspnea** |  |  |  |  |  |  |  |  |  |  |  |  |  |
| FOLFOX4 | 43 | 19 | 5.5 | 3.7-NR |  |  |  | 43 | 19 | 5.5 | 3.7-NR |  |  |
| FOLFOX7 + FOLFIRI | 43 | 15 | 9.7 | 5.43-NR | 0.68 [0.34-1.35] | 0.274 |  | 43 | 15 | 9.7 | 5.4-NR | 0.68[0.34-1.35] | 0.274 |
| **Insomnia** |  |  |  |  |  |  |  |  |  |  |  |  |  |
| FOLFOX4 | 45 | 14 | NR | 3.96-NR |  |  |  | 45 | 13 | NR | 5.56-NR |  |  |
| FOLFOX7 + FOLFIRI | 43 | 12 | NR | 6.06-NR | 0.69[0.32-1.51] | 0.36 |  | 43 | 11 | NR | 6.06-NR | 0.69[0.30-1.55] | 0.376 |
| **Appetite loss** |  |  |  |  |  |  |  |  |  |  |  |  |  |
| FOLFOX4 | 45 | 18 | 6.63 | 4.66-NR |  |  |  | 45 | 18 | 6.63 | 4.6-NR |  |  |
| FOLFOX7 + FOLFIRI | 43 | 21 | 6.63 | 2.53-NR | 1.24[0.65-2.36] | 0.497 |  | 43 | 21 | 6.63 | 2.53-NR | 1.22 [0.64-2.32] | 0.528 |
| **Constipation** |  |  |  |  |  |  |  |  |  |  |  |  |  |
| FOLFOX4 | 44 | 13 | 7.26 | 5.73 |  |  |  | 44 | 13 | 7.26 | 5.7-NR |  |  |
| FOLFOX7 + FOLFIRI | 43 | 15 | 7.26 | 5.93 | 1.08[0.51-2.29] | 0.822 |  | 43 | 15 | 7.26 | 5.9-NR | 1.05[0.49-2.21] | 0.895 |
| **Diarrhea** |  |  |  |  |  |  |  |  |  |  |  |  |  |
| FOLFOX4 | 44 | 15 | NR | 3.2-NR |  |  |  | 44 | 15 | NR | 3.26-NR |  |  |
| FOLFOX7 + FOLFIRI | 40 | 16 | 6.63 | 5-NR | 0.95[0.47-1.92] | 0.891 |  | 40 | 16 | 6.63 | 5.4-NR | 0.91[0.45-1.85] | 0.81 |
| **Financial difficulties** |  |  |  |  |  |  |  |  |  |  |  |  |  |
| FOLFOX4 | 43 | 7 | NR | 6.23-NR |  |  |  | 43 | 7 | NR | 6.2-NR |  |  |
| FOLFOX7 + FOLFIRI | 42 | 9 | 9.93 | 7.43-NR | 1.04[0.38-2.81] | 0.93 |  | 42 | 9 | 9.93 | 7.43-NR | 0.99[0.36-2.67] | 0.988 |
